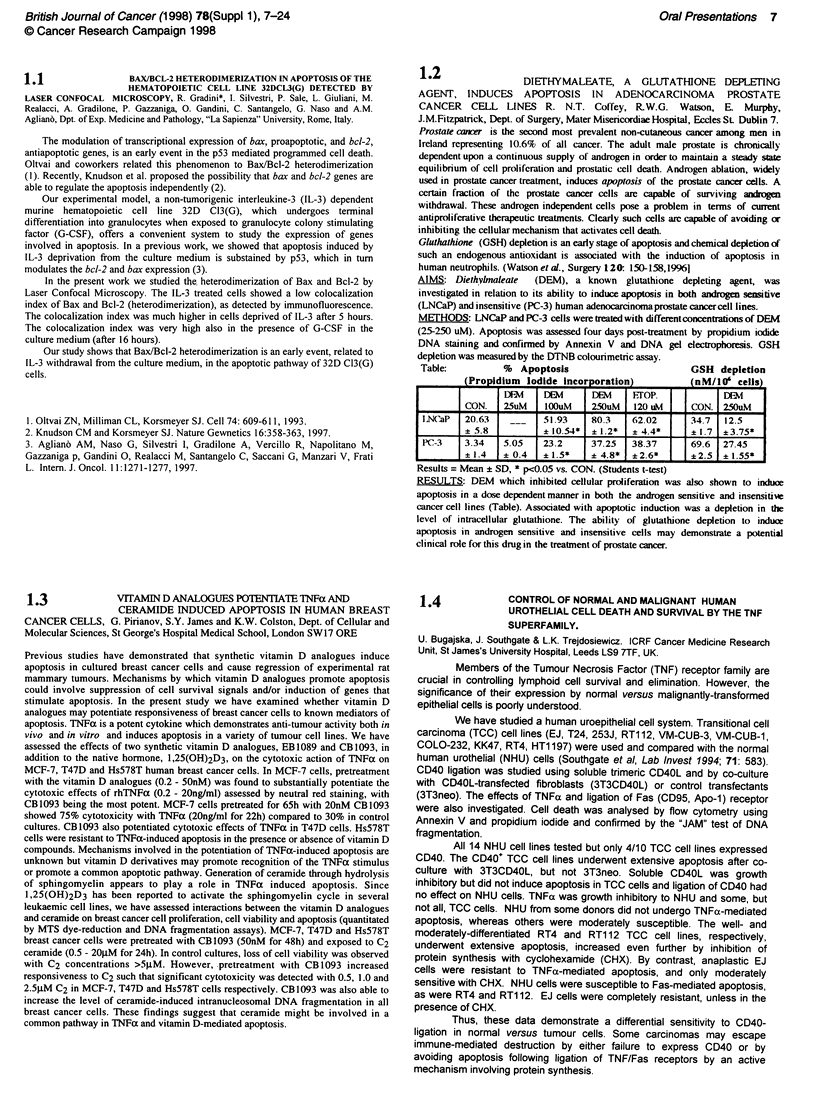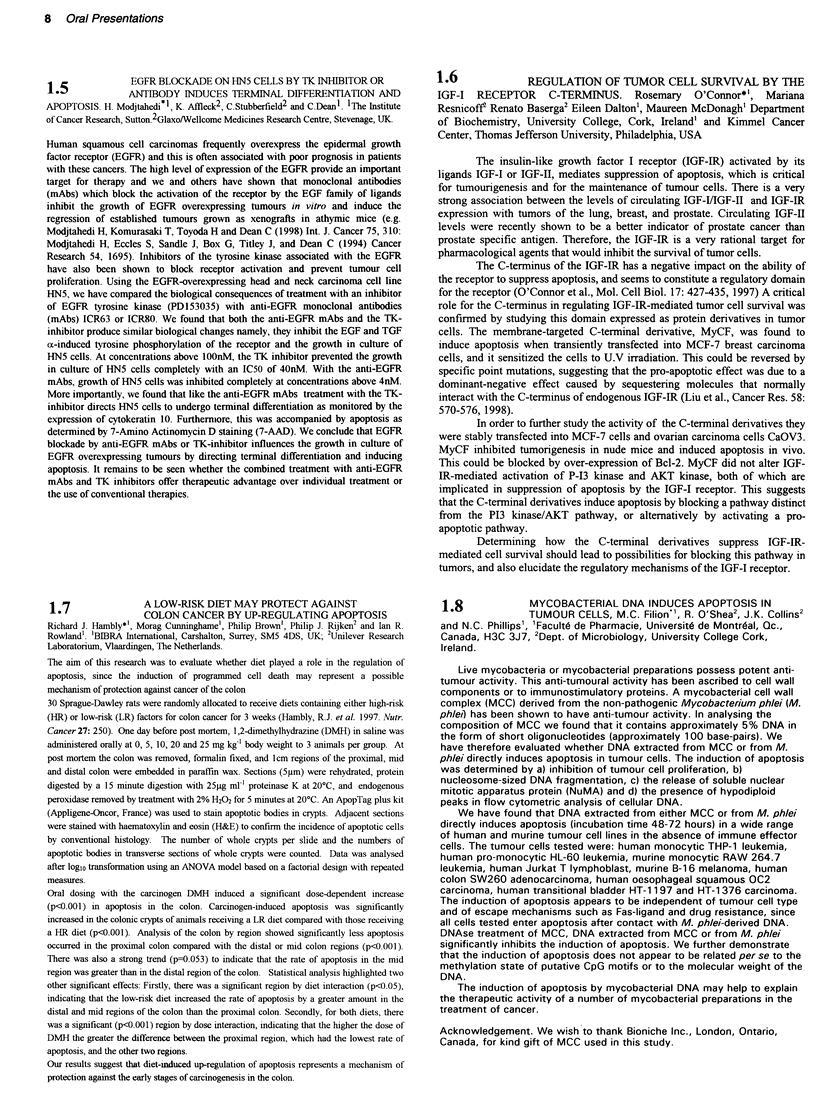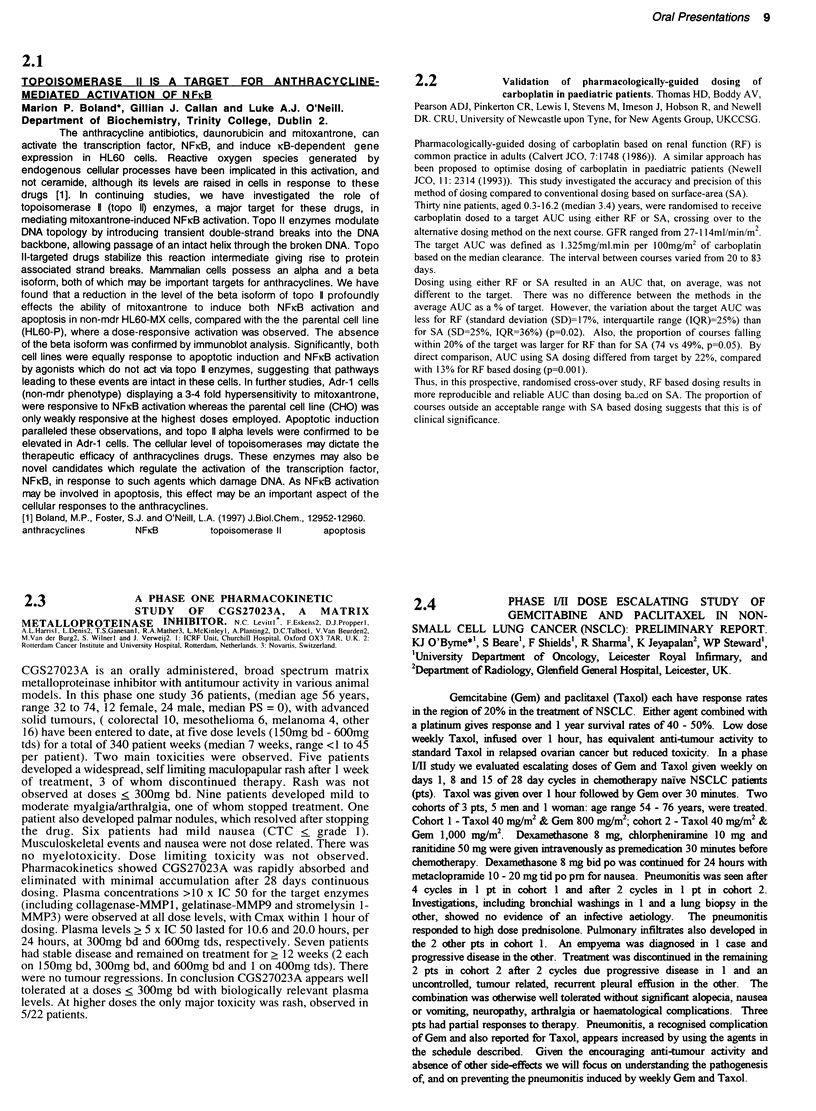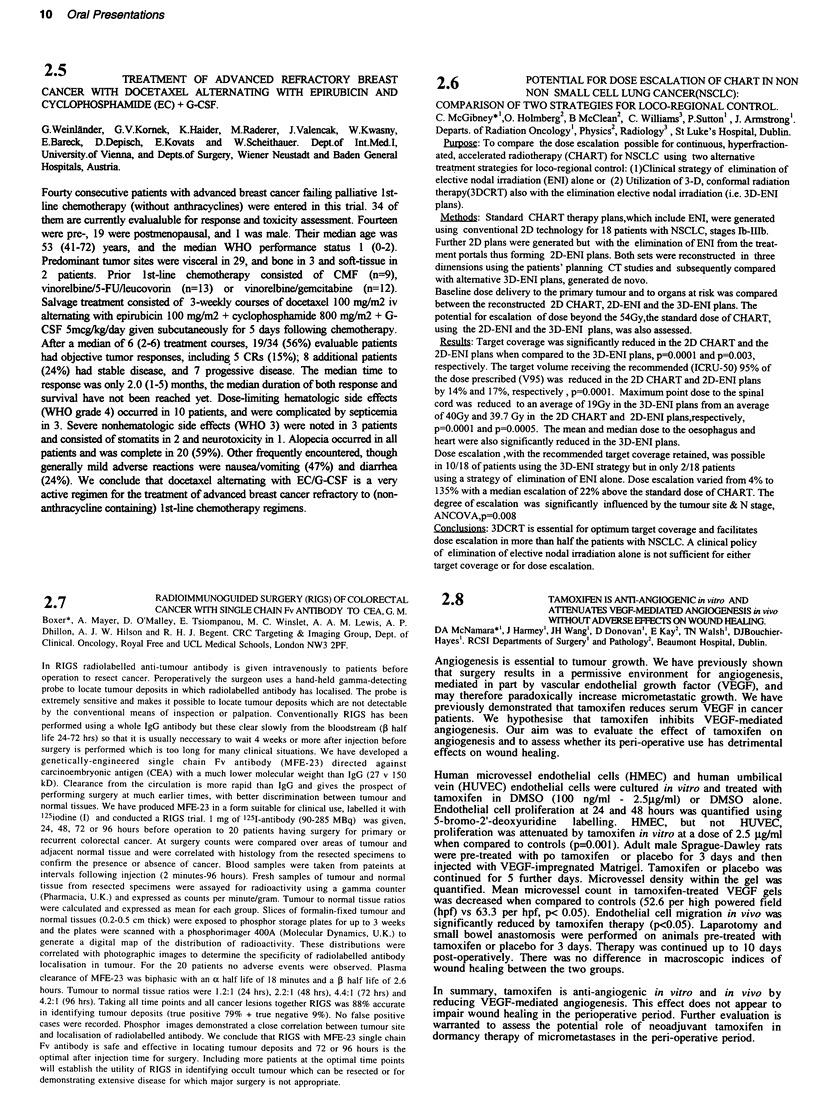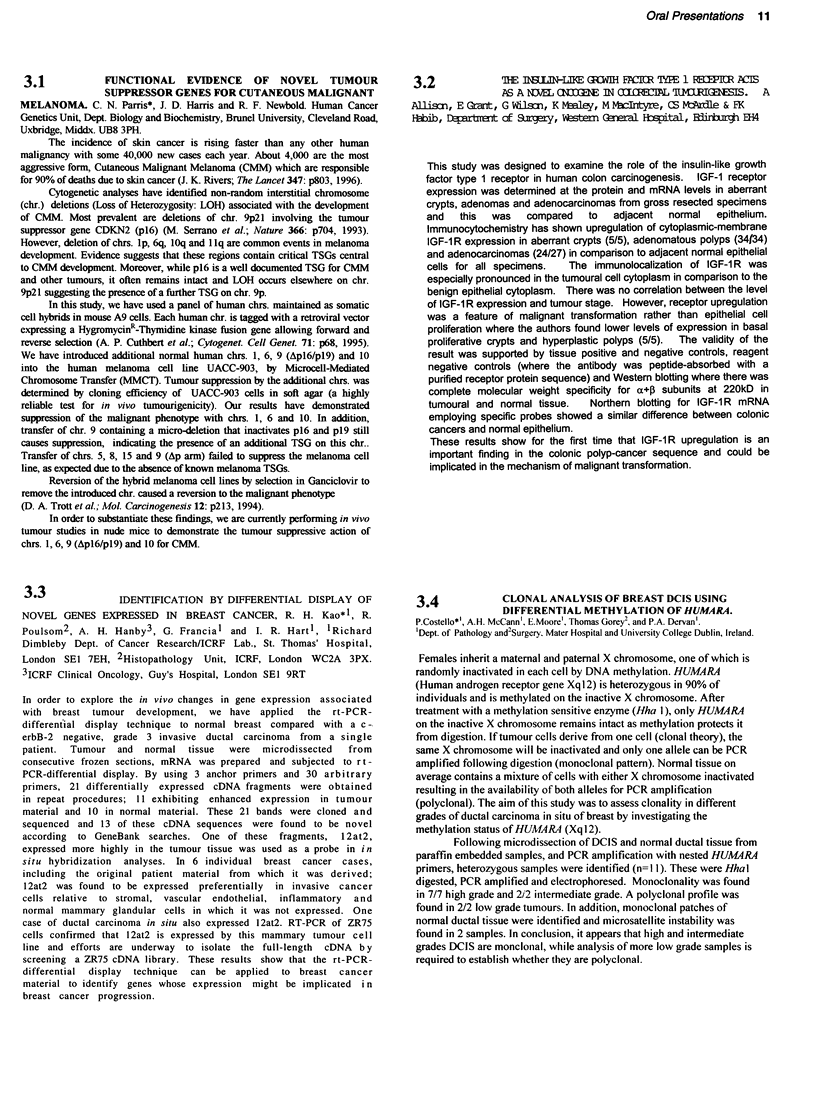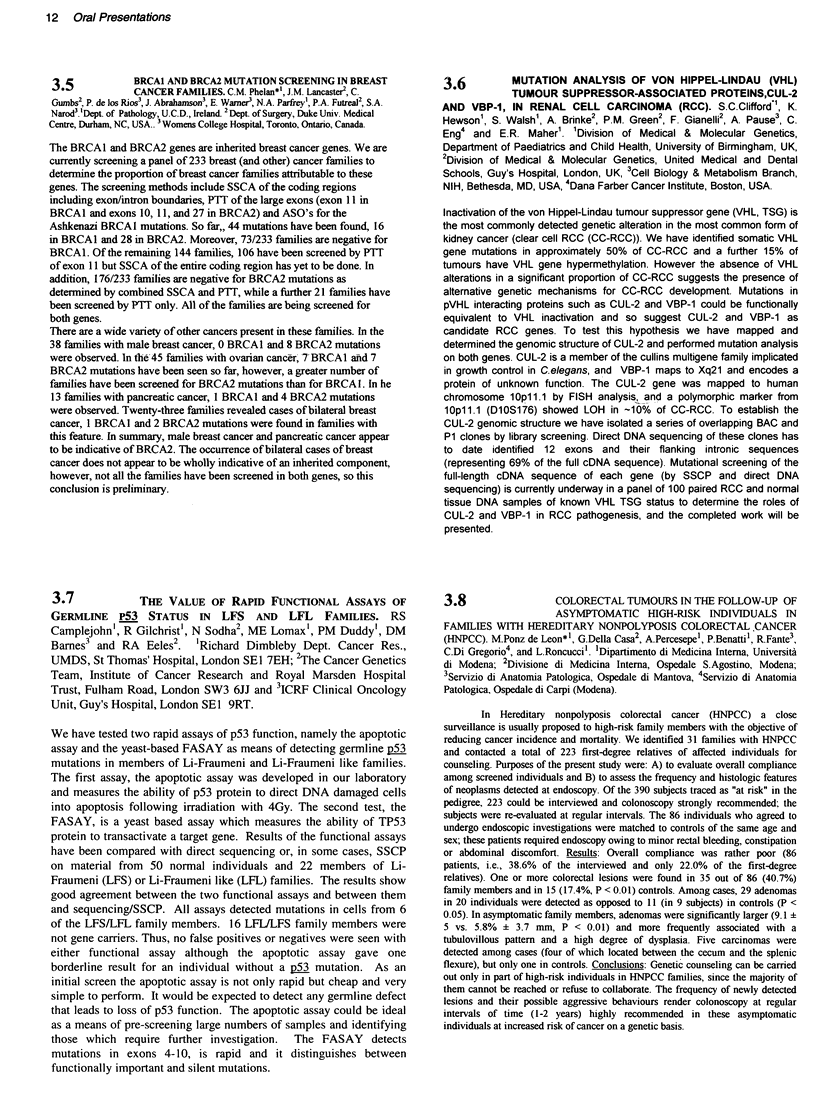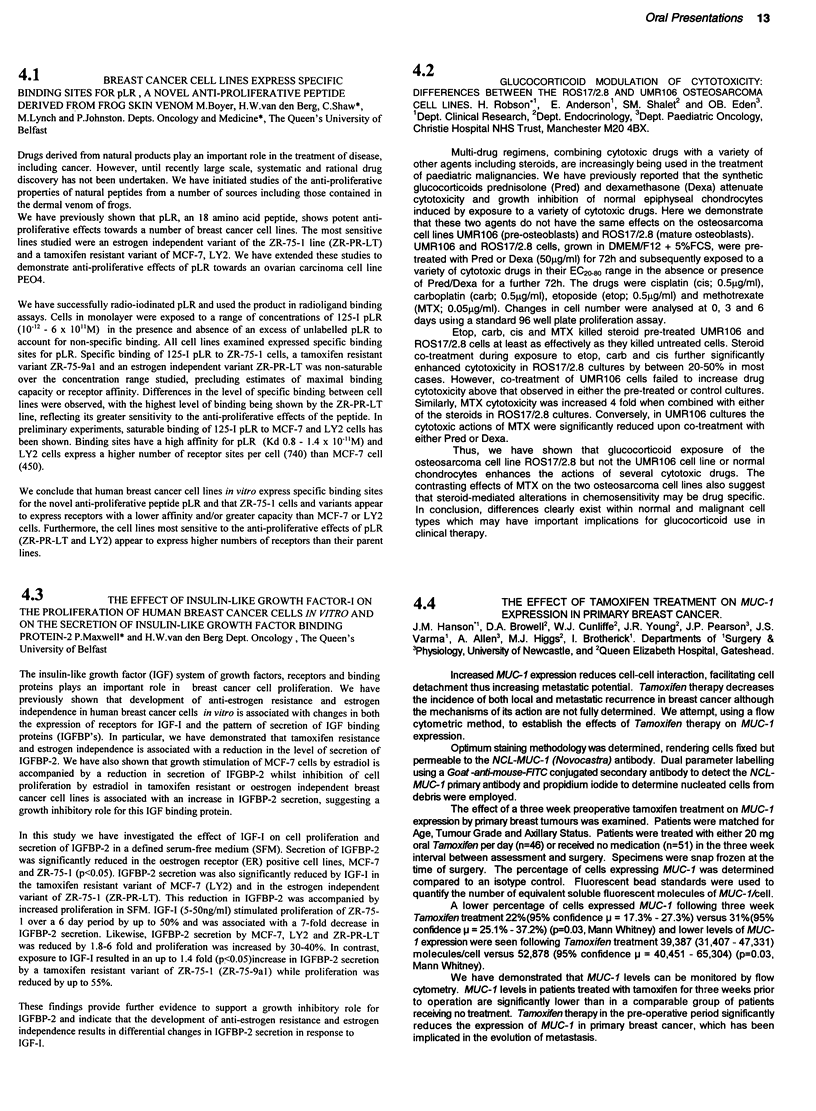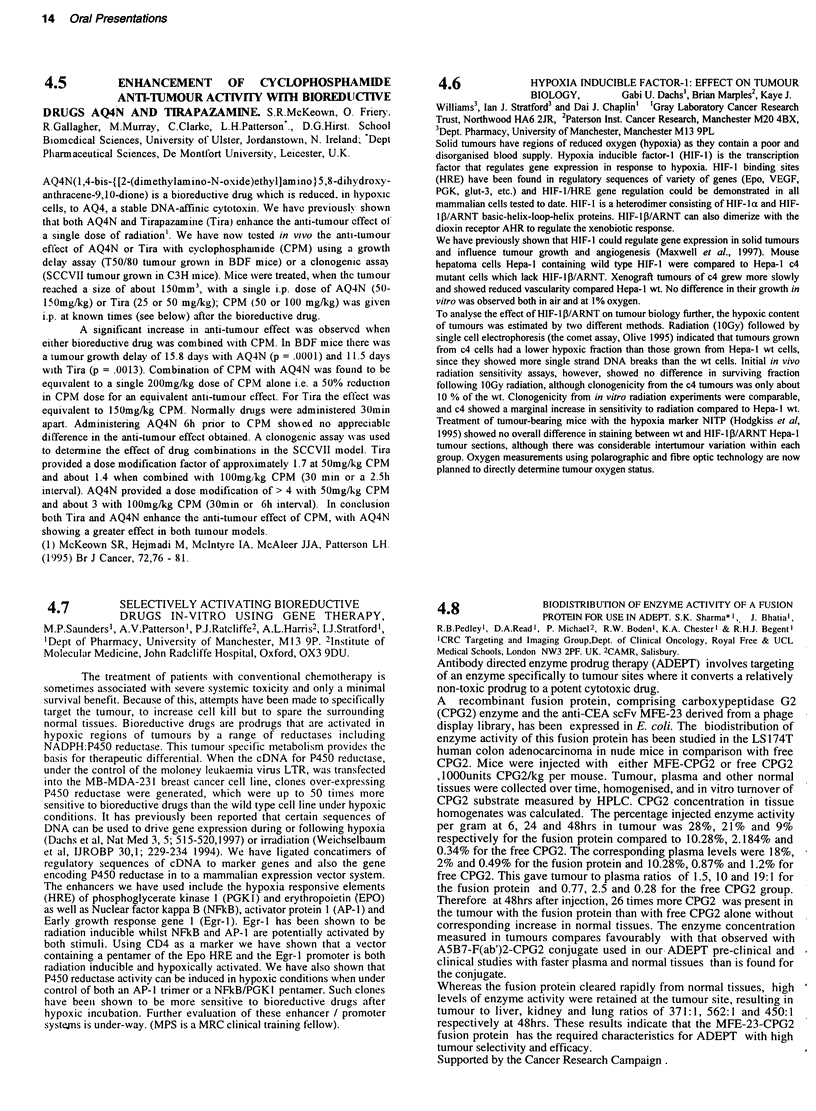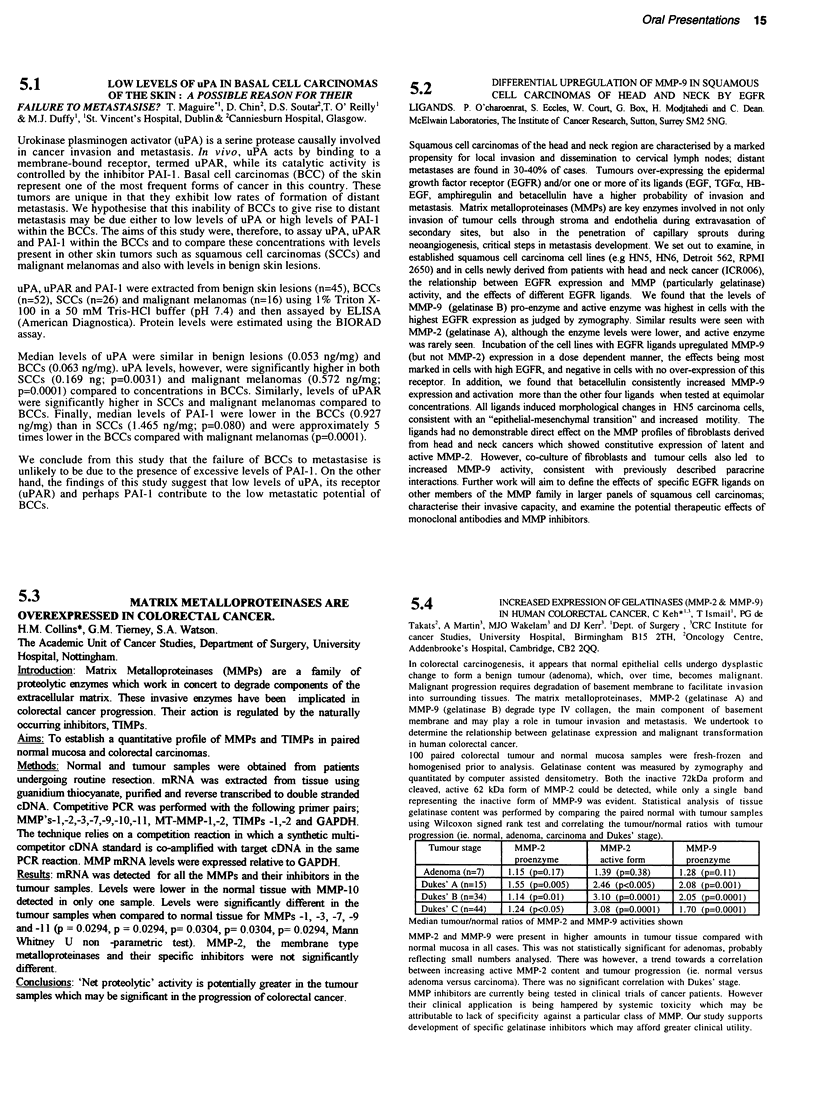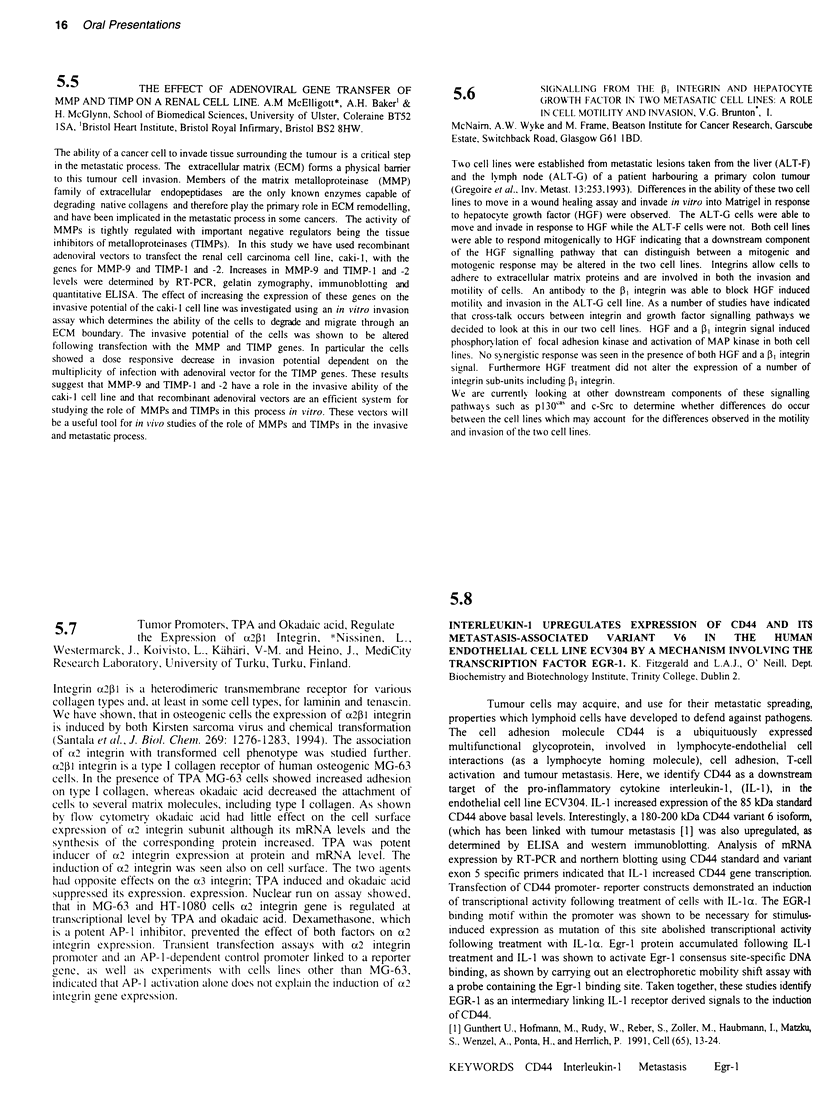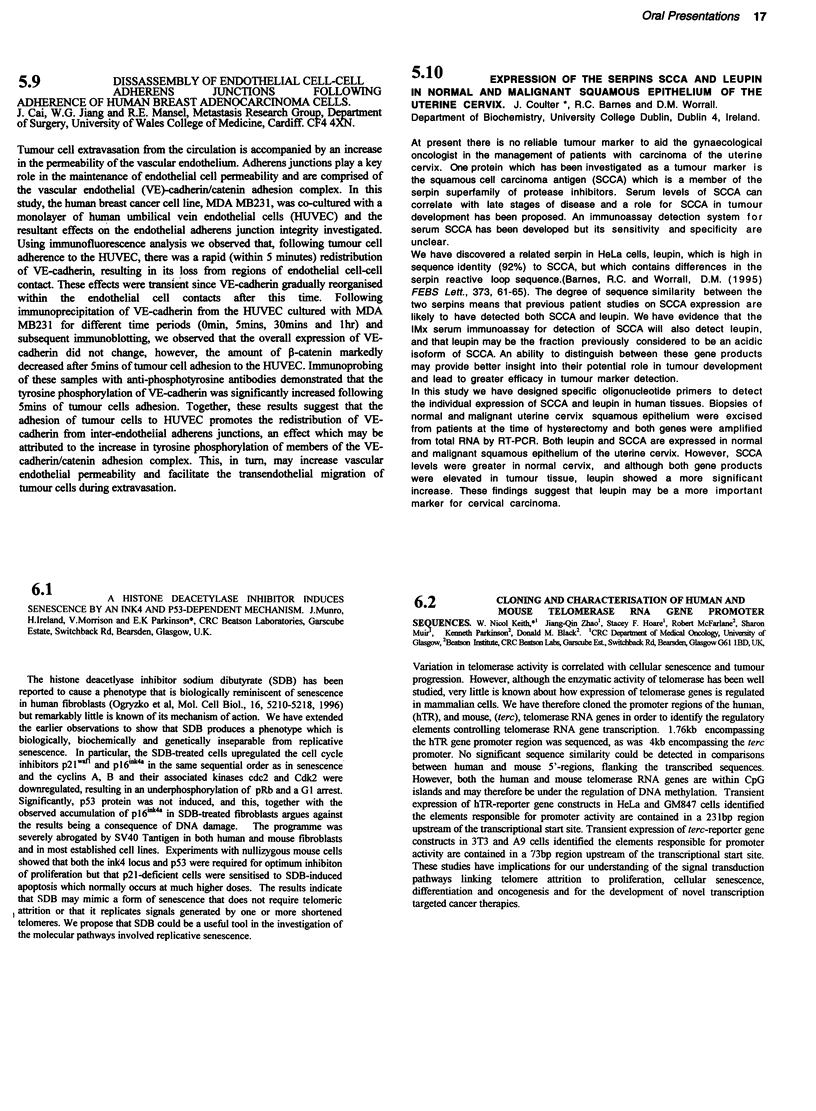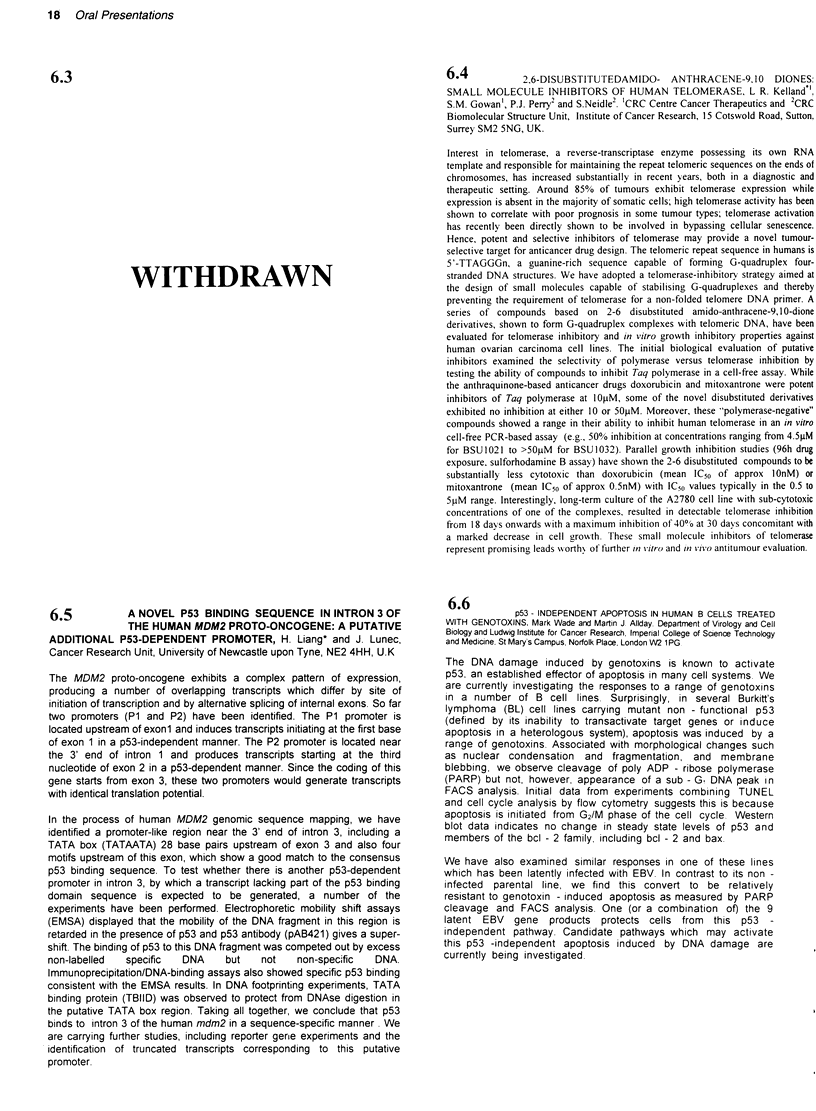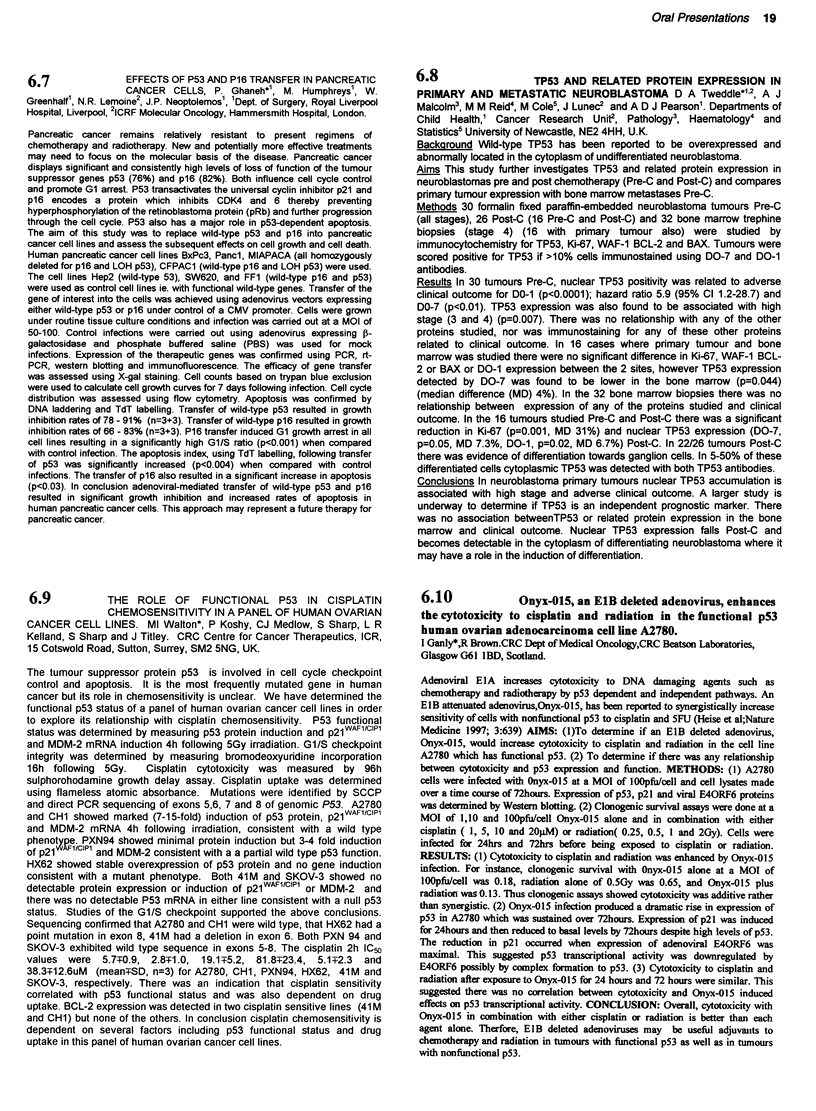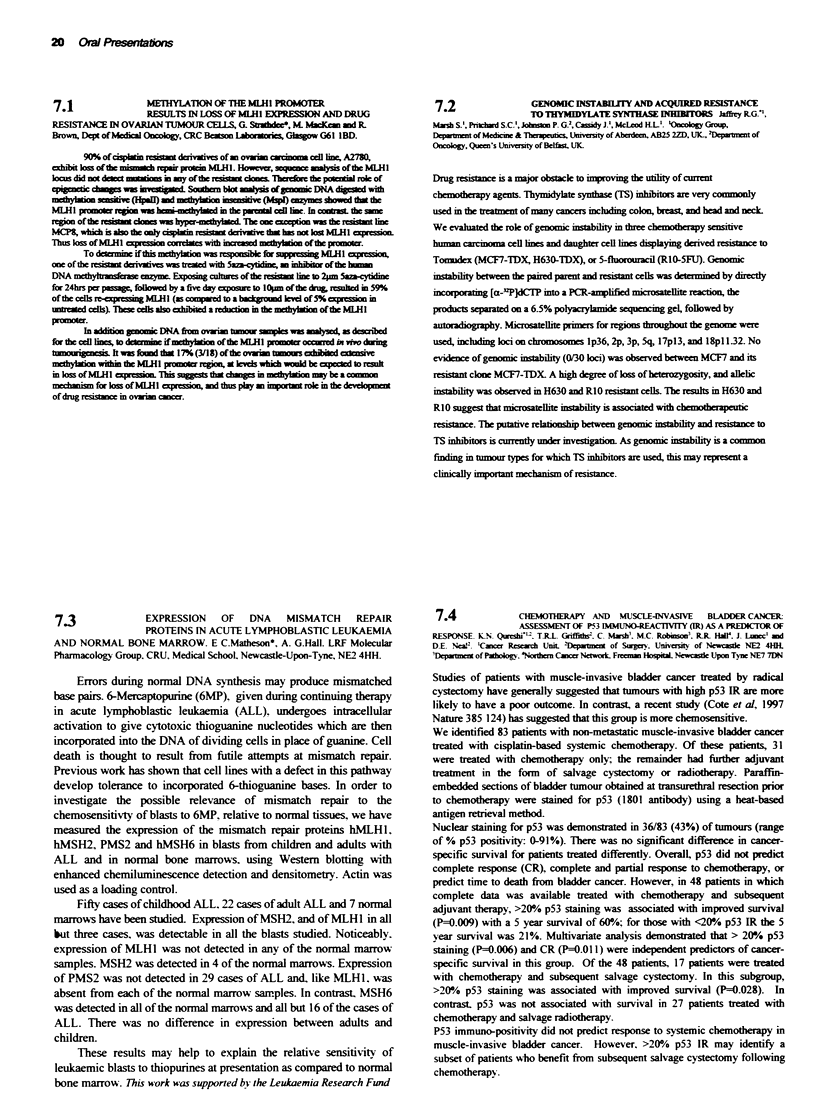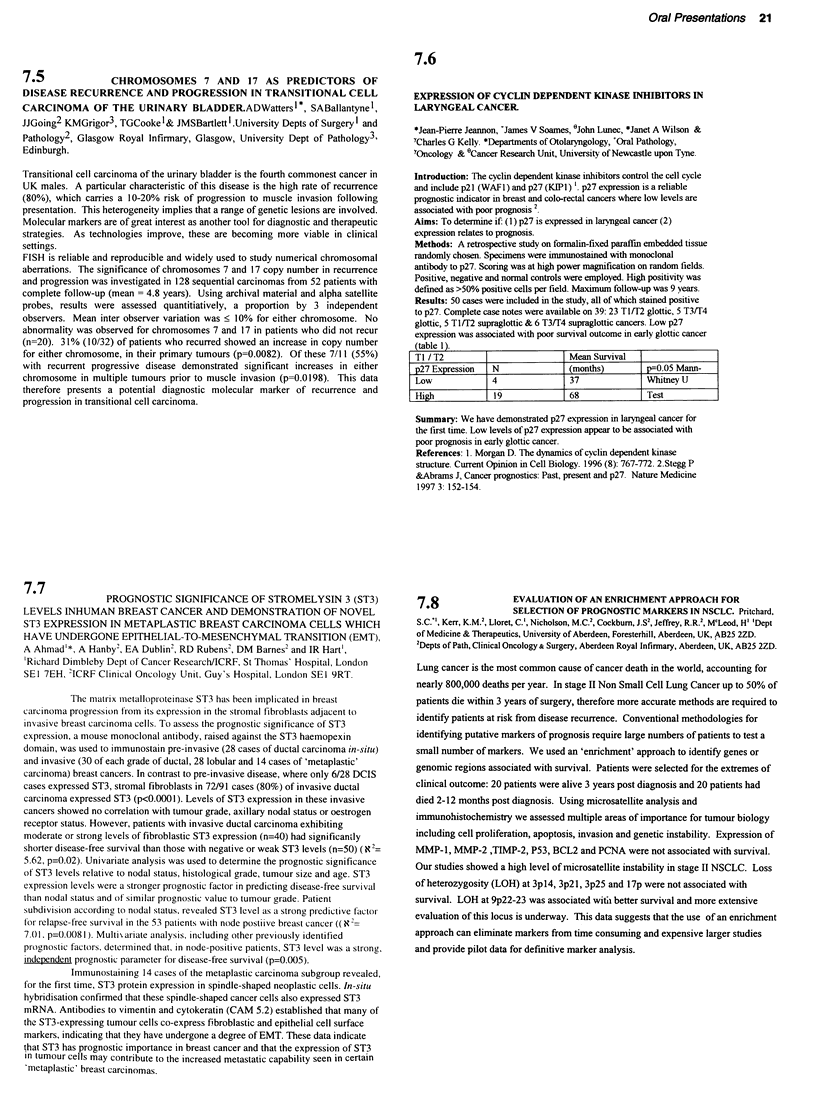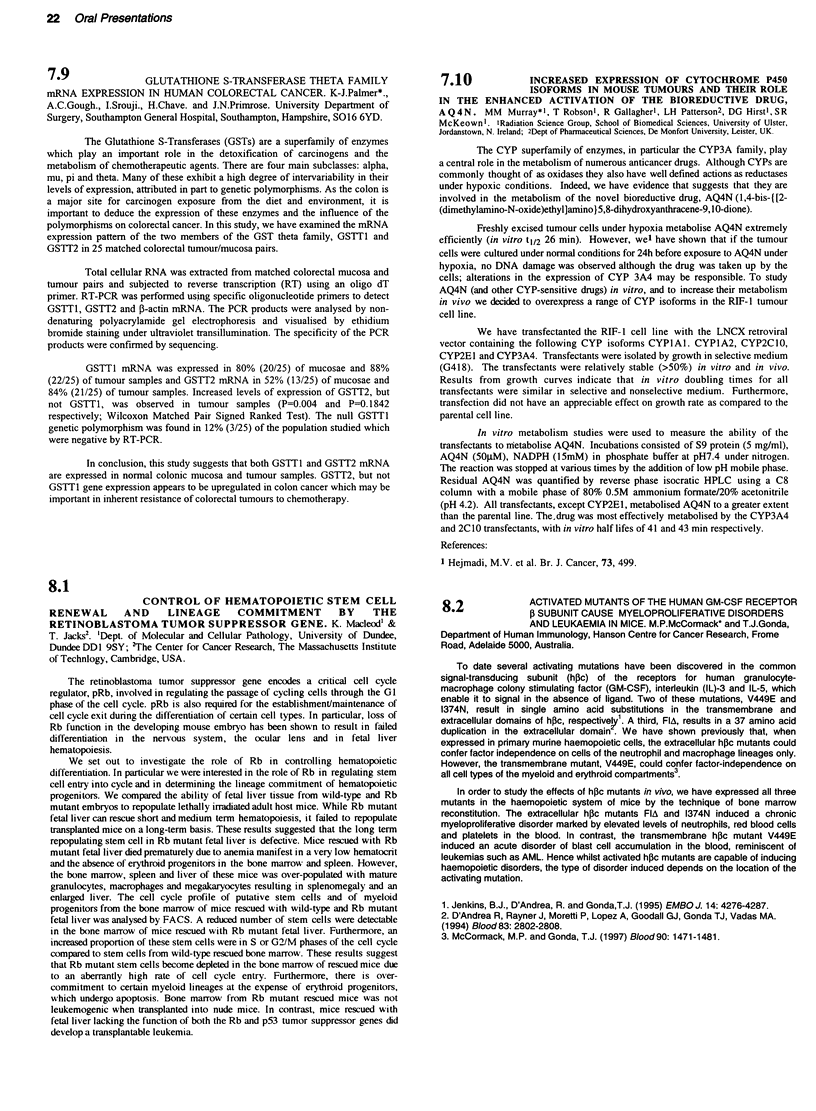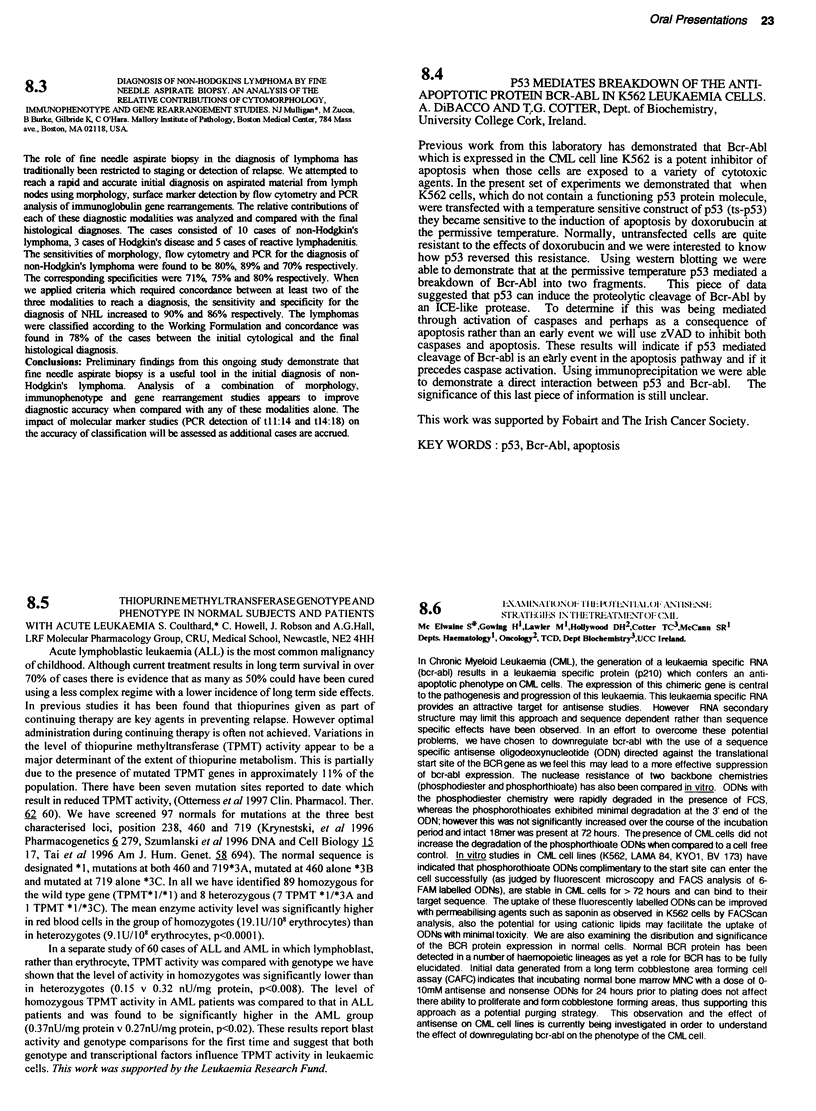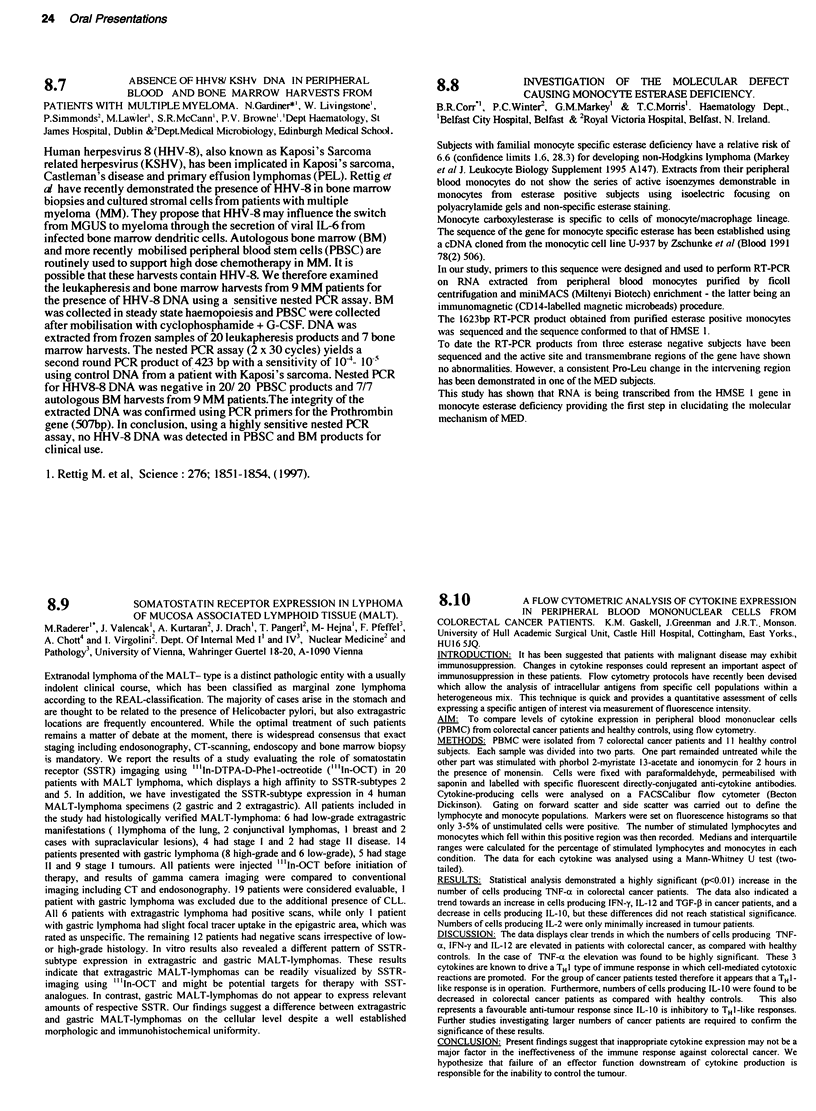# Oral Presentations

**Published:** 1998

**Authors:** 


© Cancer Research Campaign 1998

### 1.1

**BAX/BCL-2 HETERODIMERIZATION IN APOPTOSIS OF THE HEMATOPOIETIC CELL LINE 32DCL3(G) DETECTED BY LASER CONFOCAL MICROSCOPY,** R. Gradini*, I. Silvestri, P. Sale, L. Giuliani, M. Realacci, A. Gradilone, P. Gazzaniga, O. Gandini, C. Santangelo, G. Naso and A.M. Aglianò, Dpt. of Exp. Medicine and Pathology, "La Sapienza" University, Rome, Italy.

The modulation of transcriptional expression of *bax*, proapoptotic, and *bcl-2*, antiapoptotic genes, is an early event in the p53 mediated programmed cell death. Oltvai and coworkers related this phenomenon to Bax/Bcl-2 heterodimerization (1). Recently, Knudson et al. proposed the possibility that *bax* and *bcl-2* genes are able to regulate the apoptosis independently (2).

Our experimental model, a non-tumorigenic interleukine-3 (IL-3) dependent murine hematopoietic cell line 32D Cl3(G), which undergoes terminal differentiation into granulocytes when exposed to granulocyte colony stimulating factor (G-CSF), offers a convenient system to study the expression of genes involved in apoptosis. In a previous work, we showed that apoptosis induced by IL-3 deprivation from the culture medium is substained by p53, which in turn modulates the *bcl-2* and *bax* expression (3).

In the present work we studied the heterodimerization of Bax and Bcl-2 by Laser Confocal Microscopy. The IL-3 treated cells showed a low colocalization index of Bax and Bcl-2 (heterodimerization), as detected by immunofluorescence. The colocalization index was much higher in cells deprived of IL-3 after 5 hours. The colocalization index was very high also in the presence of G-CSF in the culture medium (after 16 hours).

Our study shows that Bax/Bcl-2 heterodimerization is an early event, related to IL-3 withdrawal from the culture medium, in the apoptotic pathway of 32D Cl3(G) cells.

1. Oltvai ZN, Milliman CL, Korsmeyer SJ. Cell 74: 609-611, 1993.
2. Knudson CM and Korsmeyer SJ. Nature Gewnetics 16:358-363, 1997.
3. Aglianò AM, Naso G, Silvestri I, Gradilone A, Vercillo R, Napolitano M, Gazzaniga p, Gandini O, Realacci M, Santangelo C, Saccani G, Manzari V, Frati L. Intern. J. Oncol. 11:1271-1277, 1997.

### 1.2

DIETHYMALEATE, A GLUTATHIONE DEPLETING AGENT, INDUCES APOPTOSIS IN ADENOCARCINOMA PROSTATE CANCER CELL LINES R. N.T. Coffey, R.W.G. Watson, E. Murphy, J.M.Fitzpatrick, Dept. of Surgery, Mater Misericordiae Hospital, Eccles St. Dublin 7.

*Prostate cancer* is the second most prevalent non-cutaneous cancer among men in Ireland representing 10.6% of all cancer. The adult male prostate is chronically dependent upon a continuous supply of androgen in order to maintain a steady state equilibrium of cell proliferation and prostatic cell death. Androgen ablation, widely used in prostate cancer treatment, induces *apoptosis* of the prostate cancer cells. A certain fraction of the prostate cancer cells are capable of surviving androgen withdrawal. These androgen independent cells pose a problem in terms of current antiproliferative therapeutic treatments. Clearly such cells are capable of avoiding or inhibiting the cellular mechanism that activates cell death.

*Gluthathione* (GSH) depletion is an early stage of apoptosis and chemical depletion of such an endogenous antioxidant is associated with the induction of apoptosis in human neutrophils. (Watson *et al.*, Surgery **120**: 150-158,1996]

AIMS: *Diethylmaleate* (DEM), a known glutathione depleting agent, was investigated in relation to its ability to induce apoptosis in both androgen sensitive (LNCaP) and insensitive (PC-3) human adenocarcinoma prostate cancer cell lines.

METHODS: LNCaP and PC-3 cells were treated with different concentrations of DEM (25-250 uM). Apoptosis was assessed four days post-treatment by propidium iodide DNA staining and confirmed by Annexin V and DNA gel electrophoresis. GSH depletion was measured by the DTNB colourimetric assay.

Table: **% Apoptosis (Propidium Iodide incorporation)** · **GSH depletion (nM/$10^6$ cells)**

| | CON. | DFM 25uM | DEM 100uM | DEM 250uM | ETOP. 120 uM | | CON. | DEM 250uM |
|---|---|---|---|---|---|---|---|---|
| LNCaP | 20.63 ± 5.8 | --- | 51.93 ± 10.54* | 80.3 ± 1.2* | 62.02 ± 4.4* | | 34.7 ± 1.7 | 12.5 ± 3.75* |
| PC-3 | 3.34 ± 1.4 | 5.05 ± 0.4 | 23.2 ± 1.5* | 37.25 ± 4.8* | 38.37 ± 2.6* | | 69.6 ± 2.5 | 27.45 ± 1.55* |

Results = Mean ± SD, * $p<0.05$ vs. CON. (Students t-test)

RESULTS: DEM which inhibited cellular proliferation was also shown to induce apoptosis in a dose dependent manner in both the androgen sensitive and insensitive cancer cell lines (Table). Associated with apoptotic induction was a depletion in the level of intracellular glutathione. The ability of glutathione depletion to induce apoptosis in androgen sensitive and insensitive cells may demonstrate a potential clinical role for this drug in the treatment of prostate cancer.

### 1.3

VITAMIN D ANALOGUES POTENTIATE TNFα AND CERAMIDE INDUCED APOPTOSIS IN HUMAN BREAST CANCER CELLS, G. Pirianov, S.Y. James and K.W. Colston, Dept. of Cellular and Molecular Sciences, St George's Hospital Medical School, London SW17 ORE

Previous studies have demonstrated that synthetic vitamin D analogues induce apoptosis in cultured breast cancer cells and cause regression of experimental rat mammary tumours. Mechanisms by which vitamin D analogues promote apoptosis could involve suppression of cell survival signals and/or induction of genes that stimulate apoptosis. In the present study we have examined whether vitamin D analogues may potentiate responsiveness of breast cancer cells to known mediators of apoptosis. TNFα is a potent cytokine which demonstrates anti-tumour activity both *in vivo* and *in vitro* and induces apoptosis in a variety of tumour cell lines. We have assessed the effects of two synthetic vitamin D analogues, EB1089 and CB1093, in addition to the native hormone, $1,25(OH)_2D_3$, on the cytotoxic action of TNFα on MCF-7, T47D and Hs578T human breast cancer cells. In MCF-7 cells, pretreatment with the vitamin D analogues (0.2 - 50nM) was found to substantially potentiate the cytotoxic effects of rhTNFα (0.2 - 20ng/ml) assessed by neutral red staining, with CB1093 being the most potent. MCF-7 cells pretreated for 65h with 20nM CB1093 showed 75% cytotoxicity with TNFα (20ng/ml for 22h) compared to 30% in control cultures. CB1093 also potentiated cytotoxic effects of TNFα in T47D cells. Hs578T cells were resistant to TNFα-induced apoptosis in the presence or absence of vitamin D compounds. Mechanisms involved in the potentiation of TNFα-induced apoptosis are unknown but vitamin D derivatives may promote recognition of the TNFα stimulus or promote a common apoptotic pathway. Generation of ceramide through hydrolysis of sphingomyelin appears to play a role in TNFα induced apoptosis. Since $1,25(OH)_2D_3$ has been reported to activate the sphingomyelin cycle in several leukaemic cell lines, we have assessed interactions between the vitamin D analogues and ceramide on breast cancer cell proliferation, cell viability and apoptosis (quantitated by MTS dye-reduction and DNA fragmentation assays). MCF-7, T47D and Hs578T breast cancer cells were pretreated with CB1093 (50nM for 48h) and exposed to $C_2$ ceramide (0.5 - 20μM for 24h). In control cultures, loss of cell viability was observed with $C_2$ concentrations >5μM. However, pretreatment with CB1093 increased responsiveness to $C_2$ such that significant cytotoxicity was detected with 0.5, 1.0 and 2.5μM $C_2$ in MCF-7, T47D and Hs578T cells respectively. CB1093 was also able to increase the level of ceramide-induced intranucleosomal DNA fragmentation in all breast cancer cells. These findings suggest that ceramide might be involved in a common pathway in TNFα and vitamin D-mediated apoptosis.

### 1.4

**CONTROL OF NORMAL AND MALIGNANT HUMAN UROTHELIAL CELL DEATH AND SURVIVAL BY THE TNF SUPERFAMILY.**

U. Bugajska, J. Southgate & L.K. Trejdosiewicz. ICRF Cancer Medicine Research Unit, St James's University Hospital, Leeds LS9 7TF, UK.

Members of the Tumour Necrosis Factor (TNF) receptor family are crucial in controlling lymphoid cell survival and elimination. However, the significance of their expression by normal *versus* malignantly-transformed epithelial cells is poorly understood.

We have studied a human uroepithelial cell system. Transitional cell carcinoma (TCC) cell lines (EJ, T24, 253J, RT112, VM-CUB-3, VM-CUB-1, COLO-232, KK47, RT4, HT1197) were used and compared with the normal human urothelial (NHU) cells (Southgate *et al*, *Lab Invest 1994*; **71**: 583). CD40 ligation was studied using soluble trimeric CD40L and by co-culture with CD40L-transfected fibroblasts (3T3CD40L) or control transfectants (3T3neo). The effects of TNFα and ligation of Fas (CD95, Apo-1) receptor were also investigated. Cell death was analysed by flow cytometry using Annexin V and propidium iodide and confirmed by the "JAM" test of DNA fragmentation.

All 14 NHU cell lines tested but only 4/10 TCC cell lines expressed CD40. The $CD40^+$ TCC cell lines underwent extensive apoptosis after co-culture with 3T3CD40L, but not 3T3neo. Soluble CD40L was growth inhibitory but did not induce apoptosis in TCC cells and ligation of CD40 had no effect on NHU cells. TNFα was growth inhibitory to NHU and some, but not all, TCC cells. NHU from some donors did not undergo TNFα-mediated apoptosis, whereas others were moderately susceptible. The well- and moderately-differentiated RT4 and RT112 TCC cell lines, respectively, underwent extensive apoptosis, increased even further by inhibition of protein synthesis with cyclohexamide (CHX). By contrast, anaplastic EJ cells were resistant to TNFα-mediated apoptosis, and only moderately sensitive with CHX. NHU cells were susceptible to Fas-mediated apoptosis, as were RT4 and RT112. EJ cells were completely resistant, unless in the presence of CHX.

Thus, these data demonstrate a differential sensitivity to CD40-ligation in normal *versus* tumour cells. Some carcinomas may escape immune-mediated destruction by either failure to express CD40 or by avoiding apoptosis following ligation of TNF/Fas receptors by an active mechanism involving protein synthesis.

### 1.5 EGFR BLOCKADE ON HN5 CELLS BY TK INHIBITOR OR ANTIBODY INDUCES TERMINAL DIFFERENTIATION AND APOPTOSIS.

H. Modjtahedi*[1], K. Affleck[2], C.Stubberfield[2] and C.Dean[1]. [1]The Institute of Cancer Research, Sutton.[2]Glaxo/Wellcome Medicines Research Centre, Stevenage, UK.

Human squamous cell carcinomas frequently overexpress the epidermal growth factor receptor (EGFR) and this is often associated with poor prognosis in patients with these cancers. The high level of expression of the EGFR provide an important target for therapy and we and others have shown that monoclonal antibodies (mAbs) which block the activation of the receptor by the EGF family of ligands inhibit the growth of EGFR overexpressing tumours *in vitro* and induce the regression of established tumours grown as xenografts in athymic mice (e.g. Modjtahedi H, Komurasaki T, Toyoda H and Dean C (1998) Int. J. Cancer 75, 310: Modjtahedi H, Eccles S, Sandle J, Box G, Titley J, and Dean C (1994) Cancer Research 54, 1695). Inhibitors of the tyrosine kinase associated with the EGFR have also been shown to block receptor activation and prevent tumour cell proliferation. Using the EGFR-overexpressing head and neck carcinoma cell line HN5, we have compared the biological consequences of treatment with an inhibitor of EGFR tyrosine kinase (PD153035) with anti-EGFR monoclonal antibodies (mAbs) ICR63 or ICR80. We found that both the anti-EGFR mAbs and the TK-inhibitor produce similar biological changes namely, they inhibit the EGF and TGF $\alpha$-induced tyrosine phosphorylation of the receptor and the growth in culture of HN5 cells. At concentrations above 100nM, the TK inhibitor prevented the growth in culture of HN5 cells completely with an IC50 of 40nM. With the anti-EGFR mAbs, growth of HN5 cells was inhibited completely at concentrations above 4nM. More importantly, we found that like the anti-EGFR mAbs treatment with the TK-inhibitor directs HN5 cells to undergo terminal differentiation as monitored by the expression of cytokeratin 10. Furthermore, this was accompanied by apoptosis as determined by 7-Amino Actinomycin D staining (7-AAD). We conclude that EGFR blockade by anti-EGFR mAbs or TK-inhibitor influences the growth in culture of EGFR overexpressing tumours by directing terminal differentiation and inducing apoptosis. It remains to be seen whether the combined treatment with anti-EGFR mAbs and TK inhibitors offer therapeutic advantage over individual treatment or the use of conventional therapies.

### 1.6 REGULATION OF TUMOR CELL SURVIVAL BY THE IGF-I RECEPTOR C-TERMINUS.

Rosemary O'Connor*[1], Mariana Resnicoff[2] Renato Baserga[2] Eileen Dalton[1], Maureen McDonagh[1] Department of Biochemistry, University College, Cork, Ireland[1] and Kimmel Cancer Center, Thomas Jefferson University, Philadelphia, USA

The insulin-like growth factor I receptor (IGF-IR) activated by its ligands IGF-I or IGF-II, mediates suppression of apoptosis, which is critical for tumourigenesis and for the maintenance of tumour cells. There is a very strong association between the levels of circulating IGF-I/IGF-II and IGF-IR expression with tumors of the lung, breast, and prostate. Circulating IGF-II levels were recently shown to be a better indicator of prostate cancer than prostate specific antigen. Therefore, the IGF-IR is a very rational target for pharmacological agents that would inhibit the survival of tumor cells.

The C-terminus of the IGF-IR has a negative impact on the ability of the receptor to suppress apoptosis, and seems to constitute a regulatory domain for the receptor (O'Connor et al., Mol. Cell Biol. 17: 427-435, 1997) A critical role for the C-terminus in regulating IGF-IR-mediated tumor cell survival was confirmed by studying this domain expressed as protein derivatives in tumor cells. The membrane-targeted C-terminal derivative, MyCF, was found to induce apoptosis when transiently transfected into MCF-7 breast carcinoma cells, and it sensitized the cells to U.V irradiation. This could be reversed by specific point mutations, suggesting that the pro-apoptotic effect was due to a dominant-negative effect caused by sequestering molecules that normally interact with the C-terminus of endogenous IGF-IR (Liu et al., Cancer Res. 58: 570-576, 1998).

In order to further study the activity of the C-terminal derivatives they were stably transfected into MCF-7 cells and ovarian carcinoma cells CaOV3. MyCF inhibited tumorigenesis in nude mice and induced apoptosis in vivo. This could be blocked by over-expression of Bcl-2. MyCF did not alter IGF-IR-mediated activation of P-I3 kinase and AKT kinase, both of which are implicated in suppression of apoptosis by the IGF-I receptor. This suggests that the C-terminal derivatives induce apoptosis by blocking a pathway distinct from the PI3 kinase/AKT pathway, or alternatively by activating a pro-apoptotic pathway.

Determining how the C-terminal derivatives suppress IGF-IR-mediated cell survival should lead to possibilities for blocking this pathway in tumors, and also elucidate the regulatory mechanisms of the IGF-I receptor.

### 1.7 A LOW-RISK DIET MAY PROTECT AGAINST COLON CANCER BY UP-REGULATING APOPTOSIS

Richard J. Hambly*[1], Morag Cunninghame[1], Philip Brown[1], Philip J. Rijken[2] and Ian R. Rowland[1]. [1]BIBRA International, Carshalton, Surrey, SM5 4DS, UK; [2]Unilever Research Laboratorium, Vlaardingen, The Netherlands.

The aim of this research was to evaluate whether diet played a role in the regulation of apoptosis, since the induction of programmed cell death may represent a possible mechanism of protection against cancer of the colon

30 Sprague-Dawley rats were randomly allocated to receive diets containing either high-risk (HR) or low-risk (LR) factors for colon cancer for 3 weeks (Hambly, R.J. *et al.* 1997. *Nutr. Cancer* **27**: 250). One day before post mortem, 1,2-dimethylhydrazine (DMH) in saline was administered orally at 0, 5, 10, 20 and 25 mg $kg^{-1}$ body weight to 3 animals per group. At post mortem the colon was removed, formalin fixed, and 1cm regions of the proximal, mid and distal colon were embedded in paraffin wax. Sections (5µm) were rehydrated, protein digested by a 15 minute digestion with 25µg $ml^{-1}$ proteinase K at 20°C, and endogenous peroxidase removed by treatment with 2% $H_2O_2$ for 5 minutes at 20°C. An ApopTag plus kit (Appligene-Oncor, France) was used to stain apoptotic bodies in crypts. Adjacent sections were stained with haematoxylin and eosin (H&E) to confirm the incidence of apoptotic cells by conventional histology. The number of whole crypts per slide and the numbers of apoptotic bodies in transverse sections of whole crypts were counted. Data was analysed after $log_{10}$ transformation using an ANOVA model based on a factorial design with repeated measures.

Oral dosing with the carcinogen DMH induced a significant dose-dependent increase ($p<0.001$) in apoptosis in the colon. Carcinogen-induced apoptosis was significantly increased in the colonic crypts of animals receiving a LR diet compared with those receiving a HR diet ($p<0.001$). Analysis of the colon by region showed significantly less apoptosis occurred in the proximal colon compared with the distal or mid colon regions ($p<0.001$). There was also a strong trend ($p=0.053$) to indicate that the rate of apoptosis in the mid region was greater than in the distal region of the colon. Statistical analysis highlighted two other significant effects: Firstly, there was a significant region by diet interaction ($p<0.05$), indicating that the low-risk diet increased the rate of apoptosis by a greater amount in the distal and mid regions of the colon than the proximal colon. Secondly, for both diets, there was a significant ($p<0.001$) region by dose interaction, indicating that the higher the dose of DMH the greater the difference between the proximal region, which had the lowest rate of apoptosis, and the other two regions.

Our results suggest that diet-induced up-regulation of apoptosis represents a mechanism of protection against the early stages of carcinogenesis in the colon.

### 1.8 MYCOBACTERIAL DNA INDUCES APOPTOSIS IN TUMOUR CELLS,

M.C. Filion*[1], R. O'Shea[2], J.K. Collins[2] and N.C. Phillips[1], [1]Faculté de Pharmacie, Université de Montréal, Qc., Canada, H3C 3J7, [2]Dept. of Microbiology, University College Cork, Ireland.

Live mycobacteria or mycobacterial preparations possess potent anti-tumour activity. This anti-tumoural activity has been ascribed to cell wall components or to immunostimulatory proteins. A mycobacterial cell wall complex (MCC) derived from the non-pathogenic *Mycobacterium phlei* (*M. phlei*) has been shown to have anti-tumour activity. In analysing the composition of MCC we found that it contains approximately 5% DNA in the form of short oligonucleotides (approximately 100 base-pairs). We have therefore evaluated whether DNA extracted from MCC or from *M. phlei* directly induces apoptosis in tumour cells. The induction of apoptosis was determined by a) inhibition of tumour cell proliferation, b) nucleosome-sized DNA fragmentation, c) the release of soluble nuclear mitotic apparatus protein (NuMA) and d) the presence of hypodiploid peaks in flow cytometric analysis of cellular DNA.

We have found that DNA extracted from either MCC or from *M. phlei* directly induces apoptosis (incubation time 48-72 hours) in a wide range of human and murine tumour cell lines in the absence of immune effector cells. The tumour cells tested were: human monocytic THP-1 leukemia, human pro-monocytic HL-60 leukemia, murine monocytic RAW 264.7 leukemia, human Jurkat T lymphoblast, murine B-16 melanoma, human colon SW260 adenocarcinoma, human oesophageal squamous OC2 carcinoma, human transitional bladder HT-1197 and HT-1376 carcinoma. The induction of apoptosis appears to be independent of tumour cell type and of escape mechanisms such as Fas-ligand and drug resistance, since all cells tested enter apoptosis after contact with *M. phlei*-derived DNA. DNAse treatment of MCC, DNA extracted from MCC or from *M. phlei* significantly inhibits the induction of apoptosis. We further demonstrate that the induction of apoptosis does not appear to be related *per se* to the methylation state of putative CpG motifs or to the molecular weight of the DNA.

The induction of apoptosis by mycobacterial DNA may help to explain the therapeutic activity of a number of mycobacterial preparations in the treatment of cancer.

Acknowledgement. We wish to thank Bioniche Inc., London, Ontario, Canada, for kind gift of MCC used in this study.

### 2.1

**TOPOISOMERASE II IS A TARGET FOR ANTHRACYCLINE-MEDIATED ACTIVATION OF NFκB**

**Marion P. Boland*, Gillian J. Callan and Luke A.J. O'Neill. Department of Biochemistry, Trinity College, Dublin 2.**

The anthracycline antibiotics, daunorubicin and mitoxantrone, can activate the transcription factor, NFκB, and induce κB-dependent gene expression in HL60 cells. Reactive oxygen species generated by endogenous cellular processes have been implicated in this activation, and not ceramide, although its levels are raised in cells in response to these drugs [1]. In continuing studies, we have investigated the role of topoisomerase II (topo II) enzymes, a major target for these drugs, in mediating mitoxantrone-induced NFκB activation. Topo II enzymes modulate DNA topology by introducing transient double-strand breaks into the DNA backbone, allowing passage of an intact helix through the broken DNA. Topo II-targeted drugs stabilize this reaction intermediate giving rise to protein associated strand breaks. Mammalian cells possess an alpha and a beta isoform, both of which may be important targets for anthracyclines. We have found that a reduction in the level of the beta isoform of topo II profoundly effects the ability of mitoxantrone to induce both NFκB activation and apoptosis in non-mdr HL60-MX cells, compared with the the parental cell line (HL60-P), where a dose-responsive activation was observed. The absence of the beta isoform was confirmed by immunoblot analysis. Significantly, both cell lines were equally response to apoptotic induction and NFκB activation by agonists which do not act via topo II enzymes, suggesting that pathways leading to these events are intact in these cells. In further studies, Adr-1 cells (non-mdr phenotype) displaying a 3-4 fold hypersensitivity to mitoxantrone, were responsive to NFκB activation whereas the parental cell line (CHO) was only weakly responsive at the highest doses employed. Apoptotic induction paralleled these observations, and topo II alpha levels were confirmed to be elevated in Adr-1 cells. The cellular level of topoisomerases may dictate the therapeutic efficacy of anthracyclines drugs. These enzymes may also be novel candidates which regulate the activation of the transcription factor, NFκB, in response to such agents which damage DNA. As NFκB activation may be involved in apoptosis, this effect may be an important aspect of the cellular responses to the anthracyclines.

[1] Boland, M.P., Foster, S.J. and O'Neill, L.A. (1997) J.Biol.Chem., 12952-12960.

anthracyclines NFκB topoisomerase II apoptosis

### 2.2

**Validation of pharmacologically-guided dosing of carboplatin in paediatric patients.** Thomas HD, Boddy AV, Pearson ADJ, Pinkerton CR, Lewis I, Stevens M, Imeson J, Hobson R, and Newell DR. CRU, University of Newcastle upon Tyne, for New Agents Group, UKCCSG.

Pharmacologically-guided dosing of carboplatin based on renal function (RF) is common practice in adults (Calvert JCO, 7:1748 (1986)). A similar approach has been proposed to optimise dosing of carboplatin in paediatric patients (Newell JCO, 11: 2314 (1993)). This study investigated the accuracy and precision of this method of dosing compared to conventional dosing based on surface-area (SA).

Thirty nine patients, aged 0.3-16.2 (median 3.4) years, were randomised to receive carboplatin dosed to a target AUC using either RF or SA, crossing over to the alternative dosing method on the next course. GFR ranged from 27-114ml/min/m$^2$. The target AUC was defined as 1.325mg/ml.min per 100mg/m$^2$ of carboplatin based on the median clearance. The interval between courses varied from 20 to 83 days.

Dosing using either RF or SA resulted in an AUC that, on average, was not different to the target. There was no difference between the methods in the average AUC as a % of target. However, the variation about the target AUC was less for RF (standard deviation (SD)=17%, interquartile range (IQR)=25%) than for SA (SD=25%, IQR=36%) (p=0.02). Also, the proportion of courses falling within 20% of the target was larger for RF than for SA (74 vs 49%, p=0.05). By direct comparison, AUC using SA dosing differed from target by 22%, compared with 13% for RF based dosing (p=0.001).

Thus, in this prospective, randomised cross-over study, RF based dosing results in more reproducible and reliable AUC than dosing based on SA. The proportion of courses outside an acceptable range with SA based dosing suggests that this is of clinical significance.

### 2.3

**A PHASE ONE PHARMACOKINETIC STUDY OF CGS27023A, A MATRIX METALLOPROTEINASE INHIBITOR.** N.C. Levitt1*, F.Eskens2, D.J.Propper1, A.L.Harris1, L.Denis2, T.S.Ganesan1, R.A.Mather3, L.McKinley1, A.Planting2, D.C.Talbot1, V.Van Beurden2, M.Van der Burg2, S. Wilner1 and J. Verweij2. 1: ICRF Unit, Churchill Hospital, Oxford OX3 7AR, U.K. 2: Rotterdam Cancer Institute and University Hospital, Rotterdam, Netherlands. 3: Novartis, Switzerland.

CGS27023A is an orally administered, broad spectrum matrix metalloproteinase inhibitor with antitumour activity in various animal models. In this phase one study 36 patients, (median age 56 years, range 32 to 74, 12 female, 24 male, median PS = 0), with advanced solid tumours, ( colorectal 10, mesothelioma 6, melanoma 4, other 16) have been entered to date, at five dose levels (150mg bd - 600mg tds) for a total of 340 patient weeks (median 7 weeks, range <1 to 45 per patient). Two main toxicities were observed. Five patients developed a widespread, self limiting maculopapular rash after 1 week of treatment, 3 of whom discontinued therapy. Rash was not observed at doses ≤ 300mg bd. Nine patients developed mild to moderate myalgia/arthralgia, one of whom stopped treatment. One patient also developed palmar nodules, which resolved after stopping the drug. Six patients had mild nausea (CTC ≤ grade 1). Musculoskeletal events and nausea were not dose related. There was no myelotoxicity. Dose limiting toxicity was not observed. Pharmacokinetics showed CGS27023A was rapidly absorbed and eliminated with minimal accumulation after 28 days continuous dosing. Plasma concentrations >10 x IC 50 for the target enzymes (including collagenase-MMP1, gelatinase-MMP9 and stromelysin 1-MMP3) were observed at all dose levels, with Cmax within 1 hour of dosing. Plasma levels ≥ 5 x IC 50 lasted for 10.6 and 20.0 hours, per 24 hours, at 300mg bd and 600mg tds, respectively. Seven patients had stable disease and remained on treatment for ≥ 12 weeks (2 each on 150mg bd, 300mg bd, and 600mg bd and 1 on 400mg tds). There were no tumour regressions. In conclusion CGS27023A appears well tolerated at a doses ≤ 300mg bd with biologically relevant plasma levels. At higher doses the only major toxicity was rash, observed in 5/22 patients.

### 2.4

**PHASE I/II DOSE ESCALATING STUDY OF GEMCITABINE AND PACLITAXEL IN NON-SMALL CELL LUNG CANCER (NSCLC): PRELIMINARY REPORT.**

KJ O'Byrne*[1], S Beare[1], F Shields[1], R Sharma[1], K Jeyapalan[2], WP Steward[1], [1]University Department of Oncology, Leicester Royal Infirmary, and [2]Department of Radiology, Glenfield General Hospital, Leicester, UK.

Gemcitabine (Gem) and paclitaxel (Taxol) each have response rates in the region of 20% in the treatment of NSCLC. Either agent combined with a platinum gives response and 1 year survival rates of 40 - 50%. Low dose weekly Taxol, infused over 1 hour, has equivalent anti-tumour activity to standard Taxol in relapsed ovarian cancer but reduced toxicity. In a phase I/II study we evaluated escalating doses of Gem and Taxol given weekly on days 1, 8 and 15 of 28 day cycles in chemotherapy naïve NSCLC patients (pts). Taxol was given over 1 hour followed by Gem over 30 minutes. Two cohorts of 3 pts, 5 men and 1 woman: age range 54 - 76 years, were treated. Cohort 1 - Taxol 40 mg/m$^2$ & Gem 800 mg/m$^2$; cohort 2 - Taxol 40 mg/m$^2$ & Gem 1,000 mg/m$^2$. Dexamethasone 8 mg, chlorpheniramine 10 mg and ranitidine 50 mg were given intravenously as premedication 30 minutes before chemotherapy. Dexamethasone 8 mg bid po was continued for 24 hours with metaclopramide 10 - 20 mg tid po prn for nausea. Pneumonitis was seen after 4 cycles in 1 pt in cohort 1 and after 2 cycles in 1 pt in cohort 2. Investigations, including bronchial washings in 1 and a lung biopsy in the other, showed no evidence of an infective aetiology. The pneumonitis responded to high dose prednisolone. Pulmonary infiltrates also developed in the 2 other pts in cohort 1. An empyema was diagnosed in 1 case and progressive disease in the other. Treatment was discontinued in the remaining 2 pts in cohort 2 after 2 cycles due progressive disease in 1 and an uncontrolled, tumour related, recurrent pleural effusion in the other. The combination was otherwise well tolerated without significant alopecia, nausea or vomiting, neuropathy, arthralgia or haematological complications. Three pts had partial responses to therapy. Pneumonitis, a recognised complication of Gem and also reported for Taxol, appears increased by using the agents in the schedule described. Given the encouraging anti-tumour activity and absence of other side-effects we will focus on understanding the pathogenesis of, and on preventing the pneumonitis induced by weekly Gem and Taxol.

### 2.5 TREATMENT OF ADVANCED REFRACTORY BREAST CANCER WITH DOCETAXEL ALTERNATING WITH EPIRUBICIN AND CYCLOPHOSPHAMIDE (EC) + G-CSF.

G.Weinländer, G.V.Kornek, K.Haider, M.Raderer, J.Valencak, W.Kwasny, E.Bareck, D.Depisch, E.Kovats and W.Scheithauer. Dept.of Int.Med.I, University.of Vienna, and Depts.of Surgery, Wiener Neustadt and Baden General Hospitals, Austria.

Fourty consecutive patients with advanced breast cancer failing palliative 1st-line chemotherapy (without anthracyclines) were entered in this trial. 34 of them are currently evalualuble for response and toxicity assessment. Fourteen were pre-, 19 were postmenopausal, and 1 was male. Their median age was 53 (41-72) years, and the median WHO performance status 1 (0-2). Predominant tumor sites were visceral in 29, and bone in 3 and soft-tissue in 2 patients. Prior 1st-line chemotherapy consisted of CMF (n=9), vinorelbine/5-FU/leucovorin (n=13) or vinorelbine/gemcitabine (n=12). Salvage treatment consisted of 3-weekly courses of docetaxel 100 mg/m2 iv alternating with epirubicin 100 mg/m2 + cyclophosphamide 800 mg/m2 + G-CSF 5mcg/kg/day given subcutaneously for 5 days following chemotherapy. After a median of 6 (2-6) treatment courses, 19/34 (56%) evaluable patients had objective tumor responses, including 5 CRs (15%); 8 additional patients (24%) had stable disease, and 7 progessive disease. The median time to response was only 2.0 (1-5) months, the median duration of both response and survival have not been reached yet. Dose-limiting hematologic side effects (WHO grade 4) occurred in 10 patients, and were complicated by septicemia in 3. Severe nonhematologic side effects (WHO 3) were noted in 3 patients and consisted of stomatits in 2 and neurotoxicity in 1. Alopecia occurred in all patients and was complete in 20 (59%). Other frequently encountered, though generally mild adverse reactions were nausea/vomiting (47%) and diarrhea (24%). We conclude that docetaxel alternating with EC/G-CSF is a very active regimen for the treatment of advanced breast cancer refractory to (non-anthracycline containing) 1st-line chemotherapy regimens.

### 2.6 POTENTIAL FOR DOSE ESCALATION OF CHART IN NON NON SMALL CELL LUNG CANCER(NSCLC): COMPARISON OF TWO STRATEGIES FOR LOCO-REGIONAL CONTROL.

C. McGibney*[1],O. Holmberg[2], B McClean[2], C. Williams[3], P.Sutton[1], J. Armstrong[1]. Departs. of Radiation Oncology[1], Physics[2], Radiology[3], St Luke's Hospital, Dublin.

Purpose: To compare the dose escalation possible for continuous, hyperfractionated, accelerated radiotherapy (CHART) for NSCLC using two alternative treatment strategies for loco-regional control: (1)Clinical strategy of elimination of elective nodal irradiation (ENI) alone or (2) Utilization of 3-D, conformal radiation therapy(3DCRT) also with the elimination elective nodal irradiation (i.e. 3D-ENI plans).

Methods: Standard CHART therapy plans,which include ENI, were generated using conventional 2D technology for 18 patients with NSCLC, stages Ib-IIIb. Further 2D plans were generated but with the elimination of ENI from the treatment portals thus forming 2D-ENI plans. Both sets were reconstructed in three dimensions using the patients' planning CT studies and subsequently compared with alternative 3D-ENI plans, generated de novo.

Baseline dose delivery to the primary tumour and to organs at risk was compared between the reconstructed 2D CHART, 2D-ENI and the 3D-ENI plans. The potential for escalation of dose beyond the 54Gy,the standard dose of CHART, using the 2D-ENI and the 3D-ENI plans, was also assessed.

Results: Target coverage was significantly reduced in the 2D CHART and the 2D-ENI plans when compared to the 3D-ENI plans, p=0.0001 and p=0.003, respectively. The target volume receiving the recommended (ICRU-50) 95% of the dose prescribed (V95) was reduced in the 2D CHART and 2D-ENI plans by 14% and 17%, respectively , p=0.0001. Maximum point dose to the spinal cord was reduced to an average of 19Gy in the 3D-ENI plans from an average of 40Gy and 39.7 Gy in the 2D CHART and 2D-ENI plans,respectively, p=0.0001 and p=0.0005. The mean and median dose to the oesophagus and heart were also significantly reduced in the 3D-ENI plans.

Dose escalation ,with the recommended target coverage retained, was possible in 10/18 of patients using the 3D-ENI strategy but in only 2/18 patients using a strategy of elimination of ENI alone. Dose escalation varied from 4% to 135% with a median escalation of 22% above the standard dose of CHART. The degree of escalation was significantly influenced by the tumour site & N stage, ANCOVA,p=0.008

Conclusions: 3DCRT is essential for optimum target coverage and facilitates dose escalation in more than half the patients with NSCLC. A clinical policy of elimination of elective nodal irradiation alone is not sufficient for either target coverage or for dose escalation.

### 2.7 RADIOIMMUNOGUIDED SURGERY (RIGS) OF COLORECTAL CANCER WITH SINGLE CHAIN Fv ANTIBODY TO CEA,

G. M. Boxer*, A. Mayer, D. O'Malley, E. Tsiompanou, M. C. Winslet, A. A. M. Lewis, A. P. Dhillon, A. J. W. Hilson and R. H. J. Begent. CRC Targeting & Imaging Group, Dept. of Clinical. Oncology, Royal Free and UCL Medical Schools, London NW3 2PF.

In RIGS radiolabelled anti-tumour antibody is given intravenously to patients before operation to resect cancer. Peroperatively the surgeon uses a hand-held gamma-detecting probe to locate tumour deposits in which radiolabelled antibody has localised. The probe is extremely sensitive and makes it possible to locate tumour deposits which are not detectable by the conventional means of inspection or palpation. Conventionally RIGS has been performed using a whole IgG antibody but these clear slowly from the bloodstream (β half life 24-72 hrs) so that it is usually neccessary to wait 4 weeks or more after injection before surgery is performed which is too long for many clinical situations. We have developed a genetically-engineered single chain Fv antibody (MFE-23) directed against carcinoembryonic antigen (CEA) with a much lower molecular weight than IgG (27 v 150 kD). Clearance from the circulation is more rapid than IgG and gives the prospect of performing surgery at much earlier times, with better discrimination between tumour and normal tissues. We have produced MFE-23 in a form suitable for clinical use, labelled it with $^{125}$iodine (I) and conducted a RIGS trial. 1 mg of $^{125}$I-antibody (90-285 MBq) was given, 24, 48, 72 or 96 hours before operation to 20 patients having surgery for primary or recurrent colorectal cancer. At surgery counts were compared over areas of tumour and adjacent normal tissue and were correlated with histology from the resected specimens to confirm the presence or absence of cancer. Blood samples were taken from pateints at intervals following injection (2 minutes-96 hours). Fresh samples of tumour and normal tissue from resected specimens were assayed for radioactivity using a gamma counter (Pharmacia, U.K.) and expressed as counts per minute/gram. Tumour to normal tissue ratios were calculated and expressed as mean for each group. Slices of formalin-fixed tumour and normal tissues (0.2-0.5 cm thick) were exposed to phosphor storage plates for up to 3 weeks and the plates were scanned with a phosphorimager 400A (Molecular Dynamics, U.K.) to generate a digital map of the distribution of radioactivity. These distributions were correlated with photographic images to determine the specificity of radiolabelled antibody localisation in tumour. For the 20 patients no adverse events were observed. Plasma clearance of MFE-23 was biphasic with an α half life of 18 minutes and a β half life of 2.6 hours. Tumour to normal tissue ratios were 1.2:1 (24 hrs), 2.2:1 (48 hrs), 4.4:1 (72 hrs) and 4.2:1 (96 hrs). Taking all time points and all cancer lesions together RIGS was 88% accurate in identifying tumour deposits (true positive 79% + true negative 9%). No false positive cases were recorded. Phosphor images demonstrated a close correlation between tumour site and localisation of radiolabelled antibody. We conclude that RIGS with MFE-23 single chain Fv antibody is safe and effective in locating tumour deposits and 72 or 96 hours is the optimal after injection time for surgery. Including more patients at the optimal time points will establish the utility of RIGS in identifying occult tumour which can be resected or for demonstrating extensive disease for which major surgery is not appropriate.

### 2.8 TAMOXIFEN IS ANTI-ANGIOGENIC *in vitro* AND ATTENUATES VEGF-MEDIATED ANGIOGENESIS *in vivo* WITHOUT ADVERSE EFFECTS ON WOUND HEALING.

DA McNamara*[1], J Harmey[1], JH Wang[1], D Donovan[1], E Kay[2], TN Walsh[1], DJBouchier-Hayes[1]. RCSI Departments of Surgery[1] and Pathology[2], Beaumont Hospital, Dublin.

Angiogenesis is essential to tumour growth. We have previously shown that surgery results in a permissive environment for angiogenesis, mediated in part by vascular endothelial growth factor (VEGF), and may therefore paradoxically increase micrometastatic growth. We have previously demonstrated that tamoxifen reduces serum VEGF in cancer patients. We hypothesise that tamoxifen inhibits VEGF-mediated angiogenesis. Our aim was to evaluate the effect of tamoxifen on angiogenesis and to assess whether its peri-operative use has detrimental effects on wound healing.

Human microvessel endothelial cells (HMEC) and human umbilical vein (HUVEC) endothelial cells were cultured *in vitro* and treated with tamoxifen in DMSO (100 ng/ml - 2.5μg/ml) or DMSO alone. Endothelial cell proliferation at 24 and 48 hours was quantified using 5-bromo-2'-deoxyuridine labelling. HMEC, but not HUVEC, proliferation was attenuated by tamoxifen *in vitro* at a dose of 2.5 μg/ml when compared to controls (p=0.001). Adult male Sprague-Dawley rats were pre-treated with po tamoxifen or placebo for 3 days and then injected with VEGF-impregnated Matrigel. Tamoxifen or placebo was continued for 5 further days. Microvessel density within the gel was quantified. Mean microvessel count in tamoxifen-treated VEGF gels was decreased when compared to controls (52.6 per high powered field (hpf) vs 63.3 per hpf, p< 0.05). Endothelial cell migration *in vivo* was significantly reduced by tamoxifen therapy (p<0.05). Laparotomy and small bowel anastomosis were performed on animals pre-treated with tamoxifen or placebo for 3 days. Therapy was continued up to 10 days post-operatively. There was no difference in macroscopic indices of wound healing between the two groups.

In summary, tamoxifen is anti-angiogenic *in vitro* and *in vivo* by reducing VEGF-mediated angiogenesis. This effect does not appear to impair wound healing in the perioperative period. Further evaluation is warranted to assess the potential role of neoadjuvant tamoxifen in dormancy therapy of micrometastases in the peri-operative period.

### 3.1 FUNCTIONAL EVIDENCE OF NOVEL TUMOUR SUPPRESSOR GENES FOR CUTANEOUS MALIGNANT MELANOMA.

C. N. Parris*, J. D. Harris and R. F. Newbold. Human Cancer Genetics Unit, Dept. Biology and Biochemistry, Brunel University, Cleveland Road, Uxbridge, Middx. UB8 3PH.

The incidence of skin cancer is rising faster than any other human malignancy with some 40,000 new cases each year. About 4,000 are the most aggressive form, Cutaneous Malignant Melanoma (CMM) which are responsible for 90% of deaths due to skin cancer (J. K. Rivers; *The Lancet* **347**: p803, 1996).

Cytogenetic analyses have identified non-random interstitial chromosome (chr.) deletions (Loss of Heterozygosity: LOH) associated with the development of CMM. Most prevalent are deletions of chr. 9p21 involving the tumour suppressor gene CDKN2 (p16) (M. Serrano *et al.*; *Nature* **366**: p704, 1993). However, deletion of chrs. 1p, 6q, 10q and 11q are common events in melanoma development. Evidence suggests that these regions contain critical TSGs central to CMM development. Moreover, while p16 is a well documented TSG for CMM and other tumours, it often remains intact and LOH occurs elsewhere on chr. 9p21 suggesting the presence of a further TSG on chr. 9p.

In this study, we have used a panel of human chrs. maintained as somatic cell hybrids in mouse A9 cells. Each human chr. is tagged with a retroviral vector expressing a Hygromycin$^{R}$-Thymidine kinase fusion gene allowing forward and reverse selection (A. P. Cuthbert *et al.*; *Cytogenet. Cell Genet.* **71**: p68, 1995). We have introduced additional normal human chrs. 1, 6, 9 (Δp16/p19) and 10 into the human melanoma cell line UACC-903, by Microcell-Mediated Chromosome Transfer (MMCT). Tumour suppression by the additional chrs. was determined by cloning efficiency of UACC-903 cells in soft agar (a highly reliable test for *in vivo* tumourigenicity). Our results have demonstrated suppression of the malignant phenotype with chrs. 1, 6 and 10. In addition, transfer of chr. 9 containing a micro-deletion that inactivates p16 and p19 still causes suppression, indicating the presence of an additional TSG on this chr.. Transfer of chrs. 5, 8, 15 and 9 (Δp arm) failed to suppress the melanoma cell line, as expected due to the absence of known melanoma TSGs.

Reversion of the hybrid melanoma cell lines by selection in Ganciclovir to remove the introduced chr. caused a reversion to the malignant phenotype (D. A. Trott *et al.; Mol. Carcinogenesis* **12**: p213, 1994).

In order to substantiate these findings, we are currently performing *in vivo* tumour studies in nude mice to demonstrate the tumour suppressive action of chrs. 1, 6, 9 (Δp16/p19) and 10 for CMM.

### 3.2 THE INSULIN-LIKE GROWTH FACTOR TYPE 1 RECEPTOR ACTS AS A NOVEL ONCOGENE IN COLORECTAL TUMOURIGENESIS.

A Allison, E Grant, G Wilson, K Mealey, M MacIntyre, CS McArdle & FK Habib, Department of Surgery, Western General Hospital, Edinburgh EH4

This study was designed to examine the role of the insulin-like growth factor type 1 receptor in human colon carcinogenesis. IGF-1 receptor expression was determined at the protein and mRNA levels in aberrant crypts, adenomas and adenocarcinomas from gross resected specimens and this was compared to adjacent normal epithelium. Immunocytochemistry has shown upregulation of cytoplasmic-membrane IGF-1R expression in aberrant crypts (5/5), adenomatous polyps (34/34) and adenocarcinomas (24/27) in comparison to adjacent normal epithelial cells for all specimens. The immunolocalization of IGF-1R was especially pronounced in the tumoural cell cytoplasm in comparison to the benign epithelial cytoplasm. There was no correlation between the level of IGF-1R expression and tumour stage. However, receptor upregulation was a feature of malignant transformation rather than epithelial cell proliferation where the authors found lower levels of expression in basal proliferative crypts and hyperplastic polyps (5/5). The validity of the result was supported by tissue positive and negative controls, reagent negative controls (where the antibody was peptide-absorbed with a purified receptor protein sequence) and Western blotting where there was complete molecular weight specificity for $\alpha+\beta$ subunits at 220kD in tumoural and normal tissue. Northern blotting for IGF-1R mRNA employing specific probes showed a similar difference between colonic cancers and normal epithelium.

These results show for the first time that IGF-1R upregulation is an important finding in the colonic polyp-cancer sequence and could be implicated in the mechanism of malignant transformation.

### 3.3 IDENTIFICATION BY DIFFERENTIAL DISPLAY OF NOVEL GENES EXPRESSED IN BREAST CANCER,

R. H. Kao*[1], R. Poulsom[2], A. H. Hanby[3], G. Francia[1] and I. R. Hart[1], [1]Richard Dimbleby Dept. of Cancer Research/ICRF Lab., St. Thomas' Hospital, London SE1 7EH, [2]Histopathology Unit, ICRF, London WC2A 3PX. [3]ICRF Clinical Oncology, Guy's Hospital, London SE1 9RT

In order to explore the *in vivo* changes in gene expression associated with breast tumour development, we have applied the rt-PCR-differential display technique to normal breast compared with a c-erbB-2 negative, grade 3 invasive ductal carcinoma from a single patient. Tumour and normal tissue were microdissected from consecutive frozen sections, mRNA was prepared and subjected to rt-PCR-differential display. By using 3 anchor primers and 30 arbitrary primers, 21 differentially expressed cDNA fragments were obtained in repeat procedures; 11 exhibiting enhanced expression in tumour material and 10 in normal material. These 21 bands were cloned and sequenced and 13 of these cDNA sequences were found to be novel according to GeneBank searches. One of these fragments, 12at2, expressed more highly in the tumour tissue was used as a probe in *in situ* hybridization analyses. In 6 individual breast cancer cases, including the original patient material from which it was derived; 12at2 was found to be expressed preferentially in invasive cancer cells relative to stromal, vascular endothelial, inflammatory and normal mammary glandular cells in which it was not expressed. One case of ductal carcinoma *in situ* also expressed 12at2. RT-PCR of ZR75 cells confirmed that 12at2 is expressed by this mammary tumour cell line and efforts are underway to isolate the full-length cDNA by screening a ZR75 cDNA library. These results show that the rt-PCR-differential display technique can be applied to breast cancer material to identify genes whose expression might be implicated in breast cancer progression.

### 3.4 CLONAL ANALYSIS OF BREAST DCIS USING DIFFERENTIAL METHYLATION OF *HUMARA*.

P.Costello*[1], A.H. McCann[1], E.Moore[1], Thomas Gorey[2], and P.A. Dervan[1].

[1]Dept. of Pathology and[2]Surgery, Mater Hospital and University College Dublin, Ireland.

Females inherit a maternal and paternal X chromosome, one of which is randomly inactivated in each cell by DNA methylation. *HUMARA* (Human androgen receptor gene Xq12) is heterozygous in 90% of individuals and is methylated on the inactive X chromosome. After treatment with a methylation sensitive enzyme (*Hha* 1), only *HUMARA* on the inactive X chromosome remains intact as methylation protects it from digestion. If tumour cells derive from one cell (clonal theory), the same X chromosome will be inactivated and only one allele can be PCR amplified following digestion (monoclonal pattern). Normal tissue on average contains a mixture of cells with either X chromosome inactivated resulting in the availability of both alleles for PCR amplification (polyclonal). The aim of this study was to assess clonality in different grades of ductal carcinoma in situ of breast by investigating the methylation status of *HUMARA* (Xq12).

Following microdissection of DCIS and normal ductal tissue from paraffin embedded samples, and PCR amplification with nested *HUMARA* primers, heterozygous samples were identified (n=11). These were *HhaI* digested, PCR amplified and electrophoresed. Monoclonality was found in 7/7 high grade and 2/2 intermediate grade. A polyclonal profile was found in 2/2 low grade tumours. In addition, monoclonal patches of normal ductal tissue were identified and microsatellite instability was found in 2 samples. In conclusion, it appears that high and intermediate grades DCIS are monclonal, while analysis of more low grade samples is required to establish whether they are polyclonal.

### 3.5 BRCA1 AND BRCA2 MUTATION SCREENING IN BREAST CANCER FAMILIES.

C.M. Phelan*[1], J.M. Lancaster[2], C. Gumbs[2], P. de los Rios[3], J. Abrahamson[3], E. Warner[3], N.A. Parfrey[1], P.A. Futreal[2], S.A. Narod[3]. [1]Dept. of Pathology, U.C.D., Ireland. [2]Dept. of Surgery, Duke Univ. Medical Centre, Durham, NC, USA.. [3]Womens College Hospital, Toronto, Ontario, Canada.

The BRCA1 and BRCA2 genes are inherited breast cancer genes. We are currently screening a panel of 233 breast (and other) cancer families to determine the proportion of breast cancer families attributable to these genes. The screening methods include SSCA of the coding regions including exon/intron boundaries, PTT of the large exons (exon 11 in BRCA1 and exons 10, 11, and 27 in BRCA2) and ASO's for the Ashkenazi BRCA1 mutations. So far,, 44 mutations have been found, 16 in BRCA1 and 28 in BRCA2. Moreover, 73/233 families are negative for BRCA1. Of the remaining 144 families, 106 have been screened by PTT of exon 11 but SSCA of the entire coding region has yet to be done. In addition, 176/233 families are negative for BRCA2 mutations as determined by combined SSCA and PTT, while a further 21 families have been screened by PTT only. All of the families are being screened for both genes.

There are a wide variety of other cancers present in these families. In the 38 families with male breast cancer, 0 BRCA1 and 8 BRCA2 mutations were observed. In the 45 families with ovarian cancer, 7 BRCA1 and 7 BRCA2 mutations have been seen so far, however, a greater number of families have been screened for BRCA2 mutations than for BRCA1. In he 13 families with pancreatic cancer, 1 BRCA1 and 4 BRCA2 mutations were observed. Twenty-three families revealed cases of bilateral breast cancer, 1 BRCA1 and 2 BRCA2 mutations were found in families with this feature. In summary, male breast cancer and pancreatic cancer appear to be indicative of BRCA2. The occurrence of bilateral cases of breast cancer does not appear to be wholly indicative of an inherited component, however, not all the families have been screened in both genes, so this conclusion is preliminary.

### 3.6 MUTATION ANALYSIS OF VON HIPPEL-LINDAU (VHL) TUMOUR SUPPRESSOR-ASSOCIATED PROTEINS,CUL-2 AND VBP-1, IN RENAL CELL CARCINOMA (RCC).

S.C.Clifford*[1], K. Hewson[1], S. Walsh[1], A. Brinke[2], P.M. Green[2], F. Gianelli[2], A. Pause[3], C. Eng[4] and E.R. Maher[1]. [1]Division of Medical & Molecular Genetics, Department of Paediatrics and Child Health, University of Birmingham, UK, [2]Division of Medical & Molecular Genetics, United Medical and Dental Schools, Guy's Hospital, London, UK, [3]Cell Biology & Metabolism Branch, NIH, Bethesda, MD, USA, [4]Dana Farber Cancer Institute, Boston, USA.

Inactivation of the von Hippel-Lindau tumour suppressor gene (VHL, TSG) is the most commonly detected genetic alteration in the most common form of kidney cancer (clear cell RCC (CC-RCC)). We have identified somatic VHL gene mutations in approximately 50% of CC-RCC and a further 15% of tumours have VHL gene hypermethylation. However the absence of VHL alterations in a significant proportion of CC-RCC suggests the presence of alternative genetic mechanisms for CC-RCC development. Mutations in pVHL interacting proteins such as CUL-2 and VBP-1 could be functionally equivalent to VHL inactivation and so suggest CUL-2 and VBP-1 as candidate RCC genes. To test this hypothesis we have mapped and determined the genomic structure of CUL-2 and performed mutation analysis on both genes. CUL-2 is a member of the cullins multigene family implicated in growth control in *C.elegans*, and VBP-1 maps to Xq21 and encodes a protein of unknown function. The CUL-2 gene was mapped to human chromosome 10p11.1 by FISH analysis, and a polymorphic marker from 10p11.1 (D10S176) showed LOH in ~10% of CC-RCC. To establish the CUL-2 genomic structure we have isolated a series of overlapping BAC and P1 clones by library screening. Direct DNA sequencing of these clones has to date identified 12 exons and their flanking intronic sequences (representing 69% of the full cDNA sequence). Mutational screening of the full-length cDNA sequence of each gene (by SSCP and direct DNA sequencing) is currently underway in a panel of 100 paired RCC and normal tissue DNA samples of known VHL TSG status to determine the roles of CUL-2 and VBP-1 in RCC pathogenesis, and the completed work will be presented.

### 3.7 THE VALUE OF RAPID FUNCTIONAL ASSAYS OF GERMLINE p53 STATUS IN LFS AND LFL FAMILIES.

RS Camplejohn[1], R Gilchrist[1], N Sodha[2], ME Lomax[1], PM Duddy[1], DM Barnes[3] and RA Eeles[2]. [1]Richard Dimbleby Dept. Cancer Res., UMDS, St Thomas' Hospital, London SE1 7EH; [2]The Cancer Genetics Team, Institute of Cancer Research and Royal Marsden Hospital Trust, Fulham Road, London SW3 6JJ and [3]ICRF Clinical Oncology Unit, Guy's Hospital, London SE1 9RT.

We have tested two rapid assays of p53 function, namely the apoptotic assay and the yeast-based FASAY as means of detecting germline p53 mutations in members of Li-Fraumeni and Li-Fraumeni like families. The first assay, the apoptotic assay was developed in our laboratory and measures the ability of p53 protein to direct DNA damaged cells into apoptosis following irradiation with 4Gy. The second test, the FASAY, is a yeast based assay which measures the ability of TP53 protein to transactivate a target gene. Results of the functional assays have been compared with direct sequencing or, in some cases, SSCP on material from 50 normal individuals and 22 members of Li-Fraumeni (LFS) or Li-Fraumeni like (LFL) families. The results show good agreement between the two functional assays and between them and sequencing/SSCP. All assays detected mutations in cells from 6 of the LFS/LFL family members. 16 LFL/LFS family members were not gene carriers. Thus, no false positives or negatives were seen with either functional assay although the apoptotic assay gave one borderline result for an individual without a p53 mutation. As an initial screen the apoptotic assay is not only rapid but cheap and very simple to perform. It would be expected to detect any germline defect that leads to loss of p53 function. The apoptotic assay could be ideal as a means of pre-screening large numbers of samples and identifying those which require further investigation. The FASAY detects mutations in exons 4-10, is rapid and it distinguishes between functionally important and silent mutations.

### 3.8 COLORECTAL TUMOURS IN THE FOLLOW-UP OF ASYMPTOMATIC HIGH-RISK INDIVIDUALS IN FAMILIES WITH HEREDITARY NONPOLYPOSIS COLORECTAL CANCER (HNPCC).

M.Ponz de Leon*[1], G.Della Casa[2], A.Percesepe[1], P.Benatti[1], R.Fante[3], C.Di Gregorio[4], and L.Roncucci[1]. [1]Dipartimento di Medicina Interna, Università di Modena; [2]Divisione di Medicina Interna, Ospedale S.Agostino, Modena; [3]Servizio di Anatomia Patologica, Ospedale di Mantova, [4]Servizio di Anatomia Patologica, Ospedale di Carpi (Modena).

In Hereditary nonpolyposis colorectal cancer (HNPCC) a close surveillance is usually proposed to high-risk family members with the objective of reducing cancer incidence and mortality. We identified 31 families with HNPCC and contacted a total of 223 first-degree relatives of affected individuals for counseling. Purposes of the present study were: A) to evaluate overall compliance among screened individuals and B) to assess the frequency and histologic features of neoplasms detected at endoscopy. Of the 390 subjects traced as "at risk" in the pedigree, 223 could be interviewed and colonoscopy strongly recommended; the subjects were re-evaluated at regular intervals. The 86 individuals who agreed to undergo endoscopic investigations were matched to controls of the same age and sex; these patients required endoscopy owing to minor rectal bleeding, constipation or abdominal discomfort. Results: Overall compliance was rather poor (86 patients, i.e., 38.6% of the interviewed and only 22.0% of the first-degree relatives). One or more colorectal lesions were found in 35 out of 86 (40.7%) family members and in 15 (17.4%, $P < 0.01$) controls. Among cases, 29 adenomas in 20 individuals were detected as opposed to 11 (in 9 subjects) in controls ($P < 0.05$). In asymptomatic family members, adenomas were significantly larger (9.1 ± 5 vs. 5.8% ± 3.7 mm, $P < 0.01$) and more frequently associated with a tubulovillous pattern and a high degree of dysplasia. Five carcinomas were detected among cases (four of which located between the cecum and the splenic flexure), but only one in controls. Conclusions: Genetic counseling can be carried out only in part of high-risk individuals in HNPCC families, since the majority of them cannot be reached or refuse to collaborate. The frequency of newly detected lesions and their possible aggressive behaviours render colonoscopy at regular intervals of time (1-2 years) highly recommended in these asymptomatic individuals at increased risk of cancer on a genetic basis.

### 4.1 BREAST CANCER CELL LINES EXPRESS SPECIFIC BINDING SITES FOR pLR , A NOVEL ANTI-PROLIFERATIVE PEPTIDE DERIVED FROM FROG SKIN VENOM

M.Boyer, H.W.van den Berg, C.Shaw*, M.Lynch and P.Johnston. Depts. Oncology and Medicine*, The Queen's University of Belfast

Drugs derived from natural products play an important role in the treatment of disease, including cancer. However, until recently large scale, systematic and rational drug discovery has not been undertaken. We have initiated studies of the anti-proliferative properties of natural peptides from a number of sources including those contained in the dermal venom of frogs.

We have previously shown that pLR, an 18 amino acid peptide, shows potent anti-proliferative effects towards a number of breast cancer cell lines. The most sensitive lines studied were an estrogen independent variant of the ZR-75-1 line (ZR-PR-LT) and a tamoxifen resistant variant of MCF-7, LY2. We have extended these studies to demonstrate anti-proliferative effects of pLR towards an ovarian carcinoma cell line PEO4.

We have successfully radio-iodinated pLR and used the product in radioligand binding assays. Cells in monolayer were exposed to a range of concentrations of 125-I pLR ($10^{-12}$ - 6 x $10^{11}$M) in the presence and absence of an excess of unlabelled pLR to account for non-specific binding. All cell lines examined expressed specific binding sites for pLR. Specific binding of 125-I pLR to ZR-75-1 cells, a tamoxifen resistant variant ZR-75-9a1 and an estrogen independent variant ZR-PR-LT was non-saturable over the concentration range studied, precluding estimates of maximal binding capacity or receptor affinity. Differences in the level of specific binding between cell lines were observed, with the highest level of binding being shown by the ZR-PR-LT line, reflecting its greater sensitivity to the anti-proliferative effects of the peptide. In preliminary experiments, saturable binding of 125-I pLR to MCF-7 and LY2 cells has been shown. Binding sites have a high affinity for pLR (Kd 0.8 - 1.4 x $10^{-11}$M) and LY2 cells express a higher number of receptor sites per cell (740) than MCF-7 cell (450).

We conclude that human breast cancer cell lines *in vitro* express specific binding sites for the novel anti-proliferative peptide pLR and that ZR-75-1 cells and variants appear to express receptors with a lower affinity and/or greater capacity than MCF-7 or LY2 cells. Furthermore, the cell lines most sensitive to the anti-proliferative effects of pLR (ZR-PR-LT and LY2) appear to express higher numbers of receptors than their parent lines.

### 4.2 GLUCOCORTICOID MODULATION OF CYTOTOXICITY: DIFFERENCES BETWEEN THE ROS17/2.8 AND UMR106 OSTEOSARCOMA CELL LINES.

H. Robson*[1], E. Anderson[1], SM. Shalet[2] and OB. Eden[3]. [1]Dept. Clinical Research, [2]Dept. Endocrinology, [3]Dept. Paediatric Oncology, Christie Hospital NHS Trust, Manchester M20 4BX.

Multi-drug regimens, combining cytotoxic drugs with a variety of other agents including steroids, are increasingly being used in the treatment of paediatric malignancies. We have previously reported that the synthetic glucocorticoids prednisolone (Pred) and dexamethasone (Dexa) attenuate cytotoxicity and growth inhibition of normal epiphyseal chondrocytes induced by exposure to a variety of cytotoxic drugs. Here we demonstrate that these two agents do not have the same effects on the osteosarcoma cell lines UMR106 (pre-osteoblasts) and ROS17/2.8 (mature osteoblasts). UMR106 and ROS17/2.8 cells, grown in DMEM/F12 + 5%FCS, were pre-treated with Pred or Dexa (50µg/ml) for 72h and subsequently exposed to a variety of cytotoxic drugs in their $EC_{20-80}$ range in the absence or presence of Pred/Dexa for a further 72h. The drugs were cisplatin (cis; 0.5µg/ml), carboplatin (carb; 0.5µg/ml), etoposide (etop; 0.5µg/ml) and methotrexate (MTX; 0.05µg/ml). Changes in cell number were analysed at 0, 3 and 6 days using a standard 96 well plate proliferation assay.

Etop, carb, cis and MTX killed steroid pre-treated UMR106 and ROS17/2.8 cells at least as effectively as they killed untreated cells. Steroid co-treatment during exposure to etop, carb and cis further significantly enhanced cytotoxicity in ROS17/2.8 cultures by between 20-50% in most cases. However, co-treatment of UMR106 cells failed to increase drug cytotoxicity above that observed in either the pre-treated or control cultures. Similarly, MTX cytotoxicity was increased 4 fold when combined with either of the steroids in ROS17/2.8 cultures. Conversely, in UMR106 cultures the cytotoxic actions of MTX were significantly reduced upon co-treatment with either Pred or Dexa.

Thus, we have shown that glucocorticoid exposure of the osteosarcoma cell line ROS17/2.8 but not the UMR106 cell line or normal chondrocytes enhances the actions of several cytotoxic drugs. The contrasting effects of MTX on the two osteosarcoma cell lines also suggest that steroid-mediated alterations in chemosensitivity may be drug specific. In conclusion, differences clearly exist within normal and malignant cell types which may have important implications for glucocorticoid use in clinical therapy.

### 4.3 THE EFFECT OF INSULIN-LIKE GROWTH FACTOR-I ON THE PROLIFERATION OF HUMAN BREAST CANCER CELLS *IN VITRO* AND ON THE SECRETION OF INSULIN-LIKE GROWTH FACTOR BINDING PROTEIN-2

P.Maxwell* and H.W.van den Berg Dept. Oncology , The Queen's University of Belfast

The insulin-like growth factor (IGF) system of growth factors, receptors and binding proteins plays an important role in breast cancer cell proliferation. We have previously shown that development of anti-estrogen resistance and estrogen independence in human breast cancer cells *in vitro* is associated with changes in both the expression of receptors for IGF-I and the pattern of secretion of IGF binding proteins (IGFBP's). In particular, we have demonstrated that tamoxifen resistance and estrogen independence is associated with a reduction in the level of secretion of IGFBP-2. We have also shown that growth stimulation of MCF-7 cells by estradiol is accompanied by a reduction in secretion of IFGBP-2 whilst inhibition of cell proliferation by estradiol in tamoxifen resistant or oestrogen independent breast cancer cell lines is associated with an increase in IGFBP-2 secretion, suggesting a growth inhibitory role for this IGF binding protein.

In this study we have investigated the effect of IGF-I on cell proliferation and secretion of IGFBP-2 in a defined serum-free medium (SFM). Secretion of IGFBP-2 was significantly reduced in the oestrogen receptor (ER) positive cell lines, MCF-7 and ZR-75-1 ($p<0.05$). IGFBP-2 secretion was also significantly reduced by IGF-I in the tamoxifen resistant variant of MCF-7 (LY2) and in the estrogen independent variant of ZR-75-1 (ZR-PR-LT). This reduction in IGFBP-2 was accompanied by increased proliferation in SFM. IGF-I (5-50ng/ml) stimulated proliferation of ZR-75-1 over a 6 day period by up to 50% and was associated with a 7-fold decrease in IGFBP-2 secretion. Likewise, IGFBP-2 secretion by MCF-7, LY2 and ZR-PR-LT was reduced by 1.8-6 fold and proliferation was increased by 30-40%. In contrast, exposure to IGF-I resulted in an up to 1.4 fold ($p\leq0.05$)increase in IGFBP-2 secretion by a tamoxifen resistant variant of ZR-75-1 (ZR-75-9a1) while proliferation was reduced by up to 55%.

These findings provide further evidence to support a growth inhibitory role for IGFBP-2 and indicate that the development of anti-estrogen resistance and estrogen independence results in differential changes in IGFBP-2 secretion in response to IGF-I.

### 4.4 THE EFFECT OF TAMOXIFEN TREATMENT ON *MUC-1* EXPRESSION IN PRIMARY BREAST CANCER.

J.M. Hanson*[1], D.A. Browell[2], W.J. Cunliffe[2], J.R. Young[2], J.P. Pearson[3], J.S. Varma[1], A. Allen[3], M.J. Higgs[2], I. Brotherick[1]. Departments of [1]Surgery & [3]Physiology, University of Newcastle, and [2]Queen Elizabeth Hospital, Gateshead.

Increased *MUC-1* expression reduces cell-cell interaction, facilitating cell detachment thus increasing metastatic potential. *Tamoxifen* therapy decreases the incidence of both local and metastatic recurrence in breast cancer although the mechanisms of its action are not fully determined. We attempt, using a flow cytometric method, to establish the effects of *Tamoxifen* therapy on *MUC-1* expression.

Optimum staining methodology was determined, rendering cells fixed but permeable to the *NCL-MUC-1 (Novocastra)* antibody. Dual parameter labelling using a *Goat -anti-mouse-FITC* conjugated secondary antibody to detect the *NCL-MUC-1* primary antibody and propidium iodide to determine nucleated cells from debris were employed.

The effect of a three week preoperative tamoxifen treatment on *MUC-1* expression by primary breast tumours was examined. Patients were matched for Age, Tumour Grade and Axillary Status. Patients were treated with either 20 mg oral *Tamoxifen* per day (n=46) or received no medication (n=51) in the three week interval between assessment and surgery. Specimens were snap frozen at the time of surgery. The percentage of cells expressing *MUC-1* was determined compared to an isotype control. Fluorescent bead standards were used to quantify the number of equivalent soluble fluorescent molecules of *MUC-1*/cell.

A lower percentage of cells expressed *MUC-1* following three week *Tamoxifen* treatment 22%(95% confidence µ = 17.3% - 27.3%) versus 31%(95% confidence µ = 25.1% - 37.2%) (p=0.03, Mann Whitney) and lower levels of *MUC-1* expression were seen following *Tamoxifen* treatment 39,387 (31,407 - 47,331) molecules/cell versus 52,878 (95% confidence µ = 40,451 - 65,304) (p=0.03, Mann Whitney).

We have demonstrated that *MUC-1* levels can be monitored by flow cytometry. *MUC-1* levels in patients treated with tamoxifen for three weeks prior to operation are significantly lower than in a comparable group of patients receiving no treatment. *Tamoxifen* therapy in the pre-operative period significantly reduces the expression of *MUC-1* in primary breast cancer, which has been implicated in the evolution of metastasis.

### 4.5 ENHANCEMENT OF CYCLOPHOSPHAMIDE ANTI-TUMOUR ACTIVITY WITH BIOREDUCTIVE DRUGS AQ4N AND TIRAPAZAMINE.

S.R.McKeown, O. Friery. R.Gallagher, M.Murray, C.Clarke, L.H.Patterson*., D.G.Hirst. School Biomedical Sciences, University of Ulster, Jordanstown, N. Ireland; *Dept Pharmaceutical Sciences, De Montfort University, Leicester, U.K.

AQ4N(1,4-bis-{[2-(dimethylamino-N-oxide)ethyl]amino}5,8-dihydroxy-anthracene-9,10-dione) is a bioreductive drug which is reduced, in hypoxic cells, to AQ4, a stable DNA-affinic cytotoxin. We have previously shown that both AQ4N and Tirapazamine (Tira) enhance the anti-tumour effect of a single dose of radiation[1]. We have now tested *in vivo* the anti-tumour effect of AQ4N or Tira with cyclophosphamide (CPM) using a growth delay assay (T50/80 tumour grown in BDF mice) or a clonogenic assay (SCCVII tumour grown in C3H mice). Mice were treated, when the tumour reached a size of about $150mm^3$, with a single i.p. dose of AQ4N (50-150mg/kg) or Tira (25 or 50 mg/kg); CPM (50 or 100 mg/kg) was given i.p. at known times (see below) after the bioreductive drug.

A significant increase in anti-tumour effect was observed when either bioreductive drug was combined with CPM. In BDF mice there was a tumour growth delay of 15.8 days with AQ4N (p = .0001) and 11.5 days with Tira (p = .0013). Combination of CPM with AQ4N was found to be equivalent to a single 200mg/kg dose of CPM alone i.e. a 50% reduction in CPM dose for an equivalent anti-tumour effect. For Tira the effect was equivalent to 150mg/kg CPM. Normally drugs were administered 30min apart. Administering AQ4N 6h prior to CPM showed no appreciable difference in the anti-tumour effect obtained. A clonogenic assay was used to determine the effect of drug combinations in the SCCVII model. Tira provided a dose modification factor of approximately 1.7 at 50mg/kg CPM and about 1.4 when combined with 100mg/kg CPM (30 min or a 2.5h interval). AQ4N provided a dose modification of > 4 with 50mg/kg CPM and about 3 with 100mg/kg CPM (30min or 6h interval). In conclusion both Tira and AQ4N enhance the anti-tumour effect of CPM, with AQ4N showing a greater effect in both tumour models.

(1) McKeown SR, Hejmadi M, McIntyre IA, McAleer JJA, Patterson LH. (1995) Br J Cancer, 72,76 - 81.

### 4.6 HYPOXIA INDUCIBLE FACTOR-1: EFFECT ON TUMOUR BIOLOGY,

Gabi U. Dachs[1], Brian Marples[2], Kaye J. Williams[3], Ian J. Stratford[3] and Dai J. Chaplin[1] [1]Gray Laboratory Cancer Research Trust, Northwood HA6 2JR, [2]Paterson Inst. Cancer Research, Manchester M20 4BX, [3]Dept. Pharmacy, University of Manchester, Manchester M13 9PL

Solid tumours have regions of reduced oxygen (hypoxia) as they contain a poor and disorganised blood supply. Hypoxia inducible factor-1 (HIF-1) is the transcription factor that regulates gene expression in response to hypoxia. HIF-1 binding sites (HRE) have been found in regulatory sequences of variety of genes (Epo, VEGF, PGK, glut-3, etc.) and HIF-1/HRE gene regulation could be demonstrated in all mammalian cells tested to date. HIF-1 is a heterodimer consisting of HIF-1$\alpha$ and HIF-1$\beta$/ARNT basic-helix-loop-helix proteins. HIF-1$\beta$/ARNT can also dimerize with the dioxin receptor AHR to regulate the xenobiotic response.

We have previously shown that HIF-1 could regulate gene expression in solid tumours and influence tumour growth and angiogenesis (Maxwell *et al.*, 1997). Mouse hepatoma cells Hepa-1 containing wild type HIF-1 were compared to Hepa-1 c4 mutant cells which lack HIF-1$\beta$/ARNT. Xenograft tumours of c4 grew more slowly and showed reduced vascularity compared Hepa-1 wt. No difference in their growth *in vitro* was observed both in air and at 1% oxygen.

To analyse the effect of HIF-1$\beta$/ARNT on tumour biology further, the hypoxic content of tumours was estimated by two different methods. Radiation (10Gy) followed by single cell electrophoresis (the comet assay, Olive 1995) indicated that tumours grown from c4 cells had a lower hypoxic fraction than those grown from Hepa-1 wt cells, since they showed more single strand DNA breaks than the wt cells. Initial *in vivo* radiation sensitivity assays, however, showed no difference in surviving fraction following 10Gy radiation, although clonogenicity from the c4 tumours was only about 10 % of the wt. Clonogenicity from *in vitro* radiation experiments were comparable, and c4 showed a marginal increase in sensitivity to radiation compared to Hepa-1 wt. Treatment of tumour-bearing mice with the hypoxia marker NITP (Hodgkiss *et al*, 1995) showed no overall difference in staining between wt and HIF-1$\beta$/ARNT Hepa-1 tumour sections, although there was considerable intertumour variation within each group. Oxygen measurements using polarographic and fibre optic technology are now planned to directly determine tumour oxygen status.

### 4.7 SELECTIVELY ACTIVATING BIOREDUCTIVE DRUGS IN-VITRO USING GENE THERAPY,

M.P.Saunders[1], A.V.Patterson[1], P.J.Ratcliffe[2], A.L.Harris[2], I.J.Stratford[1], [1]Dept of Pharmacy, University of Manchester, M13 9P. [2]Institute of Molecular Medicine, John Radcliffe Hospital, Oxford, OX3 9DU.

The treatment of patients with conventional chemotherapy is sometimes associated with severe systemic toxicity and only a minimal survival benefit. Because of this, attempts have been made to specifically target the tumour, to increase cell kill but to spare the surrounding normal tissues. Bioreductive drugs are prodrugs that are activated in hypoxic regions of tumours by a range of reductases including NADPH:P450 reductase. This tumour specific metabolism provides the basis for therapeutic differential. When the cDNA for P450 reductase, under the control of the moloney leukaemia virus LTR, was transfected into the MB-MDA-231 breast cancer cell line, clones over-expressing P450 reductase were generated, which were up to 50 times more sensitive to bioreductive drugs than the wild type cell line under hypoxic conditions. It has previously been reported that certain sequences of DNA can be used to drive gene expression during or following hypoxia (Dachs et al, Nat Med 3, 5; 515-520,1997) or irradiation (Weichselbaum et al, IJROBP 30,1; 229-234 1994). We have ligated concatimers of regulatory sequences of cDNA to marker genes and also the gene encoding P450 reductase in to a mammalian expression vector system. The enhancers we have used include the hypoxia responsive elements (HRE) of phosphoglycerate kinase 1 (PGK1) and erythropoietin (EPO) as well as Nuclear factor kappa B (NFkB), activator protein 1 (AP-1) and Early growth response gene 1 (Egr-1). Egr-1 has been shown to be radiation inducible whilst NFkB and AP-1 are potentially activated by both stimuli. Using CD4 as a marker we have shown that a vector containing a pentamer of the Epo HRE and the Egr-1 promoter is both radiation inducible and hypoxically activated. We have also shown that P450 reductase activity can be induced in hypoxic conditions when under control of both an AP-1 trimer or a NFkB/PGK1 pentamer. Such clones have been shown to be more sensitive to bioreductive drugs after hypoxic incubation. Further evaluation of these enhancer / promoter systems is under-way. (MPS is a MRC clinical training fellow).

### 4.8 BIODISTRIBUTION OF ENZYME ACTIVITY OF A FUSION PROTEIN FOR USE IN ADEPT.

S.K. Sharma*[1], J. Bhatia[1], R.B.Pedley[1], D.A.Read[1], P. Michael[2], R.W. Boden[1], K.A. Chester[1] & R.H.J. Begent[1] [1]CRC Targeting and Imaging Group,Dept. of Clinical Oncology, Royal Free & UCL Medical Schools, London NW3 2PF. UK. [2]CAMR, Salisbury.

Antibody directed enzyme prodrug therapy (ADEPT) involves targeting of an enzyme specifically to tumour sites where it converts a relatively non-toxic prodrug to a potent cytotoxic drug.

A recombinant fusion protein, comprising carboxypeptidase G2 (CPG2) enzyme and the anti-CEA scFv MFE-23 derived from a phage display library, has been expressed in *E. coli.* The biodistribution of enzyme activity of this fusion protein has been studied in the LS174T human colon adenocarcinoma in nude mice in comparison with free CPG2. Mice were injected with either MFE-CPG2 or free CPG2 ,1000units CPG2/kg per mouse. Tumour, plasma and other normal tissues were collected over time, homogenised, and in vitro turnover of CPG2 substrate measured by HPLC. CPG2 concentration in tissue homogenates was calculated. The percentage injected enzyme activity per gram at 6, 24 and 48hrs in tumour was 28%, 21% and 9% respectively for the fusion protein compared to 10.28%, 2.184% and 0.34% for the free CPG2. The corresponding plasma levels were 18%, 2% and 0.49% for the fusion protein and 10.28%, 0.87% and 1.2% for free CPG2. This gave tumour to plasma ratios of 1.5, 10 and 19:1 for the fusion protein and 0.77, 2.5 and 0.28 for the free CPG2 group. Therefore at 48hrs after injection, 26 times more CPG2 was present in the tumour with the fusion protein than with free CPG2 alone without corresponding increase in normal tissues. The enzyme concentration measured in tumours compares favourably with that observed with A5B7-F(ab')2-CPG2 conjugate used in our ADEPT pre-clinical and clinical studies with faster plasma and normal tissues than is found for the conjugate.

Whereas the fusion protein cleared rapidly from normal tissues, high levels of enzyme activity were retained at the tumour site, resulting in tumour to liver, kidney and lung ratios of 371:1, 562:1 and 450:1 respectively at 48hrs. These results indicate that the MFE-23-CPG2 fusion protein has the required characteristics for ADEPT with high tumour selectivity and efficacy.

Supported by the Cancer Research Campaign .

### 5.1 LOW LEVELS OF uPA IN BASAL CELL CARCINOMAS OF THE SKIN : *A POSSIBLE REASON FOR THEIR FAILURE TO METASTASISE?*

T. Maguire*[1], D. Chin[2], D.S. Soutar[2], T. O' Reilly[1] & M.J. Duffy[1], [1]St. Vincent's Hospital, Dublin & [2]Canniesburn Hospital, Glasgow.

Urokinase plasminogen activator (uPA) is a serine protease causally involved in cancer invasion and metastasis. *In vivo*, uPA acts by binding to a membrane-bound receptor, termed uPAR, while its catalytic activity is controlled by the inhibitor PAI-1. Basal cell carcinomas (BCC) of the skin represent one of the most frequent forms of cancer in this country. These tumors are unique in that they exhibit low rates of formation of distant metastasis. We hypothesise that this inability of BCCs to give rise to distant metastasis may be due either to low levels of uPA or high levels of PAI-1 within the BCCs. The aims of this study were, therefore, to assay uPA, uPAR and PAI-1 within the BCCs and to compare these concentrations with levels present in other skin tumors such as squamous cell carcinomas (SCCs) and malignant melanomas and also with levels in benign skin lesions.

uPA, uPAR and PAI-1 were extracted from benign skin lesions (n=45), BCCs (n=52), SCCs (n=26) and malignant melanomas (n=16) using 1% Triton X-100 in a 50 mM Tris-HCl buffer (pH 7.4) and then assayed by ELISA (American Diagnostica). Protein levels were estimated using the BIORAD assay.

Median levels of uPA were similar in benign lesions (0.053 ng/mg) and BCCs (0.063 ng/mg). uPA levels, however, were significantly higher in both SCCs (0.169 ng; p=0.0031) and malignant melanomas (0.572 ng/mg; p=0.0001) compared to concentrations in BCCs. Similarly, levels of uPAR were significantly higher in SCCs and malignant melanomas compared to BCCs. Finally, median levels of PAI-1 were lower in the BCCs (0.927 ng/mg) than in SCCs (1.465 ng/mg; p=0.080) and were approximately 5 times lower in the BCCs compared with malignant melanomas (p=0.0001).

We conclude from this study that the failure of BCCs to metastasise is unlikely to be due to the presence of excessive levels of PAI-1. On the other hand, the findings of this study suggest that low levels of uPA, its receptor (uPAR) and perhaps PAI-1 contribute to the low metastatic potential of BCCs.

### 5.2 DIFFERENTIAL UPREGULATION OF MMP-9 IN SQUAMOUS CELL CARCINOMAS OF HEAD AND NECK BY EGFR LIGANDS.

P. O'charoenrat, S. Eccles, W. Court, G. Box, H. Modjtahedi and C. Dean. McElwain Laboratories, The Institute of Cancer Research, Sutton, Surrey SM2 5NG.

Squamous cell carcinomas of the head and neck region are characterised by a marked propensity for local invasion and dissemination to cervical lymph nodes; distant metastases are found in 30-40% of cases. Tumours over-expressing the epidermal growth factor receptor (EGFR) and/or one or more of its ligands (EGF, TGFα, HB-EGF, amphiregulin and betacellulin have a higher probability of invasion and metastasis. Matrix metalloproteinases (MMPs) are key enzymes involved in not only invasion of tumour cells through stroma and endothelia during extravasation of secondary sites, but also in the penetration of capillary sprouts during neoangiogenesis, critical steps in metastasis development. We set out to examine, in established squamous cell carcinoma cell lines (e.g HN5, HN6, Detroit 562, RPMI 2650) and in cells newly derived from patients with head and neck cancer (ICR006), the relationship between EGFR expression and MMP (particularly gelatinase) activity, and the effects of different EGFR ligands. We found that the levels of MMP-9 (gelatinase B) pro-enzyme and active enzyme was highest in cells with the highest EGFR expression as judged by zymography. Similar results were seen with MMP-2 (gelatinase A), although the enzyme levels were lower, and active enzyme was rarely seen. Incubation of the cell lines with EGFR ligands upregulated MMP-9 (but not MMP-2) expression in a dose dependent manner, the effects being most marked in cells with high EGFR, and negative in cells with no over-expression of this receptor. In addition, we found that betacellulin consistently increased MMP-9 expression and activation more than the other four ligands when tested at equimolar concentrations. All ligands induced morphological changes in HN5 carcinoma cells, consistent with an "epithelial-mesenchymal transition" and increased motility. The ligands had no demonstrable direct effect on the MMP profiles of fibroblasts derived from head and neck cancers which showed constitutive expression of latent and active MMP-2. However, co-culture of fibroblasts and tumour cells also led to increased MMP-9 activity, consistent with previously described paracrine interactions. Further work will aim to define the effects of specific EGFR ligands on other members of the MMP family in larger panels of squamous cell carcinomas; characterise their invasive capacity, and examine the potential therapeutic effects of monoclonal antibodies and MMP inhibitors.

### 5.3 MATRIX METALLOPROTEINASES ARE OVEREXPRESSED IN COLORECTAL CANCER.

H.M. Collins*, G.M. Tierney, S.A. Watson.
The Academic Unit of Cancer Studies, Department of Surgery, University Hospital, Nottingham.

Introduction: Matrix Metalloproteinases (MMPs) are a family of proteolytic enzymes which work in concert to degrade components of the extracellular matrix. These invasive enzymes have been implicated in colorectal cancer progression. Their action is regulated by the naturally occurring inhibitors, TIMPs.

Aims: To establish a quantitative profile of MMPs and TIMPs in paired normal mucosa and colorectal carcinomas.

Methods: Normal and tumour samples were obtained from patients undergoing routine resection. mRNA was extracted from tissue using guanidium thiocyanate, purified and reverse transcribed to double stranded cDNA. Competitive PCR was performed with the following primer pairs; MMP's-1,-2,-3,-7,-9,-10,-11, MT-MMP-1,-2, TIMPs -1,-2 and GAPDH. The technique relies on a competition reaction in which a synthetic multi-competitor cDNA standard is co-amplified with target cDNA in the same PCR reaction. MMP mRNA levels were expressed relative to GAPDH.

Results: mRNA was detected for all the MMPs and their inhibitors in the tumour samples. Levels were lower in the normal tissue with MMP-10 detected in only one sample. Levels were significantly different in the tumour samples when compared to normal tissue for MMPs -1, -3, -7, -9 and -11 (p = 0.0294, p = 0.0294, p= 0.0304, p= 0.0304, p= 0.0294, Mann Whitney U non -parametric test). MMP-2, the membrane type metalloproteinases and their specific inhibitors were not significantly different.

Conclusions: 'Net proteolytic' activity is potentially greater in the tumour samples which may be significant in the progression of colorectal cancer.

### 5.4 INCREASED EXPRESSION OF GELATINASES (MMP-2 & MMP-9) IN HUMAN COLORECTAL CANCER.

C Keh*[1,3], T Ismail[1], PG de Takats[2], A Martin[3], MJO Wakelam[3] and DJ Kerr[3]. [1]Dept. of Surgery , [3]CRC Institute for cancer Studies, University Hospital, Birmingham B15 2TH, [2]Oncology Centre, Addenbrooke's Hospital, Cambridge, CB2 2QQ.

In colorectal carcinogenesis, it appears that normal epithelial cells undergo dysplastic change to form a benign tumour (adenoma), which, over time, becomes malignant. Malignant progression requires degradation of basement membrane to facilitate invasion into surrounding tissues. The matrix metalloproteinases, MMP-2 (gelatinase A) and MMP-9 (gelatinase B) degrade type IV collagen, the main component of basement membrane and may play a role in tumour invasion and metastasis. We undertook to determine the relationship between gelatinase expression and malignant transformation in human colorectal cancer.

100 paired colorectal tumour and normal mucosa samples were fresh-frozen and homogenised prior to analysis. Gelatinase content was measured by zymography and quantitated by computer assisted densitometry. Both the inactive 72kDa proform and cleaved, active 62 kDa form of MMP-2 could be detected, while only a single band representing the inactive form of MMP-9 was evident. Statistical analysis of tissue gelatinase content was performed by comparing the paired normal with tumour samples using Wilcoxon signed rank test and correlating the tumour/normal ratios with tumour progression (ie. normal, adenoma, carcinoma and Dukes' stage).

| Tumour stage | MMP-2 proenzyme | MMP-2 active form | MMP-9 proenzyme |
|---|---|---|---|
| Adenoma (n=7) | 1.15 (p=0.17) | 1.39 (p=0.38) | 1.28 (p=0.11) |
| Dukes' A (n=15) | 1.55 (p=0.005) | 2.46 (p<0.005) | 2.08 (p=0.001) |
| Dukes' B (n=34) | 1.14 (p=0.01) | 3.10 (p=0.0001) | 2.05 (p=0.0001) |
| Dukes' C (n=44) | 1.24 (p<0.05) | 3.08 (p=0.0001) | 1.70 (p=0.0001) |

Median tumour/normal ratios of MMP-2 and MMP-9 activities shown

MMP-2 and MMP-9 were present in higher amounts in tumour tissue compared with normal mucosa in all cases. This was not statistically significant for adenomas, probably reflecting small numbers analysed. There was however, a trend towards a correlation between increasing active MMP-2 content and tumour progression (ie. normal versus adenoma versus carcinoma). There was no significant correlation with Dukes' stage.

MMP inhibitors are currently being tested in clinical trials of cancer patients. However their clinical application is being hampered by systemic toxicity which may be attributable to lack of specificity against a particular class of MMP. Our study supports development of specific gelatinase inhibitors which may afford greater clinical utility.

### 5.5 THE EFFECT OF ADENOVIRAL GENE TRANSFER OF MMP AND TIMP ON A RENAL CELL LINE.

A.M McElligott*, A.H. Baker[1] & H. McGlynn, School of Biomedical Sciences, University of Ulster, Coleraine BT52 1SA, [1]Bristol Heart Institute, Bristol Royal Infirmary, Bristol BS2 8HW.

The ability of a cancer cell to invade tissue surrounding the tumour is a critical step in the metastatic process. The extracellular matrix (ECM) forms a physical barrier to this tumour cell invasion. Members of the matrix metalloproteinase (MMP) family of extracellular endopeptidases are the only known enzymes capable of degrading native collagens and therefore play the primary role in ECM remodelling, and have been implicated in the metastatic process in some cancers. The activity of MMPs is tightly regulated with important negative regulators being the tissue inhibitors of metalloproteinases (TIMPs). In this study we have used recombinant adenoviral vectors to transfect the renal cell carcinoma cell line, caki-1, with the genes for MMP-9 and TIMP-1 and -2. Increases in MMP-9 and TIMP-1 and -2 levels were determined by RT-PCR, gelatin zymography, immunoblotting and quantitative ELISA. The effect of increasing the expression of these genes on the invasive potential of the caki-1 cell line was investigated using an *in vitro* invasion assay which determines the ability of the cells to degrade and migrate through an ECM boundary. The invasive potential of the cells was shown to be altered following transfection with the MMP and TIMP genes. In particular the cells showed a dose responsive decrease in invasion potential dependent on the multiplicity of infection with adenoviral vector for the TIMP genes. These results suggest that MMP-9 and TIMP-1 and -2 have a role in the invasive ability of the caki-1 cell line and that recombinant adenoviral vectors are an efficient system for studying the role of MMPs and TIMPs in this process *in vitro*. These vectors will be a useful tool for *in vivo* studies of the role of MMPs and TIMPs in the invasive and metastatic process.

### 5.6 SIGNALLING FROM THE $\beta_1$ INTEGRIN AND HEPATOCYTE GROWTH FACTOR IN TWO METASATIC CELL LINES: A ROLE IN CELL MOTILITY AND INVASION.

V.G. Brunton*, I. McNairn, A.W. Wyke and M. Frame, Beatson Institute for Cancer Research, Garscube Estate, Switchback Road, Glasgow G61 1BD.

Two cell lines were established from metastatic lesions taken from the liver (ALT-F) and the lymph node (ALT-G) of a patient harbouring a primary colon tumour (Gregoire *et al.*, Inv. Metast. 13:253,1993). Differences in the ability of these two cell lines to move in a wound healing assay and invade *in vitro* into Matrigel in response to hepatocyte growth factor (HGF) were observed. The ALT-G cells were able to move and invade in response to HGF while the ALT-F cells were not. Both cell lines were able to respond mitogenically to HGF indicating that a downstream component of the HGF signalling pathway that can distinguish between a mitogenic and motogenic response may be altered in the two cell lines. Integrins allow cells to adhere to extracellular matrix proteins and are involved in both the invasion and motility of cells. An antibody to the $\beta_1$ integrin was able to block HGF induced motility and invasion in the ALT-G cell line. As a number of studies have indicated that cross-talk occurs between integrin and growth factor signalling pathways we decided to look at this in our two cell lines. HGF and a $\beta_1$ integrin signal induced phosphorylation of focal adhesion kinase and activation of MAP kinase in both cell lines. No synergistic response was seen in the presence of both HGF and a $\beta_1$ integrin signal. Furthermore HGF treatment did not alter the expression of a number of integrin sub-units including $\beta_1$ integrin.

We are currently looking at other downstream components of these signalling pathways such as $p130^{cas}$ and c-Src to determine whether differences do occur between the cell lines which may account for the differences observed in the motility and invasion of the two cell lines.

### 5.7 Tumor Promoters, TPA and Okadaic acid, Regulate the Expression of α2β1 Integrin.

*Nissinen, L., Westermarck, J., Koivisto, L., Kähäri, V-M. and Heino, J., MediCity Research Laboratory, University of Turku, Turku, Finland.

Integrin α2β1 is a heterodimeric transmembrane receptor for various collagen types and, at least in some cell types, for laminin and tenascin. We have shown, that in osteogenic cells the expression of α2β1 integrin is induced by both Kirsten sarcoma virus and chemical transformation (Santala *et al., J. Biol. Chem.* 269: 1276-1283, 1994). The association of α2 integrin with transformed cell phenotype was studied further. α2β1 integrin is a type I collagen receptor of human osteogenic MG-63 cells. In the presence of TPA MG-63 cells showed increased adhesion on type I collagen, whereas okadaic acid decreased the attachment of cells to several matrix molecules, including type I collagen. As shown by flow cytometry okadaic acid had little effect on the cell surface expression of α2 integrin subunit although its mRNA levels and the synthesis of the corresponding protein increased. TPA was potent inducer of α2 integrin expression at protein and mRNA level. The induction of α2 integrin was seen also on cell surface. The two agents had opposite effects on the α3 integrin; TPA induced and okadaic acid suppressed its expression. expression. Nuclear run on assay showed, that in MG-63 and HT-1080 cells α2 integrin gene is regulated at transcriptional level by TPA and okadaic acid. Dexamethasone, which is a potent AP-1 inhibitor, prevented the effect of both factors on α2 integrin expression. Transient transfection assays with α2 integrin promoter and an AP-1-dependent control promoter linked to a reporter gene, as well as experiments with cells lines other than MG-63, indicated that AP-1 activation alone does not explain the induction of α2 integrin gene expression.

### 5.8

**INTERLEUKIN-1 UPREGULATES EXPRESSION OF CD44 AND ITS METASTASIS-ASSOCIATED VARIANT V6 IN THE HUMAN ENDOTHELIAL CELL LINE ECV304 BY A MECHANISM INVOLVING THE TRANSCRIPTION FACTOR EGR-1.**

K. Fitzgerald and L.A.J., O' Neill, Dept. Biochemistry and Biotechnology Institute, Trinity College, Dublin 2.

Tumour cells may acquire, and use for their metastatic spreading, properties which lymphoid cells have developed to defend against pathogens. The cell adhesion molecule CD44 is a ubiquitously expressed multifunctional glycoprotein, involved in lymphocyte-endothelial cell interactions (as a lymphocyte homing molecule), cell adhesion, T-cell activation and tumour metastasis. Here, we identify CD44 as a downstream target of the pro-inflammatory cytokine interleukin-1, (IL-1), in the endothelial cell line ECV304. IL-1 increased expression of the 85 kDa standard CD44 above basal levels. Interestingly, a 180-200 kDa CD44 variant 6 isoform, (which has been linked with tumour metastasis [1] was also upregulated, as determined by ELISA and western immunoblotting. Analysis of mRNA expression by RT-PCR and northern blotting using CD44 standard and variant exon 5 specific primers indicated that IL-1 increased CD44 gene transcription. Transfection of CD44 promoter- reporter constructs demonstrated an induction of transcriptional activity following treatment of cells with IL-1α. The EGR-1 binding motif within the promoter was shown to be necessary for stimulus-induced expression as mutation of this site abolished transcriptional activity following treatment with IL-1α. Egr-1 protein accumulated following IL-1 treatment and IL-1 was shown to activate Egr-1 consensus site-specific DNA binding, as shown by carrying out an electrophoretic mobility shift assay with a probe containing the Egr-1 binding site. Taken together, these studies identify EGR-1 as an intermediary linking IL-1 receptor derived signals to the induction of CD44.

[1] Gunthert U., Hofmann, M., Rudy, W., Reber, S., Zoller, M., Haubmann, I., Matzku, S., Wenzel, A., Ponta, H., and Herrlich, P. 1991, Cell (65), 13-24.

KEYWORDS CD44 Interleukin-1 Metastasis Egr-1

### 5.9 DISSASSEMBLY OF ENDOTHELIAL CELL-CELL ADHERENS JUNCTIONS FOLLOWING ADHERENCE OF HUMAN BREAST ADENOCARCINOMA CELLS.

J. Cai, W.G. Jiang and R.E. Mansel, Metastasis Research Group, Department of Surgery, University of Wales College of Medicine, Cardiff. CF4 4XN.

Tumour cell extravasation from the circulation is accompanied by an increase in the permeability of the vascular endothelium. Adherens junctions play a key role in the maintenance of endothelial cell permeability and are comprised of the vascular endothelial (VE)-cadherin/catenin adhesion complex. In this study, the human breast cancer cell line, MDA MB231, was co-cultured with a monolayer of human umbilical vein endothelial cells (HUVEC) and the resultant effects on the endothelial adherens junction integrity investigated. Using immunofluorescence analysis we observed that, following tumour cell adherence to the HUVEC, there was a rapid (within 5 minutes) redistribution of VE-cadherin, resulting in its loss from regions of endothelial cell-cell contact. These effects were transient since VE-cadherin gradually reorganised within the endothelial cell contacts after this time. Following immunoprecipitation of VE-cadherin from the HUVEC cultured with MDA MB231 for different time periods (0min, 5mins, 30mins and 1hr) and subsequent immunoblotting, we observed that the overall expression of VE-cadherin did not change, however, the amount of β-catenin markedly decreased after 5mins of tumour cell adhesion to the HUVEC. Immunoprobing of these samples with anti-phosphotyrosine antibodies demonstrated that the tyrosine phosphorylation of VE-cadherin was significantly increased following 5mins of tumour cells adhesion. Together, these results suggest that the adhesion of tumour cells to HUVEC promotes the redistribution of VE-cadherin from inter-endotheiial adherens junctions, an effect which may be attributed to the increase in tyrosine phosphorylation of members of the VE-cadherin/catenin adhesion complex. This, in turn, may increase vascular endothelial permeability and facilitate the transendothelial migration of tumour cells during extravasation.

### 5.10 EXPRESSION OF THE SERPINS SCCA AND LEUPIN IN NORMAL AND MALIGNANT SQUAMOUS EPITHELIUM OF THE UTERINE CERVIX.

J. Coulter *, R.C. Barnes and D.M. Worrall. Department of Biochemistry, University College Dublin, Dublin 4, Ireland.

At present there is no reliable tumour marker to aid the gynaecological oncologist in the management of patients with carcinoma of the uterine cervix. One protein which has been investigated as a tumour marker is the squamous cell carcinoma antigen (SCCA) which is a member of the serpin superfamily of protease inhibitors. Serum levels of SCCA can correlate with late stages of disease and a role for SCCA in tumour development has been proposed. An immunoassay detection system for serum SCCA has been developed but its sensitivity and specificity are unclear.

We have discovered a related serpin in HeLa cells, leupin, which is high in sequence identity (92%) to SCCA, but which contains differences in the serpin reactive loop sequence.(Barnes, R.C. and Worrall, D.M. (1995) *FEBS Lett.*, 373, 61-65). The degree of sequence similarity between the two serpins means that previous patient studies on SCCA expression are likely to have detected both SCCA and leupin. We have evidence that the IMx serum immunoassay for detection of SCCA will also detect leupin, and that leupin may be the fraction previously considered to be an acidic isoform of SCCA. An ability to distinguish between these gene products may provide better insight into their potential role in tumour development and lead to greater efficacy in tumour marker detection.

In this study we have designed specific oligonucleotide primers to detect the individual expression of SCCA and leupin in human tissues. Biopsies of normal and malignant uterine cervix squamous epithelium were excised from patients at the time of hysterectomy and both genes were amplified from total RNA by RT-PCR. Both leupin and SCCA are expressed in normal and malignant squamous epithelium of the uterine cervix. However, SCCA levels were greater in normal cervix, and although both gene products were elevated in tumour tissue, leupin showed a more significant increase. These findings suggest that leupin may be a more important marker for cervical carcinoma.

### 6.1 A HISTONE DEACETYLASE INHIBITOR INDUCES SENESCENCE BY AN INK4 AND P53-DEPENDENT MECHANISM.

J.Munro, H.Ireland, V.Morrison and E.K Parkinson*, CRC Beatson Laboratories, Garscube Estate, Switchback Rd, Bearsden, Glasgow, U.K.

The histone deacetlyase inhibitor sodium dibutyrate (SDB) has been reported to cause a phenotype that is biologically reminiscent of senescence in human fibroblasts (Ogryzko et al, Mol. Cell Biol., 16, 5210-5218, 1996) but remarkably little is known of its mechanism of action. We have extended the earlier observations to show that SDB produces a phenotype which is biologically, biochemically and genetically inseparable from replicative senescence. In particular, the SDB-treated cells upregulated the cell cycle inhibitors $p21^{waf1}$ and $p16^{ink4a}$ in the same sequential order as in senescence and the cyclins A, B and their associated kinases cdc2 and Cdk2 were downregulated, resulting in an underphosphorylation of pRb and a G1 arrest. Significantly, p53 protein was not induced, and this, together with the observed accumulation of $p16^{ink4a}$ in SDB-treated fibroblasts argues against the results being a consequence of DNA damage. The programme was severely abrogated by SV40 Tantigen in both human and mouse fibroblasts and in most established cell lines. Experiments with nullizygous mouse cells showed that both the ink4 locus and p53 were required for optimum inhibiton of proliferation but that p21-deficient cells were sensitised to SDB-induced apoptosis which normally occurs at much higher doses. The results indicate that SDB may mimic a form of senescence that does not require telomeric attrition or that it replicates signals generated by one or more shortened telomeres. We propose that SDB could be a useful tool in the investigation of the molecular pathways involved replicative senescence.

### 6.2 CLONING AND CHARACTERISATION OF HUMAN AND MOUSE TELOMERASE RNA GENE PROMOTER SEQUENCES.

W. Nicol Keith,*[1] Jiang-Qin Zhao[1], Stacey F. Hoare[1], Robert McFarlane[2], Sharon Muir[1], Kenneth Parkinson[2], Donald M. Black[2]. [1]CRC Department of Medical Oncology, University of Glasgow, [2]Beatson Institute, CRC Beatson Labs, Garscube Est., Switchback Rd, Bearsden, Glasgow G61 1BD, UK,

Variation in telomerase activity is correlated with cellular senescence and tumour progression. However, although the enzymatic activity of telomerase has been well studied, very little is known about how expression of telomerase genes is regulated in mammalian cells. We have therefore cloned the promoter regions of the human, (hTR), and mouse, (*terc*), telomerase RNA genes in order to identify the regulatory elements controlling telomerase RNA gene transcription. 1.76kb encompassing the hTR gene promoter region was sequenced, as was 4kb encompassing the *terc* promoter. No significant sequence similarity could be detected in comparisons between human and mouse 5'-regions, flanking the transcribed sequences. However, both the human and mouse telomerase RNA genes are within CpG islands and may therefore be under the regulation of DNA methylation. Transient expression of hTR-reporter gene constructs in HeLa and GM847 cells identified the elements responsible for promoter activity are contained in a 231bp region upstream of the transcriptional start site. Transient expression of *terc*-reporter gene constructs in 3T3 and A9 cells identified the elements responsible for promoter activity are contained in a 73bp region upstream of the transcriptional start site. These studies have implications for our understanding of the signal transduction pathways linking telomere attrition to proliferation, cellular senescence, differentiation and oncogenesis and for the development of novel transcription targeted cancer therapies.

### 6.3

### WITHDRAWN

### 6.4

2,6-DISUBSTITUTEDAMIDO- ANTHRACENE-9,10 DIONES: SMALL MOLECULE INHIBITORS OF HUMAN TELOMERASE. L R. Kelland*[1], S.M. Gowan[1], P.J. Perry[2] and S.Neidle[2]. [1]CRC Centre Cancer Therapeutics and [2]CRC Biomolecular Structure Unit, Institute of Cancer Research, 15 Cotswold Road, Sutton, Surrey SM2 5NG, UK.

Interest in telomerase, a reverse-transcriptase enzyme possessing its own RNA template and responsible for maintaining the repeat telomeric sequences on the ends of chromosomes, has increased substantially in recent years, both in a diagnostic and therapeutic setting. Around 85% of tumours exhibit telomerase expression while expression is absent in the majority of somatic cells; high telomerase activity has been shown to correlate with poor prognosis in some tumour types; telomerase activation has recently been directly shown to be involved in bypassing cellular senescence. Hence, potent and selective inhibitors of telomerase may provide a novel tumour-selective target for anticancer drug design. The telomeric repeat sequence in humans is 5'-TTAGGGn, a guanine-rich sequence capable of forming G-quadruplex four-stranded DNA structures. We have adopted a telomerase-inhibitory strategy aimed at the design of small molecules capable of stabilising G-quadruplexes and thereby preventing the requirement of telomerase for a non-folded telomere DNA primer. A series of compounds based on 2-6 disubstituted amido-anthracene-9,10-dione derivatives, shown to form G-quadruplex complexes with telomeric DNA, have been evaluated for telomerase inhibitory and *in vitro* growth inhibitory properties against human ovarian carcinoma cell lines. The initial biological evaluation of putative inhibitors examined the selectivity of polymerase versus telomerase inhibition by testing the ability of compounds to inhibit *Taq* polymerase in a cell-free assay. While the anthraquinone-based anticancer drugs doxorubicin and mitoxantrone were potent inhibitors of *Taq* polymerase at 10μM, some of the novel disubstituted derivatives exhibited no inhibition at either 10 or 50μM. Moreover, these "polymerase-negative" compounds showed a range in their ability to inhibit human telomerase in an *in vitro* cell-free PCR-based assay (e.g., 50% inhibition at concentrations ranging from 4.5μM for BSU1021 to >50μM for BSU1032). Parallel growth inhibition studies (96h drug exposure, sulforhodamine B assay) have shown the 2-6 disubstituted compounds to be substantially less cytotoxic than doxorubicin (mean $IC_{50}$ of approx 10nM) or mitoxantrone (mean $IC_{50}$ of approx 0.5nM) with $IC_{50}$ values typically in the 0.5 to 5μM range. Interestingly, long-term culture of the A2780 cell line with sub-cytotoxic concentrations of one of the complexes, resulted in detectable telomerase inhibition from 18 days onwards with a maximum inhibition of 40% at 30 days concomitant with a marked decrease in cell growth. These small molecule inhibitors of telomerase represent promising leads worthy of further *in vitro* and *in vivo* antitumour evaluation.

### 6.5

**A NOVEL P53 BINDING SEQUENCE IN INTRON 3 OF THE HUMAN *MDM2* PROTO-ONCOGENE: A PUTATIVE ADDITIONAL P53-DEPENDENT PROMOTER,** H. Liang* and J. Lunec, Cancer Research Unit, University of Newcastle upon Tyne, NE2 4HH, U.K

The *MDM2* proto-oncogene exhibits a complex pattern of expression, producing a number of overlapping transcripts which differ by site of initiation of transcription and by alternative splicing of internal exons. So far two promoters (P1 and P2) have been identified. The P1 promoter is located upstream of exon1 and induces transcripts initiating at the first base of exon 1 in a p53-independent manner. The P2 promoter is located near the 3' end of intron 1 and produces transcripts starting at the third nucleotide of exon 2 in a p53-dependent manner. Since the coding of this gene starts from exon 3, these two promoters would generate transcripts with identical translation potential.

In the process of human *MDM2* genomic sequence mapping, we have identified a promoter-like region near the 3' end of intron 3, including a TATA box (TATAATA) 28 base pairs upstream of exon 3 and also four motifs upstream of this exon, which show a good match to the consensus p53 binding sequence. To test whether there is another p53-dependent promoter in intron 3, by which a transcript lacking part of the p53 binding domain sequence is expected to be generated, a number of the experiments have been performed. Electrophoretic mobility shift assays (EMSA) displayed that the mobility of the DNA fragment in this region is retarded in the presence of p53 and p53 antibody (pAB421) gives a super-shift. The binding of p53 to this DNA fragment was competed out by excess non-labelled specific DNA but not non-specific DNA. Immunoprecipitation/DNA-binding assays also showed specific p53 binding consistent with the EMSA results. In DNA footprinting experiments, TATA binding protein (TBIID) was observed to protect from DNAse digestion in the putative TATA box region. Taking all together, we conclude that p53 binds to intron 3 of the human *mdm2* in a sequence-specific manner . We are carrying further studies, including reporter gene experiments and the identification of truncated transcripts corresponding to this putative promoter.

### 6.6

p53 - INDEPENDENT APOPTOSIS IN HUMAN B CELLS TREATED WITH GENOTOXINS. Mark Wade and Martin J. Allday. Department of Virology and Cell Biology and Ludwig Institute for Cancer Research, Imperial College of Science Technology and Medicine, St Mary's Campus, Norfolk Place, London W2 1PG.

The DNA damage induced by genotoxins is known to activate p53, an established effector of apoptosis in many cell systems. We are currently investigating the responses to a range of genotoxins in a number of B cell lines. Surprisingly, in several Burkitt's lymphoma (BL) cell lines carrying mutant non - functional p53 (defined by its inability to transactivate target genes or induce apoptosis in a heterologous system), apoptosis was induced by a range of genotoxins. Associated with morphological changes such as nuclear condensation and fragmentation, and membrane blebbing, we observe cleavage of poly ADP - ribose polymerase (PARP) but not, however, appearance of a sub - $G_1$ DNA peak in FACS analysis. Initial data from experiments combining TUNEL and cell cycle analysis by flow cytometry suggests this is because apoptosis is initiated from $G_2$/M phase of the cell cycle. Western blot data indicates no change in steady state levels of p53 and members of the bcl - 2 family, including bcl - 2 and bax.

We have also examined similar responses in one of these lines which has been latently infected with EBV. In contrast to its non - infected parental line, we find this convert to be relatively resistant to genotoxin - induced apoptosis as measured by PARP cleavage and FACS analysis. One (or a combination of) the 9 latent EBV gene products protects cells from this p53 - independent pathway. Candidate pathways which may activate this p53 -independent apoptosis induced by DNA damage are currently being investigated.

### 6.7 EFFECTS OF P53 AND P16 TRANSFER IN PANCREATIC CANCER CELLS

P. Ghaneh*[1], M. Humphreys[1], W. Greenhalf[1], N.R. Lemoine[2], J.P. Neoptolemos[1], [1]Dept. of Surgery, Royal Liverpool Hospital, Liverpool, [2]ICRF Molecular Oncology, Hammersmith Hospital, London.

Pancreatic cancer remains relatively resistant to present regimens of chemotherapy and radiotherapy. New and potentially more effective treatments may need to focus on the molecular basis of the disease. Pancreatic cancer displays significant and consistently high levels of loss of function of the tumour suppressor genes p53 (76%) and p16 (82%). Both influence cell cycle control and promote G1 arrest. P53 transactivates the universal cyclin inhibitor p21 and p16 encodes a protein which inhibits CDK4 and 6 thereby preventing hyperphosphorylation of the retinoblastoma protein (pRb) and further progression through the cell cycle. P53 also has a major role in p53-dependent apoptosis. The aim of this study was to replace wild-type p53 and p16 into pancreatic cancer cell lines and assess the subsequent effects on cell growth and cell death. Human pancreatic cancer cell lines BxPc3, Panc1, MIAPACA (all homozygously deleted for p16 and LOH p53), CFPAC1 (wild-type p16 and LOH p53) were used. The cell lines Hep2 (wild-type 53), SW620, and FF1 (wild-type p16 and p53) were used as control cell lines ie. with functional wild-type genes. Transfer of the gene of interest into the cells was achieved using adenovirus vectors expressing either wild-type p53 or p16 under control of a CMV promoter. Cells were grown under routine tissue culture conditions and infection was carried out at a MOI of 50-100. Control infections were carried out using adenovirus expressing β-galactosidase and phosphate buffered saline (PBS) was used for mock infections. Expression of the therapeutic genes was confirmed using PCR, rt-PCR, western blotting and immunofluorescence. The efficacy of gene transfer was assessed using X-gal staining. Cell counts based on trypan blue exclusion were used to calculate cell growth curves for 7 days following infection. Cell cycle distribution was assessed using flow cytometry. Apoptosis was confirmed by DNA laddering and TdT labelling. Transfer of wild-type p53 resulted in growth inhibition rates of 78 - 91% (n=3+3). Transfer of wild-type p16 resulted in growth inhibition rates of 66 - 83% (n=3+3). P16 transfer induced G1 growth arrest in all cell lines resulting in a significantly high G1/S ratio ($p<0.001$) when compared with control infection. The apoptosis index, using TdT labelling, following transfer of p53 was significantly increased ($p<0.004$) when compared with control infections. The transfer of p16 also resulted in a significant increase in apoptosis ($p<0.03$). In conclusion adenoviral-mediated transfer of wild-type p53 and p16 resulted in significant growth inhibition and increased rates of apoptosis in human pancreatic cancer cells. This approach may represent a future therapy for pancreatic cancer.

### 6.8 TP53 AND RELATED PROTEIN EXPRESSION IN PRIMARY AND METASTATIC NEUROBLASTOMA

D A Tweddle*[1,2], A J Malcolm[3], M M Reid[4], M Cole[5], J Lunec[2] and A D J Pearson[1]. Departments of Child Health,[1] Cancer Research Unit[2], Pathology[3], Haematology[4] and Statistics[5] University of Newcastle, NE2 4HH, U.K.

Background Wild-type TP53 has been reported to be overexpressed and abnormally located in the cytoplasm of undifferentiated neuroblastoma.
Aims This study further investigates TP53 and related protein expression in neuroblastomas pre and post chemotherapy (Pre-C and Post-C) and compares primary tumour expression with bone marrow metastases Pre-C.
Methods 30 formalin fixed paraffin-embedded neuroblastoma tumours Pre-C (all stages), 26 Post-C (16 Pre-C and Post-C) and 32 bone marrow trephine biopsies (stage 4) (16 with primary tumour also) were studied by immunocytochemistry for TP53, Ki-67, WAF-1 BCL-2 and BAX. Tumours were scored positive for TP53 if >10% cells immunostained using DO-7 and DO-1 antibodies.
Results In 30 tumours Pre-C, nuclear TP53 positivity was related to adverse clinical outcome for D0-1 ($p<0.0001$); hazard ratio 5.9 (95% CI 1.2-28.7) and D0-7 ($p<0.01$). TP53 expression was also found to be associated with high stage (3 and 4) ($p=0.007$). There was no relationship with any of the other proteins studied, nor was immunostaining for any of these other proteins related to clinical outcome. In 16 cases where primary tumour and bone marrow was studied there were no significant difference in Ki-67, WAF-1 BCL-2 or BAX or DO-1 expression between the 2 sites, however TP53 expression detected by DO-7 was found to be lower in the bone marrow ($p=0.044$) (median difference (MD) 4%). In the 32 bone marrow biopsies there was no relationship between expression of any of the proteins studied and clinical outcome. In the 16 tumours studied Pre-C and Post-C there was a significant reduction in Ki-67 ($p=0.001$, MD 31%) and nuclear TP53 expression (DO-7, $p=0.05$, MD 7.3%, DO-1, $p=0.02$, MD 6.7%) Post-C. In 22/26 tumours Post-C there was evidence of differentiation towards ganglion cells. In 5-50% of these differentiated cells cytoplasmic TP53 was detected with both TP53 antibodies.
Conclusions In neuroblastoma primary tumours nuclear TP53 accumulation is associated with high stage and adverse clinical outcome. A larger study is underway to determine if TP53 is an independent prognostic marker. There was no association betweenTP53 or related protein expression in the bone marrow and clinical outcome. Nuclear TP53 expression falls Post-C and becomes detectable in the cytoplasm of differentiating neuroblastoma where it may have a role in the induction of differentiation.

### 6.9 THE ROLE OF FUNCTIONAL P53 IN CISPLATIN CHEMOSENSITIVITY IN A PANEL OF HUMAN OVARIAN CANCER CELL LINES.

MI Walton*, P Koshy, CJ Medlow, S Sharp, L R Kelland, S Sharp and J Titley. CRC Centre for Cancer Therapeutics, ICR, 15 Cotswold Road, Sutton, Surrey, SM2 5NG, UK.

The tumour suppressor protein p53 is involved in cell cycle checkpoint control and apoptosis. It is the most frequently mutated gene in human cancer but its role in chemosensitivity is unclear. We have determined the functional p53 status of a panel of human ovarian cancer cell lines in order to explore its relationship with cisplatin chemosensitivity. P53 functional status was determined by measuring p53 protein induction and $p21^{WAF1/CIP1}$ and MDM-2 mRNA induction 4h following 5Gy irradiation. G1/S checkpoint integrity was determined by measuring bromodeoxyuridine incorporation 16h following 5Gy. Cisplatin cytotoxicity was measured by 96h sulphorohodamine growth delay assay. Cisplatin uptake was determined using flameless atomic absorbance. Mutations were identified by SCCP and direct PCR sequencing of exons 5,6, 7 and 8 of genomic *P53*. A2780 and CH1 showed marked (7-15-fold) induction of p53 protein, $p21^{WAF1/CIP1}$ and MDM-2 mRNA 4h following irradiation, consistent with a wild type phenotype. PXN94 showed minimal protein induction but 3-4 fold induction of $p21^{WAF1/CIP1}$ and MDM-2 consistent with a a partial wild type p53 function. HX62 showed stable overexpression of p53 protein and no gene induction consistent with a mutant phenotype. Both 41M and SKOV-3 showed no detectable protein expression or induction of $p21^{WAF1/CIP1}$ or MDM-2 and there was no detectable P53 mRNA in either line consistent with a null p53 status. Studies of the G1/S checkpoint supported the above conclusions. Sequencing confirmed that A2780 and CH1 were wild type, that HX62 had a point mutation in exon 8, 41M had a deletion in exon 6. Both PXN 94 and SKOV-3 exhibited wild type sequence in exons 5-8. The cisplatin 2h $IC_{50}$ values were 5.7∓0.9, 2.8∓1.0, 19.1∓5.2, 81.8∓23.4, 5.1∓2.3 and 38.3∓12.6uM (mean∓SD, n=3) for A2780, CH1, PXN94, HX62, 41M and SKOV-3, respectively. There was an indication that cisplatin sensitivity correlated with p53 functional status and was also dependent on drug uptake. BCL-2 expression was detected in two cisplatin sensitive lines (41M and CH1) but none of the others. In conclusion cisplatin chemosensitivity is dependent on several factors including p53 functional status and drug uptake in this panel of human ovarian cancer cell lines.

### 6.10 Onyx-015, an E1B deleted adenovirus, enhances the cytotoxicity to cisplatin and radiation in the functional p53 human ovarian adenocarcinoma cell line A2780.

I Ganly*,R Brown.CRC Dept of Medical Oncology,CRC Beatson Laboratories, Glasgow G61 1BD, Scotland.

Adenoviral E1A increases cytotoxicity to DNA damaging agents such as chemotherapy and radiotherapy by p53 dependent and independent pathways. An E1B attenuated adenovirus,Onyx-015, has been reported to synergistically increase sensitivity of cells with nonfunctional p53 to cisplatin and 5FU (Heise et al;Nature Medicine 1997; 3:639) **AIMS:** (1)To determine if an E1B deleted adenovirus, Onyx-015, would increase cytotoxicity to cisplatin and radiation in the cell line A2780 which has functional p53. (2) To determine if there was any relationship between cytotoxicity and p53 expression and function. **METHODS:** (1) A2780 cells were infected with 0nyx-015 at a MOI of 100pfu/cell and cell lysates made over a time course of 72hours. Expression of p53, p21 and viral E4ORF6 proteins was determined by Western blotting. (2) Clonogenic survival assays were done at a MOI of 1,10 and 100pfu/cell Onyx-015 alone and in combination with either cisplatin ( 1, 5, 10 and 20μM) or radiation( 0.25, 0.5, 1 and 2Gy). Cells were infected for 24hrs and 72hrs before being exposed to cisplatin or radiation. **RESULTS:** (1) Cytotoxicity to cisplatin and radiation was enhanced by Onyx-015 infection. For instance, clonogenic survival with 0nyx-015 alone at a MOI of 100pfu/cell was 0.18, radiation alone of 0.5Gy was 0.65, and Onyx-015 plus radiation was 0.13. Thus clonogenic assays showed cytotoxicity was additive rather than synergistic. (2) Onyx-015 infection produced a dramatic rise in expression of p53 in A2780 which was sustained over 72hours. Expression of p21 was induced for 24hours and then reduced to basal levels by 72hours despite high levels of p53. The reduction in p21 occurred when expression of adenoviral E4ORF6 was maximal. This suggested p53 transcriptional activity was downregulated by E4ORF6 possibly by complex formation to p53. (3) Cytotoxicity to cisplatin and radiation after exposure to Onyx-015 for 24 hours and 72 hours were similar. This suggested there was no correlation between cytotoxicity and Onyx-015 induced effects on p53 transcriptional activity. **CONCLUSION:** Overall, cytotoxicity with Onyx-015 in combination with either cisplatin or radiation is better than each agent alone. Therfore, E1B deleted adenoviruses may be useful adjuvants to chemotherapy and radiation in tumours with functional p53 as well as in tumours with nonfunctional p53.

### 7.1 METHYLATION OF THE MLH1 PROMOTER RESULTS IN LOSS OF MLH1 EXPRESSION AND DRUG RESISTANCE IN OVARIAN TUMOUR CELLS,

G. Strathdee*, M. MacKean and R. Brown, Dept of Medical Oncology, CRC Beatson Laboratories, Glasgow G61 1BD.

Jaffrey R.G.*[1], Marsh S.[1], Pritchard S.C.[1], Johnston P. G.[2], Cassidy J.[1], McLeod H.L.[1]. [1]Oncology Group, Department of Medicine & Therapeutics, University of Aberdeen, AB25 2ZD, UK., [2]Department of Oncology, Queen's University of Belfast, UK.

Drug resistance is a major obstacle to improving the utility of current chemotherapy agents. Thymidylate synthase (TS) inhibitors are very commonly used in the treatment of many cancers including colon, breast, and head and neck. We evaluated the role of genomic instability in three chemotherapy sensitive human carcinoma cell lines and daughter cell lines displaying derived resistance to Tomudex (MCF7-TDX, H630-TDX), or 5-fluorouracil (R10-5FU). Genomic instability between the paired parent and resistant cells was determined by directly incorporating [α-$^{32}$P]dCTP into a PCR-amplified microsatellite reaction, the products separated on a 6.5% polyacrylamide sequencing gel, followed by autoradiography. Microsatellite primers for regions throughout the genome were used, including loci on chromosomes 1p36, 2p, 3p, 5q, 17p13, and 18p11.32. No evidence of genomic instability (0/30 loci) was observed between MCF7 and its resistant clone MCF7-TDX. A high degree of loss of heterozygosity, and allelic instability was observed in H630 and R10 resistant cells. The results in H630 and R10 suggest that microsatellite instability is associated with chemotherapeutic resistance. The putative relationship between genomic instability and resistance to TS inhibitors is currently under investigation. As genomic instability is a common finding in tumour types for which TS inhibitors are used, this may represent a clinically important mechanism of resistance.

### 7.3 EXPRESSION OF DNA MISMATCH REPAIR PROTEINS IN ACUTE LYMPHOBLASTIC LEUKAEMIA AND NORMAL BONE MARROW.

E C.Matheson*, A. G.Hall. LRF Molecular Pharmacology Group, CRU, Medical School, Newcastle-Upon-Tyne, NE2 4HH.

Errors during normal DNA synthesis may produce mismatched base pairs. 6-Mercaptopurine (6MP), given during continuing therapy in acute lymphoblastic leukaemia (ALL), undergoes intracellular activation to give cytotoxic thioguanine nucleotides which are then incorporated into the DNA of dividing cells in place of guanine. Cell death is thought to result from futile attempts at mismatch repair. Previous work has shown that cell lines with a defect in this pathway develop tolerance to incorporated 6-thioguanine bases. In order to investigate the possible relevance of mismatch repair to the chemosensitivty of blasts to 6MP, relative to normal tissues, we have measured the expression of the mismatch repair proteins hMLH1, hMSH2, PMS2 and hMSH6 in blasts from children and adults with ALL and in normal bone marrows, using Western blotting with enhanced chemiluminescence detection and densitometry. Actin was used as a loading control.

Fifty cases of childhood ALL, 22 cases of adult ALL and 7 normal marrows have been studied. Expression of MSH2, and of MLH1 in all but three cases, was detectable in all the blasts studied. Noticeably, expression of MLH1 was not detected in any of the normal marrow samples. MSH2 was detected in 4 of the normal marrows. Expression of PMS2 was not detected in 29 cases of ALL and, like MLH1, was absent from each of the normal marrow samples. In contrast, MSH6 was detected in all of the normal marrows and all but 16 of the cases of ALL. There was no difference in expression between adults and children.

These results may help to explain the relative sensitivity of leukaemic blasts to thiopurines at presentation as compared to normal bone marrow. *This work was supported by the Leukaemia Research Fund*

### 7.4 CHEMOTHERAPY AND MUSCLE-INVASIVE BLADDER CANCER: ASSESSMENT OF P53 IMMUNO-REACTIVITY (IR) AS A PREDICTOR OF RESPONSE.

K.N. Qureshi*[1,2], T.R.L. Griffiths[2], C. Marsh[3], M.C. Robinson[3], R.R. Hall[4], J. Lunec[1] and D.E. Neal[1,2]. [1]Cancer Research Unit, [2]Department of Surgery, University of Newcastle NE2 4HH, [3]Department of Pathology, [4]Northern Cancer Network, Freeman Hospital, Newcastle Upon Tyne NE7 7DN

Studies of patients with muscle-invasive bladder cancer treated by radical cystectomy have generally suggested that tumours with high p53 IR are more likely to have a poor outcome. In contrast, a recent study (Cote *et al*, 1997 Nature 385 124) has suggested that this group is more chemosensitive.

We identified 83 patients with non-metastatic muscle-invasive bladder cancer treated with cisplatin-based systemic chemotherapy. Of these patients, 31 were treated with chemotherapy only; the remainder had further adjuvant treatment in the form of salvage cystectomy or radiotherapy. Paraffin-embedded sections of bladder tumour obtained at transurethral resection prior to chemotherapy were stained for p53 (1801 antibody) using a heat-based antigen retrieval method.

Nuclear staining for p53 was demonstrated in 36/83 (43%) of tumours (range of % p53 positivity: 0-91%). There was no significant difference in cancer-specific survival for patients treated differently. Overall, p53 did not predict complete response (CR), complete and partial response to chemotherapy, or predict time to death from bladder cancer. However, in 48 patients in which complete data was available treated with chemotherapy and subsequent adjuvant therapy, >20% p53 staining was associated with improved survival (P=0.009) with a 5 year survival of 60%; for those with <20% p53 IR the 5 year survival was 21%. Multivariate analysis demonstrated that > 20% p53 staining (P=0.006) and CR (P=0.011) were independent predictors of cancer-specific survival in this group. Of the 48 patients, 17 patients were treated with chemotherapy and subsequent salvage cystectomy. In this subgroup, >20% p53 staining was associated with improved survival (P=0.028). In contrast, p53 was not associated with survival in 27 patients treated with chemotherapy and salvage radiotherapy.

P53 immuno-positivity did not predict response to systemic chemotherapy in muscle-invasive bladder cancer. However, >20% p53 IR may identify a subset of patients who benefit from subsequent salvage cystectomy following chemotherapy.

### 7.5 CHROMOSOMES 7 AND 17 AS PREDICTORS OF DISEASE RECURRENCE AND PROGRESSION IN TRANSITIONAL CELL CARCINOMA OF THE URINARY BLADDER.

ADWatters[1*], SABallantyne[1], JJGoing[2] KMGrigor[3], TGCooke[1]& JMSBartlett[1].University Depts of Surgery[1] and Pathology[2], Glasgow Royal Infirmary, Glasgow, University Dept of Pathology[3], Edinburgh.

Transitional cell carcinoma of the urinary bladder is the fourth commonest cancer in UK males. A particular characteristic of this disease is the high rate of recurrence (80%), which carries a 10-20% risk of progression to muscle invasion following presentation. This heterogeneity implies that a range of genetic lesions are involved. Molecular markers are of great interest as another tool for diagnostic and therapeutic strategies. As technologies improve, these are becoming more viable in clinical settings.
FISH is reliable and reproducible and widely used to study numerical chromosomal aberrations. The significance of chromosomes 7 and 17 copy number in recurrence and progression was investigated in 128 sequential carcinomas from 52 patients with complete follow-up (mean = 4.8 years). Using archival material and alpha satellite probes, results were assessed quantitiatively, a proportion by 3 independent observers. Mean inter observer variation was $\leq$ 10% for either chromosome. No abnormality was observed for chromosomes 7 and 17 in patients who did not recur (n=20). 31% (10/32) of patients who recurred showed an increase in copy number for either chromosome, in their primary tumours (p=0.0082). Of these 7/11 (55%) with recurrent progressive disease demonstrated significant increases in either chromosome in multiple tumours prior to muscle invasion (p=0.0198). This data therefore presents a potential diagnostic molecular marker of recurrence and progression in transitional cell carcinoma.

### 7.6

**EXPRESSION OF CYCLIN DEPENDENT KINASE INHIBITORS IN LARYNGEAL CANCER.**

*Jean-Pierre Jeannon, +James V Soames, θJohn Lunec, *Janet A Wilson & γCharles G Kelly. *Departments of Otolaryngology, +Oral Pathology, γOncology & θCancer Research Unit, University of Newcastle upon Tyne.

**Introduction:** The cyclin dependent kinase inhibitors control the cell cycle and include p21 (WAF1) and p27 (KIP1) [1]. p27 expression is a reliable prognostic indicator in breast and colo-rectal cancers where low levels are associated with poor prognosis [2].
**Aims:** To determine if: (1) p27 is expressed in laryngeal cancer (2) expression relates to prognosis.
**Methods:** A retrospective study on formalin-fixed paraffin embedded tissue randomly chosen. Specimens were immunostained with monoclonal antibody to p27. Scoring was at high power magnification on random fields. Positive, negative and normal controls were employed. High positivity was defined as >50% positive cells per field. Maximum follow-up was 9 years.
**Results:** 50 cases were included in the study, all of which stained positive to p27. Complete case notes were available on 39: 23 T1/T2 glottic, 5 T3/T4 glottic, 5 T1/T2 supraglottic & 6 T3/T4 supraglottic cancers. Low p27 expression was associated with poor survival outcome in early glottic cancer (table 1).

| T1 / T2 | | Mean Survival | |
|---|---|---|---|
| p27 Expression | N | (months) | p=0.05 Mann- |
| Low | 4 | 37 | Whitney U |
| High | 19 | 68 | Test |

**Summary:** We have demonstrated p27 expression in laryngeal cancer for the first time. Low levels of p27 expression appear to be associated with poor prognosis in early glottic cancer.

**References:** 1. Morgan D. The dynamics of cyclin dependent kinase structure. Current Opinion in Cell Biology. 1996 (8): 767-772. 2.Stegg P &Abrams J, Cancer prognostics: Past, present and p27. Nature Medicine 1997 3: 152-154.

### 7.7 PROGNOSTIC SIGNIFICANCE OF STROMELYSIN 3 (ST3) LEVELS INHUMAN BREAST CANCER AND DEMONSTRATION OF NOVEL ST3 EXPRESSION IN METAPLASTIC BREAST CARCINOMA CELLS WHICH HAVE UNDERGONE EPITHELIAL-TO-MESENCHYMAL TRANSITION (EMT),

A Ahmad[1]*, A Hanby[2], EA Dublin[2], RD Rubens[2], DM Barnes[2] and IR Hart[1], [1]Richard Dimbleby Dept of Cancer Research/ICRF, St Thomas' Hospital, London SE1 7EH, [2]ICRF Clinical Oncology Unit, Guy's Hospital, London SE1 9RT.

The matrix metalloproteinase ST3 has been implicated in breast carcinoma progression from its expression in the stromal fibroblasts adjacent to invasive breast carcinoma cells. To assess the prognostic significance of ST3 expression, a mouse monoclonal antibody, raised against the ST3 haemopexin domain, was used to immunostain pre-invasive (28 cases of ductal carcinoma *in-situ*) and invasive (30 of each grade of ductal, 28 lobular and 14 cases of 'metaplastic' carcinoma) breast cancers. In contrast to pre-invasive disease, where only 6/28 DCIS cases expressed ST3, stromal fibroblasts in 72/91 cases (80%) of invasive ductal carcinoma expressed ST3 (p<0.0001). Levels of ST3 expression in these invasive cancers showed no correlation with tumour grade, axillary nodal status or oestrogen receptor status. However, patients with invasive ductal carcinoma exhibiting moderate or strong levels of fibroblastic ST3 expression (n=40) had significantly shorter disease-free survival than those with negative or weak ST3 levels (n=50) ($\aleph^2$= 5.62, p=0.02). Univariate analysis was used to determine the prognostic significance of ST3 levels relative to nodal status, histological grade, tumour size and age. ST3 expression levels were a stronger prognostic factor in predicting disease-free survival than nodal status and of similar prognostic value to tumour grade. Patient subdivision according to nodal status, revealed ST3 level as a strong predictive factor for relapse-free survival in the 53 patients with node postiive breast cancer (($\aleph^2$= 7.01, p=0.0081). Multivariate analysis, including other previously identified prognostic factors, determined that, in node-positive patients, ST3 level was a strong, independent prognostic parameter for disease-free survival (p=0.005).

Immunostaining 14 cases of the metaplastic carcinoma subgroup revealed, for the first time, ST3 protein expression in spindle-shaped neoplastic cells. *In-situ* hybridisation confirmed that these spindle-shaped cancer cells also expressed ST3 mRNA. Antibodies to vimentin and cytokeratin (CAM 5.2) established that many of the ST3-expressing tumour cells co-express fibroblastic and epithelial cell surface markers, indicating that they have undergone a degree of EMT. These data indicate that ST3 has prognostic importance in breast cancer and that the expression of ST3 in tumour cells may contribute to the increased metastatic capability seen in certain 'metaplastic' breast carcinomas.

### 7.8 EVALUATION OF AN ENRICHMENT APPROACH FOR SELECTION OF PROGNOSTIC MARKERS IN NSCLC.

Pritchard, S.C.*[1], Kerr, K.M.[2], Lloret, C.[1], Nicholson, M.C.[2], Cockburn, J.S[2], Jeffrey, R.R.[2], McLeod, H[1] [1]Dept of Medicine & Therapeutics, University of Aberdeen, Foresterhill, Aberdeen, UK, AB25 2ZD. [2]Depts of Path, Clinical Oncology & Surgery, Aberdeen Royal Infirmary, Aberdeen, UK, AB25 2ZD.

Lung cancer is the most common cause of cancer death in the world, accounting for nearly 800,000 deaths per year. In stage II Non Small Cell Lung Cancer up to 50% of patients die within 3 years of surgery, therefore more accurate methods are required to identify patients at risk from disease recurrence. Conventional methodologies for identifying putative markers of prognosis require large numbers of patients to test a small number of markers. We used an 'enrichment' approach to identify genes or genomic regions associated with survival. Patients were selected for the extremes of clinical outcome: 20 patients were alive 3 years post diagnosis and 20 patients had died 2-12 months post diagnosis. Using microsatellite analysis and immunohistochemistry we assessed multiple areas of importance for tumour biology including cell proliferation, apoptosis, invasion and genetic instability. Expression of MMP-1, MMP-2 ,TIMP-2, P53, BCL2 and PCNA were not associated with survival. Our studies showed a high level of microsatellite instability in stage II NSCLC. Loss of heterozygosity (LOH) at 3p14, 3p21, 3p25 and 17p were not associated with survival. LOH at 9p22-23 was associated with better survival and more extensive evaluation of this locus is underway. This data suggests that the use of an enrichment approach can eliminate markers from time consuming and expensive larger studies and provide pilot data for definitive marker analysis.

### 7.9 GLUTATHIONE S-TRANSFERASE THETA FAMILY mRNA EXPRESSION IN HUMAN COLORECTAL CANCER.

K-J.Palmer*., A.C.Gough., I.Srouji., H.Chave. and J.N.Primrose. University Department of Surgery, Southampton General Hospital, Southampton, Hampshire, SO16 6YD.

The Glutathione S-Transferases (GSTs) are a superfamily of enzymes which play an important role in the detoxification of carcinogens and the metabolism of chemotherapeutic agents. There are four main subclasses: alpha, mu, pi and theta. Many of these exhibit a high degree of intervariability in their levels of expression, attributed in part to genetic polymorphisms. As the colon is a major site for carcinogen exposure from the diet and environment, it is important to deduce the expression of these enzymes and the influence of the polymorphisms on colorectal cancer. In this study, we have examined the mRNA expression pattern of the two members of the GST theta family, GSTT1 and GSTT2 in 25 matched colorectal tumour/mucosa pairs.

Total cellular RNA was extracted from matched colorectal mucosa and tumour pairs and subjected to reverse transcription (RT) using an oligo dT primer. RT-PCR was performed using specific oligonucleotide primers to detect GSTT1, GSTT2 and β-actin mRNA. The PCR products were analysed by non-denaturing polyacrylamide gel electrophoresis and visualised by ethidium bromide staining under ultraviolet transillumination. The specificity of the PCR products were confirmed by sequencing.

GSTT1 mRNA was expressed in 80% (20/25) of mucosae and 88% (22/25) of tumour samples and GSTT2 mRNA in 52% (13/25) of mucosae and 84% (21/25) of tumour samples. Increased levels of expression of GSTT2, but not GSTT1, was observed in tumour samples (P=0.004 and P=0.1842 respectively; Wilcoxon Matched Pair Signed Ranked Test). The null GSTT1 genetic polymorphism was found in 12% (3/25) of the population studied which were negative by RT-PCR.

In conclusion, this study suggests that both GSTT1 and GSTT2 mRNA are expressed in normal colonic mucosa and tumour samples. GSTT2, but not GSTT1 gene expression appears to be upregulated in colon cancer which may be important in inherent resistance of colorectal tumours to chemotherapy.

### 7.10 INCREASED EXPRESSION OF CYTOCHROME P450 ISOFORMS IN MOUSE TUMOURS AND THEIR ROLE IN THE ENHANCED ACTIVATION OF THE BIOREDUCTIVE DRUG, AQ4N.

MM Murray*[1], T Robson[1], R Gallagher[1], LH Patterson[2], DG Hirst[1], SR McKeown[1]. [1]Radiation Science Group, School of Biomedical Sciences, University of Ulster, Jordanstown, N. Ireland; [2]Dept of Pharmaceutical Sciences, De Monfort University, Leister, UK.

The CYP superfamily of enzymes, in particular the CYP3A family, play a central role in the metabolism of numerous anticancer drugs. Although CYPs are commonly thought of as oxidases they also have well defined actions as reductases under hypoxic conditions. Indeed, we have evidence that suggests that they are involved in the metabolism of the novel bioreductive drug, AQ4N (1,4-bis-{[2-(dimethylamino-N-oxide)ethyl]amino}5,8-dihydroxyanthracene-9,10-dione).

Freshly excised tumour cells under hypoxia metabolise AQ4N extremely efficiently (*in vitro* $t_{1/2}$ 26 min). However, we[1] have shown that if the tumour cells were cultured under normal conditions for 24h before exposure to AQ4N under hypoxia, no DNA damage was observed although the drug was taken up by the cells; alterations in the expression of CYP 3A4 may be responsible. To study AQ4N (and other CYP-sensitive drugs) *in vitro*, and to increase their metabolism *in vivo* we decided to overexpress a range of CYP isoforms in the RIF-1 tumour cell line.

We have transfectanted the RIF-1 cell line with the LNCX retroviral vector containing the following CYP isoforms CYP1A1. CYP1A2, CYP2C10, CYP2E1 and CYP3A4. Transfectants were isolated by growth in selective medium (G418). The transfectants were relatively stable (>50%) *in vitro* and *in vivo*. Results from growth curves indicate that *in vitro* doubling times for all transfectants were similar in selective and nonselective medium. Furthermore, transfection did not have an appreciable effect on growth rate as compared to the parental cell line.

*In vitro* metabolism studies were used to measure the ability of the transfectants to metabolise AQ4N. Incubations consisted of S9 protein (5 mg/ml), AQ4N (50μM), NADPH (15mM) in phosphate buffer at pH7.4 under nitrogen. The reaction was stopped at various times by the addition of low pH mobile phase. Residual AQ4N was quantified by reverse phase isocratic HPLC using a C8 column with a mobile phase of 80% 0.5M ammonium formate/20% acetonitrile (pH 4.2). All transfectants, except CYP2E1, metabolised AQ4N to a greater extent than the parental line. The drug was most effectively metabolised by the CYP3A4 and 2C10 transfectants, with *in vitro* half lifes of 41 and 43 min respectively.

References:

1 Hejmadi, M.V. et al. Br. J. Cancer, **73**, 499.

### 8.1 CONTROL OF HEMATOPOIETIC STEM CELL RENEWAL AND LINEAGE COMMITMENT BY THE RETINOBLASTOMA TUMOR SUPPRESSOR GENE.

K. Macleod[1] & T. Jacks[2]. [1]Dept. of Molecular and Cellular Pathology, University of Dundee, Dundee DD1 9SY; [2]The Center for Cancer Research, The Massachusetts Institute of Technlogy, Cambridge, USA.

The retinoblastoma tumor suppressor gene encodes a critical cell cycle regulator, pRb, involved in regulating the passage of cycling cells through the G1 phase of the cell cycle. pRb is also required for the establishment/maintenance of cell cycle exit during the differentiation of certain cell types. In particular, loss of Rb function in the developing mouse embryo has been shown to result in failed differentiation in the nervous system, the ocular lens and in fetal liver hematopoiesis.

We set out to investigate the role of Rb in controlling hematopoietic differentiation. In particular we were interested in the role of Rb in regulating stem cell entry into cycle and in determining the lineage commitment of hematopoietic progenitors. We compared the ability of fetal liver tissue from wild-type and Rb mutant embryos to repopulate lethally irradiated adult host mice. While Rb mutant fetal liver can rescue short and medium term hematopoiesis, it failed to repopulate transplanted mice on a long-term basis. These results suggested that the long term repopulating stem cell in Rb mutant fetal liver is defective. Mice rescued with Rb mutant fetal liver died prematurely due to anemia manifest in a very low hematocrit and the absence of erythroid progenitors in the bone marrow and spleen. However, the bone marrow, spleen and liver of these mice was over-populated with mature granulocytes, macrophages and megakaryocytes resulting in splenomegaly and an enlarged liver. The cell cycle profile of putative stem cells and of myeloid progenitors from the bone marrow of mice rescued with wild-type and Rb mutant fetal liver was analysed by FACS. A reduced number of stem cells were detectable in the bone marrow of mice rescued with Rb mutant fetal liver. Furthermore, an increased proportion of these stem cells were in S or G2/M phases of the cell cycle compared to stem cells from wild-type rescued bone marrow. These results suggest that Rb mutant stem cells become depleted in the bone marrow of rescued mice due to an aberrantly high rate of cell cycle entry. Furthermore, there is over-commitment to certain myeloid lineages at the expense of erythroid progenitors, which undergo apoptosis. Bone marrow from Rb mutant rescued mice was not leukemogenic when transplanted into nude mice. In contrast, mice rescued with fetal liver lacking the function of both the Rb and p53 tumor suppressor genes did develop a transplantable leukemia.

### 8.2 ACTIVATED MUTANTS OF THE HUMAN GM-CSF RECEPTOR β SUBUNIT CAUSE MYELOPROLIFERATIVE DISORDERS AND LEUKAEMIA IN MICE.

M.P.McCormack* and T.J.Gonda, Department of Human Immunology, Hanson Centre for Cancer Research, Frome Road, Adelaide 5000, Australia.

To date several activating mutations have been discovered in the common signal-transducing subunit (hβc) of the receptors for human granulocyte-macrophage colony stimulating factor (GM-CSF), interleukin (IL)-3 and IL-5, which enable it to signal in the absence of ligand. Two of these mutations, V449E and I374N, result in single amino acid substitutions in the transmembrane and extracellular domains of hβc, respectively[1]. A third, FIΔ, results in a 37 amino acid duplication in the extracellular domain[2]. We have shown previously that, when expressed in primary murine haemopoietic cells, the extracellular hβc mutants could confer factor independence on cells of the neutrophil and macrophage lineages only. However, the transmembrane mutant, V449E, could confer factor-independence on all cell types of the myeloid and erythroid compartments[3].

In order to study the effects of hβc mutants *in vivo*, we have expressed all three mutants in the haemopoietic system of mice by the technique of bone marrow reconstitution. The extracellular hβc mutants FIΔ and I374N induced a chronic myeloproliferative disorder marked by elevated levels of neutrophils, red blood cells and platelets in the blood. In contrast, the transmembrane hβc mutant V449E induced an acute disorder of blast cell accumulation in the blood, reminiscent of leukemias such as AML. Hence whilst activated hβc mutants are capable of inducing haemopoietic disorders, the type of disorder induced depends on the location of the activating mutation.

1. Jenkins, B.J., D'Andrea, R. and Gonda,T.J. (1995) *EMBO J.* 14: 4276-4287.
2. D'Andrea R, Rayner J, Moretti P, Lopez A, Goodall GJ, Gonda TJ, Vadas MA. (1994) *Blood* 83: 2802-2808.
3. McCormack, M.P. and Gonda, T.J. (1997) *Blood* 90: 1471-1481.

### 8.3

DIAGNOSIS OF NON-HODGKINS LYMPHOMA BY FINE NEEDLE ASPIRATE BIOPSY. AN ANALYSIS OF THE RELATIVE CONTRIBUTIONS OF CYTOMORPHOLOGY, IMMUNOPHENOTYPE AND GENE REARRANGEMENT STUDIES. NJ Mulligan*, M Zucca, B Burke, Gilbride K, C O'Hara. Mallory Institute of Pathology, Boston Medical Center, 784 Mass ave., Boston, MA 02118, USA.

The role of fine needle aspirate biopsy in the diagnosis of lymphoma has traditionally been restricted to staging or detection of relapse. We attempted to reach a rapid and accurate initial diagnosis on aspirated material from lymph nodes using morphology, surface marker detection by flow cytometry and PCR analysis of immunoglobulin gene rearrangements. The relative contributions of each of these diagnostic modalities was analyzed and compared with the final histological diagnoses. The cases consisted of 10 cases of non-Hodgkin's lymphoma, 3 cases of Hodgkin's disease and 5 cases of reactive lymphadenitis. The sensitivities of morphology, flow cytometry and PCR for the diagnosis of non-Hodgkin's lymphoma were found to be 80%, 89% and 70% respectively. The corresponding specificities were 71%, 75% and 80% respectively. When we applied criteria which required concordance between at least two of the three modalities to reach a diagnosis, the sensitivity and specificity for the diagnosis of NHL increased to 90% and 86% respectively. The lymphomas were classified according to the Working Formulation and concordance was found in 78% of the cases between the initial cytological and the final histological diagnosis.

**Conclusions:** Preliminary findings from this ongoing study demonstrate that fine needle aspirate biopsy is a useful tool in the initial diagnosis of non-Hodgkin's lymphoma. Analysis of a combination of morphology, immunophenotype and gene rearrangement studies appears to improve diagnostic accuracy when compared with any of these modalities alone. The impact of molecular marker studies (PCR detection of t11:14 and t14:18) on the accuracy of classification will be assessed as additional cases are accrued.

### 8.4

P53 MEDIATES BREAKDOWN OF THE ANTI-APOPTOTIC PROTEIN BCR-ABL IN K562 LEUKAEMIA CELLS.
A. DiBACCO AND T.G. COTTER, Dept. of Biochemistry, University College Cork, Ireland.

Previous work from this laboratory has demonstrated that Bcr-Abl which is expressed in the CML cell line K562 is a potent inhibitor of apoptosis when those cells are exposed to a variety of cytotoxic agents. In the present set of experiments we demonstrated that when K562 cells, which do not contain a functioning p53 protein molecule, were transfected with a temperature sensitive construct of p53 (ts-p53) they became sensitive to the induction of apoptosis by doxorubucin at the permissive temperature. Normally, untransfected cells are quite resistant to the effects of doxorubucin and we were interested to know how p53 reversed this resistance. Using western blotting we were able to demonstrate that at the permissive temperature p53 mediated a breakdown of Bcr-Abl into two fragments. This piece of data suggested that p53 can induce the proteolytic cleavage of Bcr-Abl by an ICE-like protease. To determine if this was being mediated through activation of caspases and perhaps as a consequence of apoptosis rather than an early event we will use zVAD to inhibit both caspases and apoptosis. These results will indicate if p53 mediated cleavage of Bcr-abl is an early event in the apoptosis pathway and if it precedes caspase activation. Using immunoprecipitation we were able to demonstrate a direct interaction between p53 and Bcr-abl. The significance of this last piece of information is still unclear.

This work was supported by Fobairt and The Irish Cancer Society.

KEY WORDS : p53, Bcr-Abl, apoptosis

### 8.5

THIOPURINE METHYLTRANSFERASE GENOTYPE AND PHENOTYPE IN NORMAL SUBJECTS AND PATIENTS WITH ACUTE LEUKAEMIA S. Coulthard,* C. Howell, J. Robson and A.G.Hall, LRF Molecular Pharmacology Group, CRU, Medical School, Newcastle, NE2 4HH

Acute lymphoblastic leukaemia (ALL) is the most common malignancy of childhood. Although current treatment results in long term survival in over 70% of cases there is evidence that as many as 50% could have been cured using a less complex regime with a lower incidence of long term side effects. In previous studies it has been found that thiopurines given as part of continuing therapy are key agents in preventing relapse. However optimal administration during continuing therapy is often not achieved. Variations in the level of thiopurine methyltransferase (TPMT) activity appear to be a major determinant of the extent of thiopurine metabolism. This is partially due to the presence of mutated TPMT genes in approximately 11% of the population. There have been seven mutation sites reported to date which result in reduced TPMT activity, (Otterness *et al* 1997 Clin. Pharmacol. Ther. 62 60). We have screened 97 normals for mutations at the three best characterised loci, position 238, 460 and 719 (Krynestski, *et al* 1996 Pharmacogenetics 6 279, Szumlanski *et al* 1996 DNA and Cell Biology 15 17, Tai *et al* 1996 Am J. Hum. Genet. 58 694). The normal sequence is designated *1, mutations at both 460 and 719*3A, mutated at 460 alone *3B and mutated at 719 alone *3C. In all we have identified 89 homozygous for the wild type gene (TPMT*1/*1) and 8 heterozygous (7 TPMT *1/*3A and 1 TPMT *1/*3C). The mean enzyme activity level was significantly higher in red blood cells in the group of homozygotes (19.1U/$10^8$ erythrocytes) than in heterozygotes (9.1U/$10^8$ erythrocytes, $p<0.0001$).

In a separate study of 60 cases of ALL and AML in which lymphoblast, rather than erythrocyte, TPMT activity was compared with genotype we have shown that the level of activity in homozygotes was significantly lower than in heterozygotes (0.15 v 0.32 nU/mg protein, $p<0.008$). The level of homozygous TPMT activity in AML patients was compared to that in ALL patients and was found to be significantly higher in the AML group (0.37nU/mg protein v 0.27nU/mg protein, $p<0.02$). These results report blast activity and genotype comparisons for the first time and suggest that both genotype and transcriptional factors influence TPMT activity in leukaemic cells. *This work was supported by the Leukaemia Research Fund.*

### 8.6

EXAMINATION OF THE POTENTIAL OF ANTISENSE STRATEGIES IN THE TREATMENT OF CML

**Mc Elwaine S*,Gowing H[1],Lawler M[1],Hollywood DH[2],Cotter TC[3],McCann SR[1]**

**Depts. Haematology[1], Oncology[2], TCD, Dept Biochemistry[3],UCC Ireland.**

In Chronic Myeloid Leukaemia (CML), the generation of a leukaemia specific RNA (bcr-abl) results in a leukaemia specific protein (p210) which confers an anti-apoptotic phenotype on CML cells. The expression of this chimeric gene is central to the pathogenesis and progression of this leukaemia. This leukaemia specific RNA provides an attractive target for antisense studies. However RNA secondary structure may limit this approach and sequence dependent rather than sequence specific effects have been observed. In an effort to overcome these potential problems, we have chosen to downregulate bcr-abl with the use of a sequence specific antisense oligodeoxynucleotide (ODN) directed against the translational start site of the BCR gene as we feel this may lead to a more effective suppression of bcr-abl expression. The nuclease resistance of two backbone chemistries (phosphodiester and phosphorthioate) has also been compared in vitro. ODNs with the phosphodiester chemistry were rapidly degraded in the presence of FCS, whereas the phosphorothioates exhibited minimal degradation at the 3' end of the ODN; however this was not significantly increased over the course of the incubation period and intact 18mer was present at 72 hours. The presence of CML cells did not increase the degradation of the phosphorthioate ODNs when compared to a cell free control. In vitro studies in CML cell lines (K562, LAMA 84, KYO1, BV 173) have indicated that phosphorothioate ODNs complimentary to the start site can enter the cell successfully (as judged by fluorescent microscopy and FACS analysis of 6-FAM labelled ODNs), are stable in CML cells for > 72 hours and can bind to their target sequence. The uptake of these fluorescently labelled ODNs can be improved with permeabilising agents such as saponin as observed in K562 cells by FACScan analysis, also the potential for using cationic lipids may facilitate the uptake of ODNs with minimal toxicity. We are also examining the disribution and significance of the BCR protein expression in normal cells. Normal BCR protein has been detected in a number of haemopoietic lineages as yet a role for BCR has to be fully elucidated. Initial data generated from a long term cobblestone area forming cell assay (CAFC) indicates that incubating normal bone marrow MNC with a dose of 0-10mM antisense and nonsense ODNs for 24 hours prior to plating does not affect there ability to proliferate and form cobblestone forming areas, thus supporting this approach as a potential purging strategy. This observation and the effect of antisense on CML cell lines is currently being investigated in order to understand the effect of downregulating bcr-abl on the phenotype of the CML cell.

### 8.7 ABSENCE OF HHV8/ KSHV DNA IN PERIPHERAL BLOOD AND BONE MARROW HARVESTS FROM PATIENTS WITH MULTIPLE MYELOMA.

N.Gardiner*[1], W. Livingstone[1], P.Simmonds[2], M.Lawler[1], S.R.McCann[1], P.V. Browne[1]. [1]Dept Haematology, St James Hospital, Dublin &[2]Dept.Medical Microbiology, Edinburgh Medical School.

Human herpesvirus 8 (HHV-8), also known as Kaposi's Sarcoma related herpesvirus (KSHV), has been implicated in Kaposi's sarcoma, Castleman's disease and primary effusion lymphomas (PEL). Rettig *et al* have recently demonstrated the presence of HHV-8 in bone marrow biopsies and cultured stromal cells from patients with multiple myeloma (MM). They propose that HHV-8 may influence the switch from MGUS to myeloma through the secretion of viral IL-6 from infected bone marrow dendritic cells. Autologous bone marrow (BM) and more recently mobilised peripheral blood stem cells (PBSC) are routinely used to support high dose chemotherapy in MM. It is possible that these harvests contain HHV-8. We therefore examined the leukapheresis and bone marrow harvests from 9 MM patients for the presence of HHV-8 DNA using a sensitive nested PCR assay. BM was collected in steady state haemopoiesis and PBSC were collected after mobilisation with cyclophosphamide + G-CSF. DNA was extracted from frozen samples of 20 leukapheresis products and 7 bone marrow harvests. The nested PCR assay (2 x 30 cycles) yields a second round PCR product of 423 bp with a sensitivity of $10^{-4}$- $10^{-5}$ using control DNA from a patient with Kaposi's sarcoma. Nested PCR for HHV8-8 DNA was negative in 20/ 20 PBSC products and 7/7 autologous BM harvests from 9 MM patients.The integrity of the extracted DNA was confirmed using PCR primers for the Prothrombin gene (507bp). In conclusion, using a highly sensitive nested PCR assay, no HHV-8 DNA was detected in PBSC and BM products for clinical use.

1. Rettig M. et al, Science : 276; 1851-1854, (1997).

### 8.8 INVESTIGATION OF THE MOLECULAR DEFECT CAUSING MONOCYTE ESTERASE DEFICIENCY.

B.R.Corr*[1], P.C.Winter[2], G.M.Markey[1] & T.C.Morris[1]. Haematology Dept., [1]Belfast City Hospital, Belfast & [2]Royal Victoria Hospital, Belfast, N. Ireland.

Subjects with familial monocyte specific esterase deficiency have a relative risk of 6.6 (confidence limits 1.6, 28.3) for developing non-Hodgkins lymphoma (Markey *et al* J. Leukocyte Biology Supplement 1995 A147). Extracts from their peripheral blood monocytes do not show the series of active isoenzymes demonstrable in monocytes from esterase positive subjects using isoelectric focusing on polyacrylamide gels and non-specific esterase staining.

Monocyte carboxylesterase is specific to cells of monocyte/macrophage lineage. The sequence of the gene for monocyte specific esterase has been established using a cDNA cloned from the monocytic cell line U-937 by Zschunke *et al* (Blood 1991 78(2) 506).

In our study, primers to this sequence were designed and used to perform RT-PCR on RNA extracted from peripheral blood monocytes purified by ficoll centrifugation and miniMACS (Miltenyi Biotech) enrichment - the latter being an immunomagnetic (CD14-labelled magnetic microbeads) procedure.

The 1623bp RT-PCR product obtained from purified esterase positive monocytes was sequenced and the sequence conformed to that of HMSE 1.

To date the RT-PCR products from three esterase negative subjects have been sequenced and the active site and transmembrane regions of the gene have shown no abnormalities. However, a consistent Pro-Leu change in the intervening region has been demonstrated in one of the MED subjects.

This study has shown that RNA is being transcribed from the HMSE 1 gene in monocyte esterase deficiency providing the first step in elucidating the molecular mechanism of MED.

### 8.9 SOMATOSTATIN RECEPTOR EXPRESSION IN LYPHOMA OF MUCOSA ASSOCIATED LYMPHOID TISSUE (MALT).

M.Raderer[1*], J. Valencak[1], A. Kurtaran[2], J. Drach[1], T. Pangerl[2], M- Hejna[1], F. Pfeffel[3], A. Chott[4] and I. Virgolini[2]. Dept. Of Internal Med I[1] and IV[3], Nuclear Medicine[2] and Pathology[3], University of Vienna, Wahringer Guertel 18-20, A-1090 Vienna

Extranodal lymphoma of the MALT– type is a distinct pathologic entity with a usually indolent clinical course, which has been classified as marginal zone lymphoma according to the REAL-classification. The majority of cases arise in the stomach and are thought to be related to the presence of Helicobacter pylori, but also extragastric locations are frequently encountered. While the optimal treatment of such patients remains a matter of debate at the moment, there is widespread consensus that exact staging including endosonography, CT-scanning, endoscopy and bone marrow biopsy is mandatory. We report the results of a study evaluating the role of somatostatin receptor (SSTR) imgaging using $^{111}$In-DTPA-D-Phe1-octreotide ($^{111}$In-OCT) in 20 patients with MALT lymphoma, which displays a high affinity to SSTR-subtypes 2 and 5. In addition, we have investigated the SSTR-subtype expression in 4 human MALT-lymphoma specimens (2 gastric and 2 extragastric). All patients included in the study had histologically verified MALT-lymphoma: 6 had low-grade extragastric manifestations ( 1lymphoma of the lung, 2 conjunctival lymphomas, 1 breast and 2 cases with supraclavicular lesions), 4 had stage I and 2 had stage II disease. 14 patients presented with gastric lymphoma (8 high-grade and 6 low-grade), 5 had stage II and 9 stage I tumours. All patients were injected $^{111}$In-OCT before initiation of therapy, and results of gamma camera imaging were compared to conventional imaging including CT and endosonography. 19 patients were considered evaluable, 1 patient with gastric lymphoma was excluded due to the additional presence of CLL. All 6 patients with extragastric lymphoma had positive scans, while only 1 patient with gastric lymphoma had slight focal tracer uptake in the epigastric area, which was rated as unspecific. The remaining 12 patients had negative scans irrespective of low- or high-grade histology. In vitro results also revealed a different pattern of SSTR-subtype expression in extragastric and gastric MALT-lymphomas. These results indicate that extragastric MALT-lymphomas can be readily visualized by SSTR-imaging using $^{111}$In-OCT and might be potential targets for therapy with SST-analogues. In contrast, gastric MALT-lymphomas do not appear to express relevant amounts of respective SSTR. Our findings suggest a difference between extragastric and gastric MALT-lymphomas on the cellular level despite a well established morphologic and immunohistochemical uniformity.

### 8.10 A FLOW CYTOMETRIC ANALYSIS OF CYTOKINE EXPRESSION IN PERIPHERAL BLOOD MONONUCLEAR CELLS FROM COLORECTAL CANCER PATIENTS.

K.M. Gaskell, J.Greenman and J.R.T., Monson. University of Hull Academic Surgical Unit, Castle Hill Hospital, Cottingham, East Yorks., HU16 5JQ.

INTRODUCTION: It has been suggested that patients with malignant disease may exhibit immunosuppression. Changes in cytokine responses could represent an important aspect of immunosuppression in these patients. Flow cytometry protocols have recently been devised which allow the analysis of intracellular antigens from specific cell populations within a heterogeneous mix. This technique is quick and provides a quantitative assessment of cells expressing a specific antigen of interest via measurement of fluorescence intensity.

AIM: To compare levels of cytokine expression in peripheral blood mononuclear cells (PBMC) from colorectal cancer patients and healthy controls, using flow cytometry.

METHODS: PBMC were isolated from 7 colorectal cancer patients and 11 healthy control subjects. Each sample was divided into two parts. One part remained untreated while the other part was stimulated with phorbol 2-myristate 13-acetate and ionomycin for 2 hours in the presence of monensin. Cells were fixed with paraformaldehyde, permeabilised with saponin and labelled with specific fluorescent directly-conjugated anti-cytokine antibodies. Cytokine-producing cells were analysed on a FACSCalibur flow cytometer (Becton Dickinson). Gating on forward scatter and side scatter was carried out to define the lymphocyte and monocyte populations. Markers were set on fluorescence histograms so that only 3-5% of unstimulated cells were positive. The number of stimulated lymphocytes and monocytes which fell within this positive region was then recorded. Medians and interquartile ranges were calculated for the percentage of stimulated lymphocytes and monocytes in each condition. The data for each cytokine was analysed using a Mann-Whitney U test (two-tailed).

RESULTS: Statistical analysis demonstrated a highly significant ($p<0.01$) increase in the number of cells producing TNF-$\alpha$ in colorectal cancer patients. The data also indicated a trend towards an increase in cells producing IFN-$\gamma$, IL-12 and TGF-$\beta$ in cancer patients, and a decrease in cells producing IL-10, but these differences did not reach statistical significance. Numbers of cells producing IL-2 were only minimally increased in tumour patients.

DISCUSSION: The data displays clear trends in which the numbers of cells producing TNF-$\alpha$, IFN-$\gamma$ and IL-12 are elevated in patients with colorectal cancer, as compared with healthy controls. In the case of TNF-$\alpha$ the elevation was found to be highly significant. These 3 cytokines are known to drive a $T_H1$ type of immune response in which cell-mediated cytotoxic reactions are promoted. For the group of cancer patients tested therefore it appears that a $T_H1$-like response is in operation. Furthermore, numbers of cells producing IL-10 were found to be decreased in colorectal cancer patients as compared with healthy controls. This also represents a favourable anti-tumour response since IL-10 is inhibitory to $T_H1$-like responses. Further studies investigating larger numbers of cancer patients are required to confirm the significance of these results.

CONCLUSION: Present findings suggest that inappropriate cytokine expression may not be a major factor in the ineffectiveness of the immune response against colorectal cancer. We hypothesize that failure of an effector function downstream of cytokine production is responsible for the inability to control the tumour.